# Targeted Synthesis of 3,3′-, 3,4′- and 3,6′-Phenylpropanoid Sucrose Esters

**DOI:** 10.3390/molecules27020535

**Published:** 2022-01-15

**Authors:** Wong Pooi Wen Kathy, Li Lin Ong, Surabhi Devaraj, Duc Thinh Khong, Zaher M. A. Judeh

**Affiliations:** 1 School of Chemical and Biomedical Engineering, Nanyang Technological University, 62 Nanyang Drive, N1.2–B1-14, Singapore 637459, Singapore; pwong003@e.ntu.edu.sg (W.P.W.K.); long008@e.ntu.edu.sg (L.L.O.); surabhi.devaraj@gmail.com (S.D.); thinh@smart.mit.edu (D.T.K.); 2 Institute of Health Technologies, Interdisciplinary Graduate School, Nanyang Technological University, Singapore, 61 Nanyang Drive, ABN-02b-07, Singapore 637335, Singapore

**Keywords:** phenylpropanoid sucrose esters, natural products, orthogonal protection, selective acylation of sugar, antidiabetic compounds

## Abstract

In this study, we report on an orthogonal strategy for the precise synthesis of 3,3′-, 3,4′-, and 3,6′-phenylpropanoid sucrose esters (PSEs). The strategy relies on carefully selected protecting groups and deprotecting agents, taking into consideration the reactivity of the four free hydroxyl groups of the key starting material: di-isopropylidene sucrose **2**. The synthetic strategy is general, and potentially applies to the preparation of many natural and unnatural PSEs, especially those substituted at 3-, 3′-, 4′- and 6′-positions of PSEs.

## 1. Introduction

**Figure 1:**
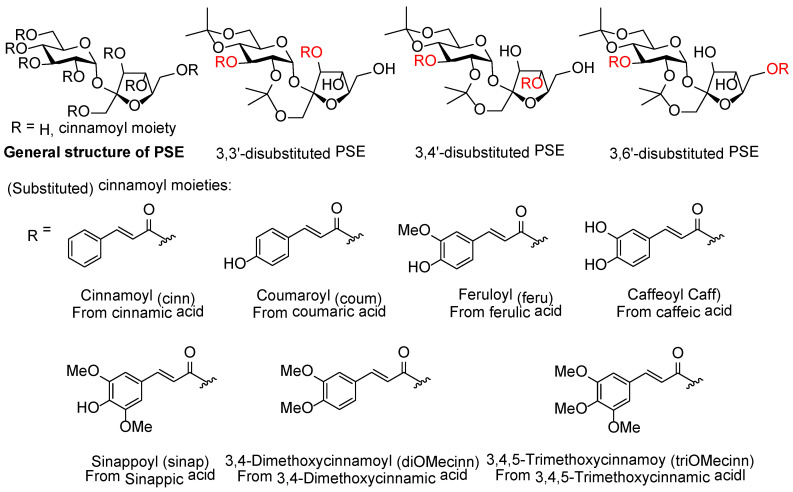
General structure of PSE and the targeted 3,3′-, 3,4′-, and 3,6′-di-substituted PSEs.

Phenylpropanoid sucrose esters (PSEs, Figure 1) are plant-derived compounds and possess many biological activities, including inhibitory activities against both α-glucosidases and α-amylase [1]. For example, the PSEs Lapathoside D, Lapathoside C, Hydropiperoside, Vanicoside B, and Diboside A showed variable levels of inhibitory activities against α-glucosidase and α-amylase [2,3,4,5]. Recently, we proposed PSEs as promising alpha-glucosidase inhibitors (AGIs) that have a better side effect profile than commercial AGIs [6,7,8,9,10]. AGIs possess an excellent efficacy and safety record, and are represented by the commercial drugs acarbose (the gold standard), voglibose, and miglitol. They function by inhibiting the α-glucosidase enzymes responsible for hydrolyzing carbohydrates in the small intestine, thereby restricting its absorption into the bloodstream [11]. However, commercial AGIs cause serious gastrointestinal (GI) side effects such as flatulence, abdominal distension, and diarrhoea [12]. The side effects hamper patient compliance and acceptability, and limit the wide use of these effective drugs [13]. Based on a preliminary structure–activity relationship (SAR) study [6,9,10], we demonstrated that their in vitro inhibition of α-glucosidase and α-amylase depended on the type, number, and position of the phenylpropanoid moieties on the sucrose core and the presence/absence of the di-isopropylidene bridges (Figure 1). The study also identified *O*-3 substituents as favorable for increased level of inhibition of α-glucosidase [6,9,10]. Additionally, the di-isopropylidene bridges favorably reduce the inhibition of α-amylase and minimally affect the inhibition of α-glucosidase [6,9,10]. These results were further corroborated by silico docking and molecular modelling studies. Furthermore, we demonstrated the efficacy of a tetrafeuloyl PSE in a mouse model and found it to be as effective as acarbose in controlling the rise in post-prandial blood glucose levels. The reduction in the inhibition of α-amylase was shown to reduce the side effects of AGIs [14,15]. However, there is a lack of comprehensive and systematic SAR studies identifying the contribution of the type, number, and position of the phenylpropanoid moiety to the inhibition activity. This can be attributed to difficulties in synthesizing PSEs with the precise structures needed for such SAR studies. Previously, we synthesized PSEs through a simple acylation reaction [16,17]. Direct synthesis of substituted PSEs by reacting 2,1′:4,6-di-*O*-diisopropylidene sucrose 2 with substituted cinnamoyl chlorides gave mixtures of differently acylated products depending on the reaction conditions, concentration, and the type of substituted cinnamoyl chloride used, which compromised the yields and complicated the purification process [16,17]. This process cannot be used to synthesize the precisely substituted PSEs required for comprehensive SAR studies.

Herein, we report on the precise synthesis of 3,3′-, 3,4′-, and 3,6′-di-substituted PSEs as potential AGIs. The strategy is designed to also be applicable to other natural and unnatural PSEs.

## 2. Results and Discussion

The inhibition of α-glucosidase and α-amylase varies depending on the type and position of the (substituted) cinnamoyl moieties on the sucrose core (Figure 1) [1,2,3,15]. Therefore, the targeted 3,3′-, 3,4′-, and 3,6′-di-substituted PSEs are designed with commonly found (substituted) cinnamoyl moieties in natural PSEs, including cinnamoyl, coumaroyl, feruloyl, caffeoyl, sinapoyl, 3,4-dimethoxycinnamoyl, and 3,4,5-trimethoxy cinnamoyl moieties (Figure 1). The di-substituted PSEs are designed with di-isopropylidene bridges since they can have a beneficial effect through the reduction of α-amylase inhibition, which is believed to reduce GI side effects [12].

## 3. Synthesis of 3,3′-di-Substituted PSEs

**Scheme 1:**
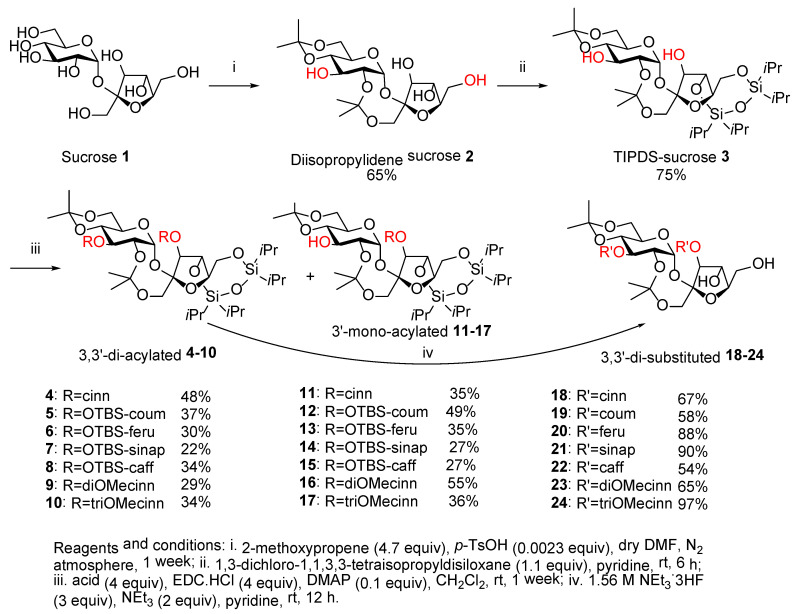
Synthesis of 3,3′-di-substituted PSEs.

The synthesis of 3,3′-di-substituted PSEs began with the reaction of sucrose **1** with 2-methoxypropene to give di-isopropylidene sucrose **2** in 65% yield, following the process of Falkenhagen et al. (Scheme 1) with some modifications [16,17,18]. The dual protection of the 4′-OH and 6′-OH of di-isopropylidene **2** was accomplished using 1,3-dichloro-1,1,3,3-tetraisopropyldisiloxane to give TPDIS-sucrose **3** in 75% yield [19]. Subsequently, Steglich esterification of **3** with cinnamic acid, OTBS-coumaric acid, OTBS-ferulic acid, OTBS-sinapic acid, OTBS-caffeic acid, 3,4-dimethoxycinnamic acid, and 3,4,5-trimethoxycinnamic acid (Figure 1) gave the corresponding 3,3′-di-acylated products **4**–**10** in 22–48% yield, along with the 3′-mono-acylated counterparts **11**–**17** [12] in 27–49% yield (Scheme 1). The formation of 3′-mono-acylated compounds **11**–**17** is attributed to the higher nucleophilicity of 3′-OH in comparison to 3-OH [16,17]. Attempts to increase the yield of 3,3′-di-acylated products **4**–**10** by using higher equivalents of the acids and increasing the reaction time and temperature were unsuccessful. Finally, removal of the protecting TBS and/or TIPDS groups using NEt_3_.3HF gave the corresponding 3,3′-di-substituted PSEs **18**–**24** in 54–97% yield (Scheme 1).

## 4. Synthesis of 3,4′-di-Substituted PSEs

**Scheme 2:**
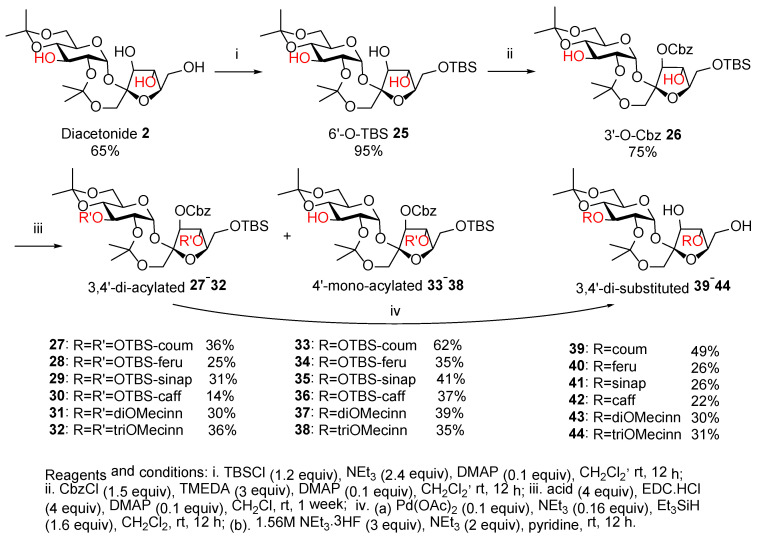
Synthesis of 3,4′-di-substituted PSEs.

The precise synthesis of 3,4′-di-substituted PSEs is shown in Scheme 2. Since the primary 6′-OH of di-isopropylidene sucrose **2** is the most nucleophilic, it was protected first using *tert*-butyldimethylsilyl chloride (TBSCl); the reaction gave 6′-*O*-TBS **25** in 95% yield. Subsequent protection of 3′-OH with benzyl chloroformate (*CbzCl*) gave 3′-*O*-Cbz **26** in 75% yield. 3′-*O*-Cbz **26** was obtained as the major product since 3′-OH is the most reactive among the three OH groups of 6′-*O*-TBS **25** due to fewer steric effects. Next, Steglich reaction between 3′-*O*-Cbz **26** and cinnamic acid, OTBS-coumaric acid, OTBS-ferulic acid, OTBS-sinapic acid, OTBS-caffeic acid, 3,4-dimethoxycinnamic acid, and 3,4,5-trimethoxycinnamic acid gave the corresponding 3,4′-di-acylated products **27**–**32** in 14–36% yield, along with 4′-mono-acylated products **33**–**38** [12] in 35–62% yield, respectively (Scheme 2). Again, attempts to increase the yield of 3,4′-di-acylated products **27**–**32** by increasing the acid equivalents as well as the reaction temperature and time were unsuccessful. In this case, the 4′-OH proved to be more nucleophilic than the 3-OH, as indicated by the formation of 4′-mono-acylated products in high yield. Subsequently, removal of the Cbz protecting groups using Pd(OAc)_2_ and the TBS protecting groups using NEt_3_.3HF gave 3,4′-di-substituted PSEs **39**–**44** in 22–49% yields over two steps (Scheme 2).

## 5. Synthesis of 3,6′-di-Substituted PSEs

**Scheme 3:**
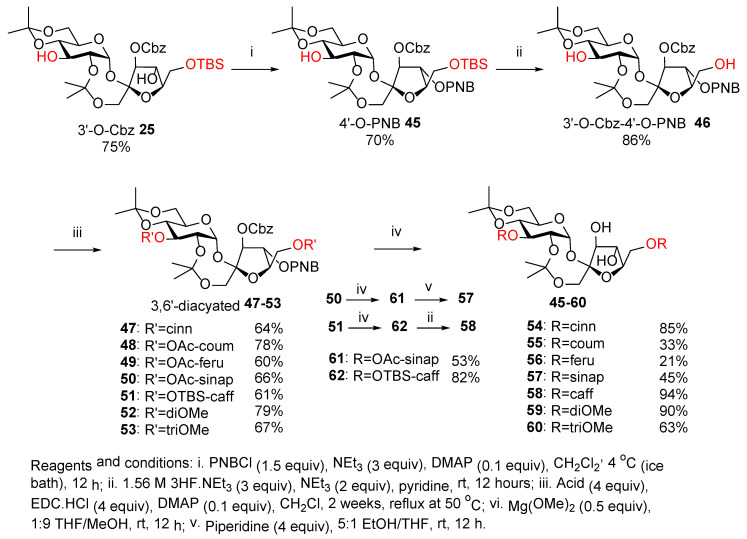
Synthesis of 3,6′-di-substituted PSEs.

The synthesis of 3,6′-di-substituted PSEs began with protecting the 4′-OH group of 6′-*O*-TBS **25** with *p*-nitrobenzoyl chloride (PNBCl) to give 4′-OPNB **45** in 70% yield (Scheme 3). Removal of the TBS protecting group of **45** using NEt_3_.3HF gave compound **46**. Subsequent Steglich reaction between compound **46** and cinnamic acid, acetyl-coumaric acid, acetyl-ferulic acid, acetyl-sinapic acid, OTBS-caffeic acid, 3,4-dimethoxycinnamic acid, and 3,4,5-trimethoxycinnamic acid gave the corresponding 3,6′-di-acylated compounds **47**–**53** in 66–79% yield, along with traces of other compounds thought to be the mono-acylated counterparts. In this case, the yields were much higher in comparison to 3,3′- and 3,4′-di-acylated compounds due to higher nucleophilicity and less steric hindrance of the primary 6′-OH. We purposely protected coumaric, ferulic, and sinapic acids with acetyl groups rather than the usual TBS since these groups can be removed simultaneously in one step during the removal of the Cbz and PNB groups using Mg(OMe)_2_. Acetylation of caffeic acid posed problems, so in this case, OTBS-caffeic acid was used. Finally, simultaneous removal of the Cbz, PNB, and acetyl groups of **47**–**49**, **52** and **53** with Mg(OMe)_2_ gave 3,6′-di-substituted PSEs **54**–**56**, **59**, and **60** in 21–90% yield, respectively (Scheme 3). During the reactions, TLC showed two to three spots indicating stepwise deprotection; the reaction was deemed complete when only one spot was observed. However, while Mg(OMe)_2_ removed the Cbz and PNB groups of 3,6′-diacylated **50**, it failed to remove its acetyl group and gave compound **61** in 53% yield. Piperidine successfully removed the acetyl group of compound **61** to give the required 3,6′-di-substituted PSE **57** in 45% yield. In the case of 3,6′-diacylated **51**, Cbz and PNB groups were removed using Mg(OMe)_2_ to give compound **62** in 82% yield, which upon removal of its TBS groups using NEt_3_.3HF gave 3,6′-di-substituted PSE **58** in 94% yield (Scheme 3). Copies of the [1] H NMR, [13] C [1] NMR, and 2D NMR spectra of the synthesized compounds are available online (Supplementary Materials).

The strategy took advantage of the slight differences in reactivates of the four OH groups on the key compound di-isopropylidene sucrose **2**. The above routes gave the required PSEs with the correct substitution patterns at 3,3′-, 3,4′- and 3,6′- positions, as confirmed via 2D NMR experiments (see supporting information).

The synthesized compounds in this work will decipher the role of the O-3 substituents in inhibition through comprehensive SAR studies.

## 6. Conclusions

We successfully developed a precise synthesis of 3,3′-, 3,4′-, and 3,6′-di-substituted PSEs using an orthogonal protection/deprotection strategy. The synthetic strategy has a wider application for the preparation of many natural and unnatural PSEs, especially those substituted at O-3, O-4′, and O-6′ positions such as Helonioside A, Lapathoside C, and Lapathoside D. Future work will investigate the synthesized PSEs as part of a comprehensive SAR study to develop lead antidiabetic AGIs with a better side effect profile.

## Data Availability

Not applicable.

## Supplementary Materials

The following supporting information can be downloaded. Copies of the [1] H NMR, [13] C [1] NMR, and 2D NMR spectra of the synthesized compounds are available online.

## Sample Availability

Samples of the compounds are not from the authors.
