# Supplementary material for: Targeted Synthesis of 3,3′-, 3,4′- and 3,6′-Phenylpropanoid Sucrose Esters
Source: Molecules. 2022 Jan 15;27(2):535. doi: 10.3390/molecules27020535 (PMC8780461; doi:10.3390/molecules27020535)
Supplement: Supplementary file 1 [file molecules-27-00535-s001.zip › molecules-1543609-supplementary.pdf]

Supporting Information

Targeted Synthesis of 3,3′-, 3,4′- and 3,6′-Phenylpropanoid Sucrose Esters

Wong Pooi Wen Kathy,[1,2] Li Lin Ong,[1,2] Surabhi Devaraj, [1] Duc Thinh Khong,[1] Zaher M. A. Judeh[1*]

[1] School of Chemical and Biomedical Engineering, Nanyang Technological University, 62 Nanyang Drive, N1.2–B1-14, Singapore 637459

[2] Institute of Health Technologies, Interdisciplinary Graduate School, Nanyang Technological University, Singapore, 61 Nanyang Drive, ABN-02b-07, Singapore 637335

Tel.: +65-6790-6738; fax: +65-6794-7553; e-mail: zaher@ntu.edu.sg

Table of Contents:

1.	Materials and methods	2
2.	Synthesis of compounds **2-62**	2-33
3.	^{1}H NMR, ^{13}C NMR and COSY spectra of compounds **3, 25, 26, 45, 11-17, 33-38, 18-24, 23, 43,** and **59**	34-58
4.	References	59

1. Materials and methods

The chemical reagents were purchased from Sigma-Aldrich, Alfa Aesar and Acros and were used as received without further purification. IR spectra were obtained using a Bruker FT-IR spectrometer. ^{1}H NMR spectra were recorded using a Bruker Avance DPX 300 at 300 MHz. ^{13}C NMR spectra were recorded using a Bruker Avance DPX 300 at 75.47 MHz. Samples were measured using deuterated chloroform $CDCl_3$, unless indicated. ^{1}H NMR multiplicities were designated as singlet (s), doublet (d), doublet of doublet (dd), triplet (t), quartet (q), multiplet (m). Waters Q-TOF Premier mass spectrometer (ESI) was used to obtain high resolution mass spectra. Analytical TLC was performed using Merck 60 F254 precoated silica gel plates (0.2 mm thickness) and visualized using UV at 254 nm or stained using *p*-anisaldehyde solution and heated. *p*-Anisaldehyde staining solution is prepared from scratch with *p*-anisaldehyde, acetic acid, sulfuric acid and ethanol.[1] Column chromatography was performed using Merck silica gel (230-400 mesh).

Synthesis of compounds 2-62

2,1′:4,6-di-*O*-isopropylidene sucrose 2

The synthesis of 2,1′:4,6-di-*O*-isopropylidene sucrose (diacetonide **2**) was accomplished according to Panda et. al. modified process of Falkenhagen *et. al.* [2-6]

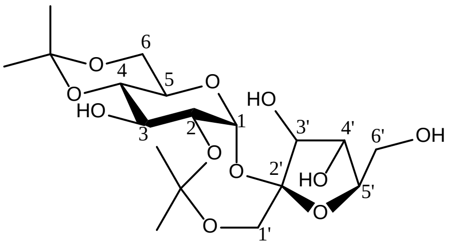

Diacetonide **2**

Drierite® (36.0 g) was added to dry DMF (700 mL) and the mixture was stirred under nitrogen atmosphere for an hour. Drierite® was removed by vacuum filtration and the solvent was collected. Finely grinded sucrose **1** (65.0 g, 190 mmol was then added to the filtered dry DMF and the mixture was stirred under nitrogen atmosphere for 30 minutes to ensure dissolution of sucrose. 2-Methoxypropene (86.0 mL, 898 mmol) and *p*-TsOH (82.0 mg, 0.431 mmol) were added to the mixture which was stirred for a week under inert atmosphere. Subsequently, the reaction mixture was quenched with the addition of NEt_3 (7.00 mL). DMF was evaporated under reduced pressure and the yellow gum obtained was then suspended in EtOAc (400 mL). After 10 minutes, the solution was filtered. The filtrate was then dried over anhydrous $MgSO_4$, evaporated and the crude product obtained was subjected to purification using column chromatography with pure EtOAc as the eluent. Diace-

tonide **2** was obtained as the major product in (52 g, 65% yield). Analytical data of diacetonide **2** corresponded to the literature values.[2-5]

Synthesis of 4′,6′-*O*-tetraisopropyldisilyl-2,1′:4,6-di-*O*-isopropylidene sucrose 3

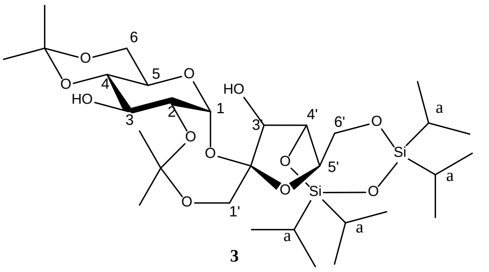

3

The synthesis of TIPDS-diacetonide **3** was accomplished accord- ing to the litera- ture procedure.[3,7] A mixture of diacetonide **2** (1.00 g, 2.37 mmol) and 1,3- dichloro-1,1,3,3-tetraisopropyldisiloxane (832 µL, 2.61 mmol) in pyridine (5.0 ml) was stirred at room temperature for 6 hours. Pyridine was re- moved under reduced pressure and the crude product was purified using sili- ca gel column chromatography using Hexane/EtOAc (3:2) as eluent. TIPDS- diacetonide **3** was obtained as white solid (1.18g, 75% yield). Analytical data for TIPDS- diacetonide **3** corresponded to the literature values[3].

General procedure for acylation of TIPDS-diacetonides 4, 5, 6, 7, 8, 9 and **10**

To a stirred solution of TIPDS-diacetonide **3** (300 mg, 0.452 mmol) and DMAP (11 mg, 0.09 mmol) in CH_2Cl_2 (100 mL) was added (substituted) cinnamic acids (1.81 mmol) and EDC (347 mg, 1.81 mmol) at room temperature. The reaction mixture was left to stir for a period of time. After completion (TLC), it was then washed with distilled water (3 x 30 ml), the organic phase was dried over anhydrous $MgSO_4$, filtered and evaporated under reduced pressure. The crude product was purified using silica gel column chromatography using Hexane/EtOAc (4:1) as eluent. This general procedure was used to prepare compounds **4, 5, 6, 7, 8, 9** and **10**, and also gave compounds **11, 12, 13, 14, 15, 16** and **17** side products.

3,3′-di-*O*-cinnamoyl-4′,6′-*O*-tetraisopropyldisilyl-2,1′:4,6-di-*O*-isopropylidene sucrose 4

Following the general procedure, the reaction using cinnamic acid (268 mg, 1.81 mmol) gave the required compound **4** (201 mg, 48%) and along with **11** (126 mg, 35%) as white solids.

Analytical data for compound 4: ^{1}H NMR (300 MHz, $CDCl_3$): TIPDS: δ 0.82-1.09 (m, 28H, 4 x H-a, 8 x $(CH_3)_2CSi$); isopropylidene rings: δ 1.32-1.42 (m, 12H, 2 x $(CH_3)_2C$)); sucrose unit: δ 3.50 (d, *J* = 12 Hz, 1H, H-1′), 3.65- 3.71 (m, 2H), 3.82-3.94 (m, 7H), 4.12- 4.16 (m, 1H), 4.65 (t, 1H, *J* = 9 Hz, H-4′), 5.18 (d, 1H, *J* = 8 Hz, H-3′), 5.26 (t, 1H, *J* = 16.5 Hz, H-3), 5.94 (d, 1H, *J* = 4 Hz, H-1); aromatic and alkenyl protons: δ 6.34 (d, 1H, *J* = 16 Hz, H-a′), 6.55 (d, 1H, *J* = 16 Hz, H-a$^{1′}$), 7.28-7.59 (m, 11H, H-b′, H-c′, H-c$^{1′}$, H-d′, H-d$^{1′}$, H-e′, H-e$^{1′}$, H-f′, H-f$^{1′}$ H-g′, H-g$^{1′}$), 7.77 (d, 1H, *J* = 16 Hz, H-b$^{1′}$); ^{13}C NMR (75.5 MHz, $CDCl_3$): δ 11.2, 12.6, 13.0, 13.5, 14.3, 17.1, 17.2, 17.3, 17.6, 17.7, 17.8, 19.2, 22.9, 13.9, 24.1, 25.6, 29.1, 29.2, 29.9, 30.6, 38.9, 62.5, 64.2, 66.2, 66.4, 68.4, 71.2, 71.9, 72.0, 78.9, 81.5, 91.3, 99.8, 101.3, 102.8, 117.3, 118.3, 128.2, 128.3, 128.8, 129.0, 129.1, 130.4, 130.6, 131.1, 132.6, 134.6, 144.9, 146.8, 166.1, 166.7, 168.0.

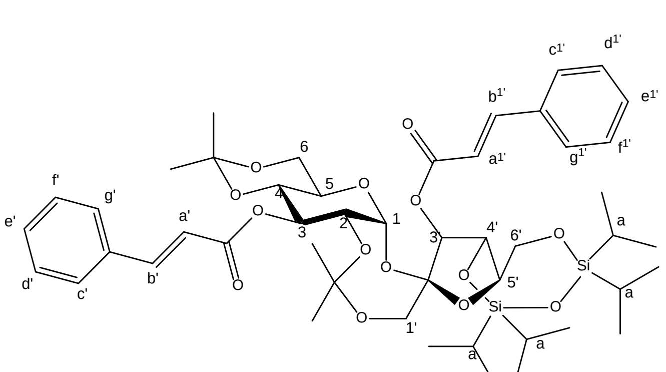

4

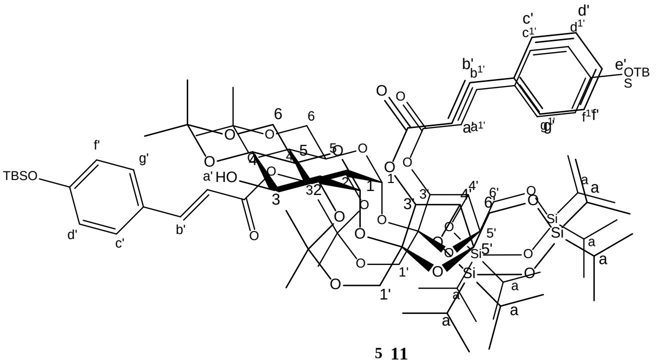

5 11

Analytical data for compound 11: The ^{1}H NMR data of com- pound **11** corresponded to our previously ob- tained data thus confirming the identity of the compound.[3]

Synthesis of 3,3′-di-*O*-(4-tert- butyldime- thylsiloxycinnamoyl)-4′,6′-*O*- tetrai- sopropyldisilyl-2,1′:4,6-di-*O*-isopropylidene sucrose 5

Following the general procedure, OTBS-coum acid (synthe- sized from the experimental procedure as shown in section 2.2) (503 mg, 1.81 mmol) was added to the solu- tion. After puri- fication, two products, compounds **5** and **12** were obtained as white solid compound in 37% (198 mg) and 49% (205 mg) yield

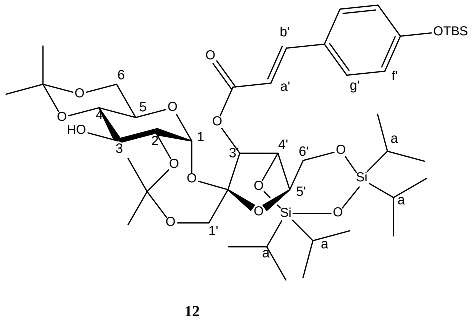

12

respectively.

Analytical data for compound 5: : ^{1}H NMR (300 MHz, $CDCl_3$): TBS protons: δ 0.19-0.22 (m, 12H), 1.13-1.21 (m, 18H); TIPDS: δ 0.89-1.09 (m, 28H, 4 x H-a, 8 x $(CH_3)_2CSi$); isopropylidene rings: δ 1.39-1.47 (m, 12H, 2 x $(CH_3)_2C$)); sucrose unit: δ 3.54 (d, *J* = 12 Hz, 1H, H-1′), 3.71-3.78 (m, 2H), 3.85-4.01 (m, 7H), 4.18-4.22 (m, 1H), 4.68 (t, 1H, *J* = 14 Hz, H-4′), 5.23 (d, 1H, *J* = 8 Hz, H-3′), 5.30 (t, 1H, *J* = 18.5 Hz, H-3), 5.97 (d, 1H, *J* = 4 Hz, H-1); aromatic and alkenyl protons:, 6.26 (d, 1H, *J* = 16 Hz, H-a′), 6.46 (d, 1H, *J* = 16 Hz, H-a$^{1'}$), 6.80-7.54 (m, 9H, H-b′, H-c′, H-c$^{1'}$, H-d′, H-d$^{1'}$, H-f′, H-f$^{1'}$ H-g′, H-g$^{1'}$), 7.78 (d, 1H, *J* = 16 Hz, H-b$^{1'}$); ^{13}C NMR (75.5 MHz, $CDCl_3$): δ -4.48, -4.18, 13.0, 13.5, 17.1, 17.2, 17.6, 17.7, 17.8, 18.0, 18.4, 19.2, 23.2, 24.1, 25.6, 25.8, 25.9, 29.1, 29.2, 30.6, 38.9, 62.5, 64.2, 66.3, 66.5, 71.1, 72.0, 81.5, 91.3, 99.8, 101.3, 102.8, 114.9, 116.0, 118.0, 120.7, 128.0, 129.8, 129.9, 130.5, 144.6, 145.1, 146.6, 158.0, 158.2, 166.4, 167.0.

Analytical data for compound 12: The ^{1}H NMR data of the compound was compared with literature values[3] and confirmed to be **12**.

Synthesis of 3,3′-di-*O*-(4-*tert*-butyldimethylsiloxy-3-methoxycinnamoyl)-4′,6′-*O*-tetraisopropyldisilyl-2,1′:4,6-di-*O*-isopropylidene sucrose 6

Following the general procedure, OTBS-feru acid (synthesized from the experimental procedure as shown in section 2.2) (557 mg, 1.81 mmol) was added to the solution. After purification, two products, compounds **6** and **13** were obtained as white solid compound in 30% (169 mg) and 35% (151 mg) yield respectively.

Analytical data for compound 6: ^{1}H NMR (300 MHz, $CDCl_3$): TBS protons: δ 0.10-0.13 (m, 12H), 1.18-1.27 (m, 18H); TIPDS: δ 0.86-1.05 (m, 28H, 4 x H-a, 8 x $(CH_3)_2CSi$); isopropylidene rings: δ 1.33-1.54 (m, 12H, 2 x $(CH_3)_2C$)); methoxy and sucrose unit: δ 3.50 (d, *J* = 12 Hz, 1H), 3.69- 3.74 (m, 2H), 3.77-3.84 (m, 6H, 3 x H-h′, 3 x H-h$^{1'}$), 3.94-3.99 (m, 5H), 4.15-4.18 (m, 1H), 4.65 (t, 1H, *J* = 13 Hz, H-4′), 5.20 (d, 1H, *J* = 8 Hz, H-3′), 5.30 (t, 1H, *J* = 19 Hz, H-3), 5.95 (d, 1H, *J* = 4 Hz, H-1); aromatic and alkenyl protons: δ 6.21 (d, 1H, *J* = 16 Hz, H-a′), 6.41 (d, 1H, J = 16 Hz, H-a$^{1'}$), 6.80-7.12 (m, 6H, H-c′, H-c$^{1'}$, H-d′, H-d$^{1'}$, H-g′, H-g$^{1'}$), 7.51 (d, 1H, *J* = 16 Hz, H-b′), 7.71 (d, 1H, *J* = 16 Hz, H-b$^{1'}$); ^{13}C NMR (75.5 MHz, $CDCl_3$): δ -4.42, -3.39, 12.6, 13.0, 13.5, 14.3, 17.1, 17.2, 17.6, 17.7, 17.8, 18.7, 19.1, 23.2, 24.2, 25.6, 25.9, 29.2, 30.6, 55.6, 55.7, 64.2, 66.3, 71.0, 72.0, 81.5, 91.3, 99.8, 101.3, 102.8, 111.0, 111.5, 115.0, 121.2, 121.3, 122.4, 123.1, 128.6, 145.0, 146.9, 147.7, 147.9, 151.4, 166.3, 167.0.

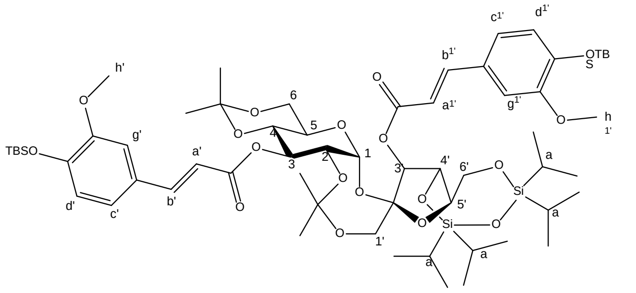

6

Analytical data for compound 13: The ^{1}H NMR data of the compound was compared with literature values[3] and confirmed to be **13**.

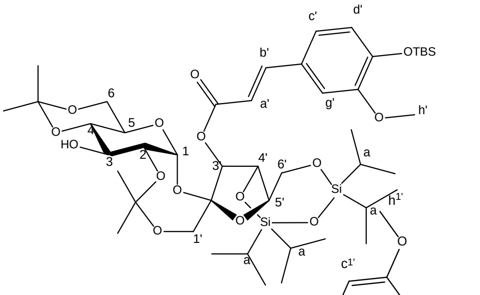

13

Synthesis of 3,3′-di-*O*-(4-*tert*-butyldimethylsiloxy-3,5-dimethoxycinnamoyl)-4′,6′-*O*-tetraisopropyldisilyl-2,1′:4,6-di-*O*-isopropylidene sucrose 7

Following the general procedure, OTBS-sinap acid (synthesized from the experimental procedure as shown in section 2.2) (612 mg, 1.81 mmol) was added to the solution. After purification, two products, compounds **7** and **14** were obtained as white solid compound in 22% (130 mg) and 27% (120 mg) yield respectively.

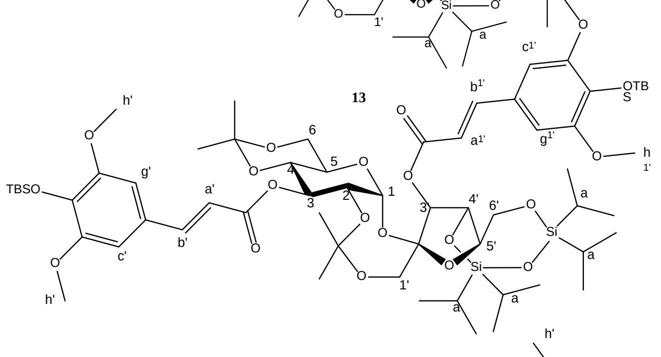

7

Analytical data for compound 7: ^{1}H NMR (300 MHz, $CDCl_3$): TBS protons: δ 0.09- 0.12 (m, 12H), 1.06, 1.10, 1.12 (m, 18H); TIPDS: δ 0.86- 1.02 (m, 28H, 4 x H-a, 8 x $(CH_3)_2CSi$); isopropylidene rings: δ 1.35-1.46 (m, 12H, 2 x $(CH_3)_2C$); methoxy and sucrose unit: δ 3.52 (d, 1H, *J* = 12.5 Hz), 3.68-3.71 (m, 2H), 3.74-3.83 (m, 12H, 6 x H-h′, 6 x H-h$^{1'}$), 3.85-3.97 (m,

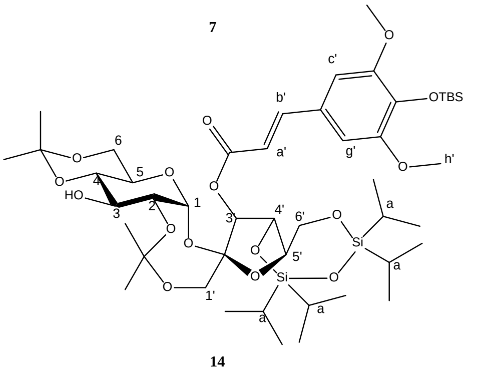

14

5H), 4.15-4.20 (m, 1H), 4.64 (t, 1H, *J* = 13 Hz, H-4′), 5.22 (d, 1H, *J* = 8 Hz, H-3′), 5.33 (t, 1H, *J* = 19 Hz, H-3), 5.97 (d, 1H, *J* = 4 Hz, H-1); aromatic and alkenyl protons: δ 6.24 (d, 1H, *J* = 16 Hz, H-a′), 6.42 (d, 1H, *J* = 16 Hz, H-a$^{1'}$), 6.68 (s, 2H, H-c′, H-g′), 6.82 (s, 2H, H-c$^{1'}$, H-g$^{1'}$), 7.50 (d, 1H, *J* = 16 Hz, H-b′), 7.70 (d, 1H, *J* = 16 Hz, H-b$^{1'}$); ^{13}C NMR (75.5 MHz, $CDCl_3$): δ -4.43, -4.40, 13.6, 17.1, 17.2, 17.3, 17.6, 17.7, 17.8, 18.9, 19.0, 19.1, 24.2, 25.6, 25.9, 26.0, 29.1, 29.2, 30.6, 38.9, 55.9, 56.0, 62.5, 64.1, 66.4, 71.0, 72.0, 81.6, 91.3, 99.8, 101.4, 102.8, 105.5, 106.1, 115.2, 116.0, 127.2, 127.3, 137.1, 137.2, 145.4, 147.2, 151.9, 152.0, 166.3, 166.9.

Analytical data for compound 14: The ^{1}H NMR data of the compound was compared with literature values[3] and confirmed to be **14**.

Synthesis of 3,3′-di-*O*-(3,4-di-*tert*-butyldimethylsiloxycinnamoyl)-4′,6′-*O*-tetraisopropyldisilyl-2,1′:4,6-di-*O*-isopropylidene sucrose 8

Following the general procedure, OTBS-caff acid (synthesized from the experimental procedure as shown in section 2.2) (738 mg, 1.81 mmol) was added to the solution. After purification, two products, compounds **8** and **15** were obtained as white solid compound in 34% (222 mg) and 27% (129 mg) yield respectively.

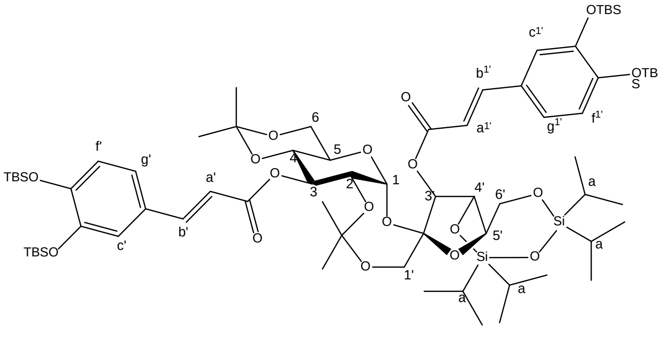

8

Analytical data for compound 8: ^{1}H NMR (300 MHz, $CDCl_3$): TBS: δ 0.18-0.19 (m, 24H), 0.87-1,12 (m, 36H); TIPDS: δ 0.87-1.12 (m, 28H, 4 x H-a, 8 x $(CH_3)_2CSi$); isopropylidene rings: δ 1.36-1.46 (m, 12H, 2 x $(CH_3)_2C$)); sucrose unit: δ 3.53 (d, 1H, *J* = 12.5 Hz), 3.70-3.73 (m, 2H), 3.85-4.01 (m, 5H), 4.18-4.22 (m, 1H), 4.65 (t, 1H, *J* = 14 Hz, H-4′), 5.16 (d, 1H, *J* = 8 Hz, H-3′), 5.29 (t, 1H, *J* = 19 Hz, H-3), 5.97 (d, 1H, J = 4 Hz, H-1); aromatic and alkenyl protons: δ 6.17 (d, 2H, *J* = 16 Hz, H-a′, H-a$^{1'}$), 6.77-6.83 (m, 2H, H-g′, H-g$^{1'}$), 6.96-7.10 (m, 4H, H-c′, H-c$^{1'}$, H-f′, H-f$^{1'}$), 7.47 (d, 1H, *J* = 16 Hz, H-b′), 7.68 (d, 1H, *J* = 16 Hz, H-b$^{1'}$); ^{13}C NMR (75.5 MHz, $CDCl_3$): δ-4.67, -4.11, -4.06, -4.02, 11.1, 12.3, 14.1, 16.9, 17.0, 17.4, 17.5, 17.8, 18.4, 18.5, 22.8, 24.0, 25.7, 25.9, 26.0, 31.3, 38.9, 62.6, 66.8, 68.5, 72.0, 81.1, 91.1, 99.8, 101.0, 103.1, 114.8, 117.7, 120.3, 121..1, 122.5, 128.1,145.2, 147.9, 149.6, 166.4, 167.1.

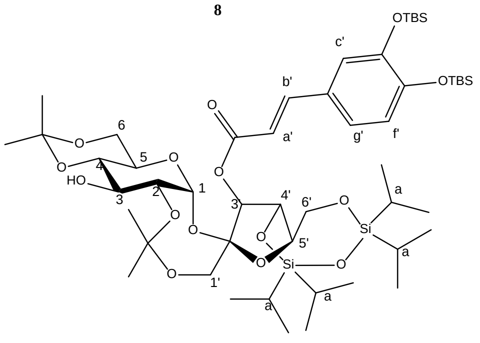

15

Analytical data for compound 15: The ^{1}H NMR data of the compound was compared with literature values[3] and confirmed to be **15**.

Synthesis of 3,3′-di-*O*-(3,4-dimethoxycinnamoyl)-4′,6′-*O*-tetraisopropyldisilyl-2,1′:4,6-di-*O*-isopropylidene sucrose 9

Following the general procedure, diOMe acid (synthesized from the experimental procedure as shown in section 2.2) (376 mg, 1.81 mmol) was added to the solution. After purification, two products, compounds **9** and **16** were obtained as white solid compound in 29% (137 mg) and 55% (212 mg) yield respectively.

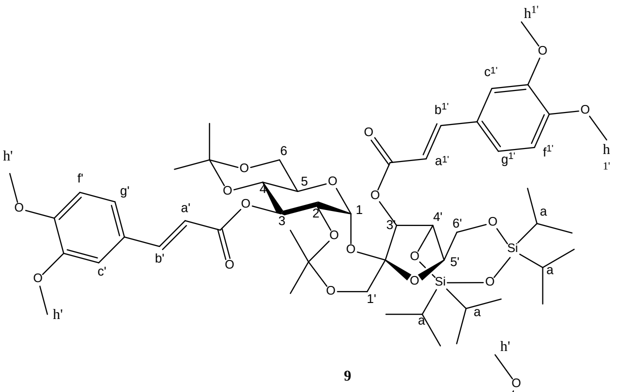

9

Analytical data for compound 9: ^{1}H NMR (300 MHz, $CDCl_3$): TIPDS: δ 0.88-1.12 (m, 28H, 4 x H-a, 8 x $(CH_3)_2CSi$); isopropylidene rings: δ 1.36-1.46 (m, 12H, 2 x $(CH_3)_2C$)); methoxy and sucrose unit: δ 3.88-3.90 (m, 12H, 6 x H-h′, 6 x H-h$^{1'}$), 3.51 (d, 1H, *J* = 12.5 Hz), 3.70-3.76 (m, 2H), 3.95-4.01 (m, 8H), 4.65 (t, 1H, *J* = 13.5 Hz, H-4′), 5.23 (d, 1H, *J* = 8 Hz, H-3′), 5.33 (t, 1H, *J* = 19 Hz, H-3), 5.97 (d, 1H, *J* = 4 Hz, H-1); aromatic and alkenyl protons: δ 6.25 (d, 1H, *J* = 16 Hz, H-a′), 6.45 (d, 1H, *J* = 16 Hz, H-a$^{1'}$), 6.80-6.87 (m, 2H, H-g′, H-g$^{1'}$), 7.01-7.06

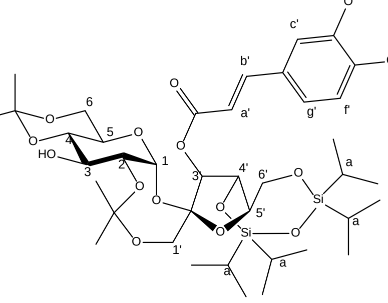

16

(m, 2H, H-c′, H-f′), 7.16-7.19 (m, 2H, H-c1′, H-f1′), 7.37 (d, 1H, *J* = 16 Hz, H-b′), 7.74 (d, 1H, *J* = 16 Hz, H-b1′); ^{13}C NMR (75.5 MHz CDCl$_3$): δ 12.0, 12.4 13.3, 16.1, 16.2, 16.6, 16.8, 16.9, 17.6, 18.2, 19.0, 25.4, 28.3, 55.2, 55.9, 56.0, 64.1, 65.3, 66.5, 70.1, 82.6, 87.2, 91.1, 92.8, 95.7, 98.9, 99.7, 100.4, 101.2, 101.8, 102.6, 108.6, 110.2, 111.0, 114.9, 122.3, 126.8, 137.6, 145.7, 148.4,149.2, 151.1, 166.1, 166.8.

Analytical data for compound 16: The ^{1}H NMR data of the compound was compared with literature values[3] and confirmed to be **16**.

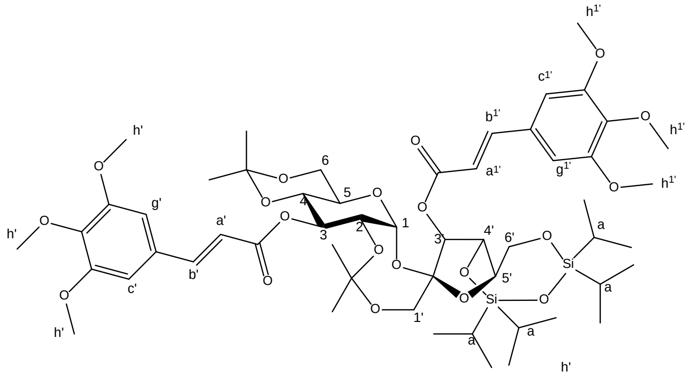

10

Synthesis of 3,3′-di-*O*-(3,4,5- trimethox-
ycinnamoyl)-4′,6′-*O*-tetraisopropyldisilyl- 2,1′:4,6-di-*O*-
isopropylidene sucrose 10

Following the general procedure, triOMe acid (synthesized
from the experimental procedure as shown in section 2.2) (430
mg, 1.81 mmol) was added to the solution. After purifica-
tion, two products, compounds **10** and **17** were obtained
as white solid compound in 34% (170 mg) and 36% (144
mg) yield respectively.

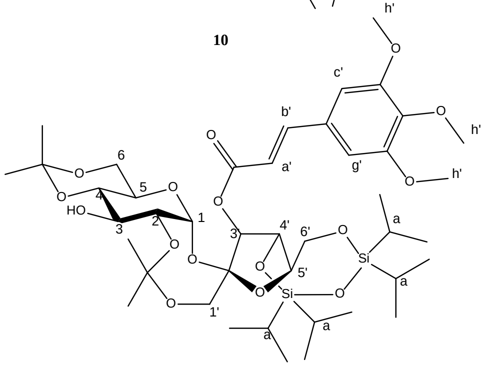

17

Analytical data for compound 10: ^{1}H NMR (300 MHz,
CDCl$_3$): TIPDS: δ 1.00-1.02 (m, 28H, 4 x H-a, 8 x (CH$_3$)$_2$CSi);
isopropylidene rings: δ 1.33-1.46 (m, 12H, 2 x (CH$_3$)$_2$C));
methoxy and sucrose unit: δ 3.80- 4.01 (m, 18H, 9 x H-h′, 9
x H-h1′), 3.52 (d, 1H, *J* = 12.5 Hz), 3.67-3.74 (m, 4H), 3.95-4.07
(m, 10H), 4.64 (t, 1H, *J* = 13.5 Hz, H-4′), 5.23 (d, 1H, *J* = 8 Hz, H-
3′), 5.32 (t, 1H, *J* = 18 Hz, H-3), 5.97 (d, 1H, *J* = 4 Hz, H-1); ar-
omatic and alkenyl protons: δ 6.27 (d, 1H, *J* = 16 Hz, H-a′),
6.47 (d, 1H, *J* = 16 Hz, H-a1′), 6.70 (s, 2H, H-c′, H-g′), 6.85 (s,
2H, H-c1′, H-g1′), 7.50 (d, 1H, *J* = 16 Hz, H-b′), 7.71 (d, 1H, *J* =
16 Hz, H-b1′); ^{13}C NMR (75.5 MHz, CDCl$_3$): δ 12.7, 13.0, 13.2,
13.5, 13.6, 17.1, 17.2, 17.3, 17.6, 17.7, 17.8, 19.2, 19.3, 25.6, 29.2,
31.1, 56.4, 61.2, 62.5, 64.2, 71.2, 72.0, 73.2, 91.4, 91.9, 99.9, 101.4, 102.8, 103.0, 105.5, 106.0, 106.3, 115.5, 117.3, 129.7, 130.1, 130.2, 147.7, 153.6, 153.7, 166.1, 167.2, 167.5 .

Analytical data for compound 17: The ^{1}H NMR data of the compound was compared with literature values[3] and confirmed to be **17**.

1.2.3.3 General procedure for the deprotection of TIPDS / TBS groups

The TIPDS / TBS protected compound was dissolved in pyridine (1.0 mL) at room temperature by stirring. Subsequently, NEt$_3$ and a solution of 1.56 M 3HF·NEt$_3$ in pyridine were added to the above solution and the reaction mixture was left to stir for 12 hours. Upon completion (TLC), pyridine was evaporated under reduced pressure, and the crude mixture was subjected to silica gel chromatographic purification using Hexane/EtOAc (2:3) as eluent. The general procedure was used to prepare 3,3′-cinn **18**, 3,3′-coum **19**, 3,3′-feru **20**, 3,3′-sinap **21**, 3,3′-caff **22**, 3,3′-diOMe **23** and 3,3′-triOMe **24** were obtained.

1.2.3.3.1 Synthesis of 3,3′-di-*O*-cinnamoyl-2,1′-di-*O*-isopropylidene sucrose, 3,3′-cinn 18

Following the general procedure, deprotection of compound **11** (80 mg, 0.09 mmol) using NEt$_3$ (24 μL, 0.17 mmol) and 1.56 M 3HF·NEt$_3$ (167 μL, 0.26 mmol) gave compound **18** as a white solid (41 mg, 67 % yield).

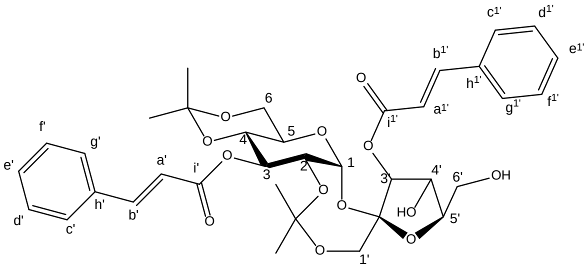

18

Analytical data for 3,3′-cinn 18: mp 57-59 °C; ^{1}H NMR (300 MHz,
CDCl$_3$): isopropylidene rings: δ 1.26-1.46 (m, 12H, 2 x (CH$_3$)$_2$C)); su-
crose unit: δ 3.52-3.56 (m, 1H, H-1′), 3.63-3.81 (m, 4H, H-2, H-4, H-6,
H-6′), 3.83, 3.85-3.98 (m, 3H, H-5, H-6, H-6′), 4.07-4.16 (m, 2H, H-1′, H-
5′), 4.90 (t, 1H, *J* = 14.5 Hz, H-4′), 4.96 (d, 1H, *J* = 8 Hz, H-3′), 5.47 (t, 1H,
J = 19 Hz, H-3), 6.25 (d, 1H, J = 4 Hz, H-1); aromatic and alkenyl

protons: δ 6.43 (d, 1H, *J* = 16 Hz, H-a′), 6.62 (d, 1H, J = 16 Hz, H-a$^{1'}$), 7.36-7.40 (m, 6H, H-d′, H-d$^{1'}$, H-e′, H-e$^{1'}$, H-f′, H-f$^{1'}$), 7.50-7.70 (m, 5H, H-b′, H-c′, H-c$^{1'}$, H-g′, H-g$^{1'}$) 7.89 (d, 1H, *J* = 16 Hz, H-b$^{1'}$); ^{13}C NMR (75.5 MHz, $CDCl_3$): δ 19.2, 24.2, 25.5, 29.2 (4 x $(CH_3)_2C$), 61.7 (C-6), 62.2 (C-5), 64.6 (C-6′), 66.6 (C-1′), 71.0 (C-3), 71.6 (C-4), 71.8 (C-2), 72.3 (C-4′), 81.5 (C-3′), 85.0 (C-5′), 91.5 (C-1), 100.0, 101.9 (2 x $(CH_3)_2C$), 104.0 (C-2′), 116.8, 118.2 (C-a′, C-a$^{1'}$), 128.3, 129.1 (C-c′, C-c$^{1'}$, C-g′, C-g$^{1'}$, C-d′, C-d$^{1'}$, C-f′, C-f$^{1'}$), 130.5, 131.0 (C-e′, C-e$^{1'}$), 134.2, 134.6 (C-h′, C-h$^{1'}$), 145.1, 147.8 (C-b′, C-b$^{1'}$), 166.1, 168.4 (C-i′, C-i$^{1'}$); HRMS (ESI positive mode): found *m/z* 705.2524 [M+Na]$^+$, calcd.705.2523 for $C_{36}H_{42}O_{13}Na$.

Synthesis of 3,3′-di-*O*-coumaroyl-2,1′-di-*O*-isopropylidene sucrose, 3,3′-coum 19

Following the general procedure, deprotection of compound **12** (100 mg, 0.08 mmol) using NEt_3 (24 μL, 0.17 mmol) and 1.56 M 3HF·NEt_3 (163 μL, 0.25 mmol) gave compound **19** as a white solid (33 mg, 58 % yield).

Analytical data for 3,3′-coum 19: mp 87-89 °C; ^1H NMR (300 MHz, $CDCl_3$): isopropylidene rings: δ 1.17-1.46 (m, 12H, 2 x $(CH_3)_2C$)); sucrose unit: δ 3.51-3.55 (m, 1H, H-1′), 3.73-3.86 (m, 7H, H-2, H-4, H-5, 2 x H-6′, 2 x H-6), 3.98- 4.04 (m, 2H, H-5′, H-1′), 4.61 (t, 1H, *J* = 14.5 Hz, H-4′), 5.12 (d, 1H, *J* = 8 Hz, H-3′), 5.36 (t, 1H, *J* = 18 Hz, H-3), 6.19 (d, 1H, *J* = 3.5 Hz, H-1); aromatic and alkenyl protons: δ 6.29 (d, 1H, *J* = 16 Hz, H-a′), 6.52 (d, 1H, *J* = 16 Hz, H-a$^{1'}$), 6.85-7.64 (m, 9H, H-b′, H-c′, H-c$^{1'}$, H-d′, H-d$^{1'}$, H-f′, H-f$^{1'}$ H-g′, H-g$^{1'}$), 7.79 (d, 1H, *J* = 16 Hz, H-b$^{1'}$); ^{13}C NMR (75.5 MHz, $CDCl_3$): δ 19.1, 24.1, 25.3 (4 x $(CH_3)_2C$), 62.4 (C-6), 63.0 (C-5), 64.7 (C-6′), 66.9 (C-1′), 71.1 (C-3), 71.2 (C-4), 71.8 (C-2), 72.4 (C-4′), 73.1, 79.4 (C-3′), 84.5 (C-5′), 91.8 (C-1), 100.1 (2 x $(CH_3)_2C$), 104.2 (C-2′), 114.3 (C-a′, C-a$^{1'}$), 115.2, 116.5 (C-d′, C-d$^{1'}$, C-f′, C-f$^{1'}$), 126.5 (C-h′, C-h$^{1'}$), 130.1, 131.2 (C-c′, C-c$^{1'}$, C-g′, C-g$^{1'}$), 145.3, 146.8 (C-b′, C-b$^{1'}$), 160.6, 160.8 (C-e′, C-e$^{1'}$), 166.3, 167.4 (C-i′, C-i$^{1'}$); HRMS (ESI positive mode): found *m/z* 737.2457 [M+Na]$^+$, calcd.737.2421 for $C_{36}H_{42}O_{15}Na$.

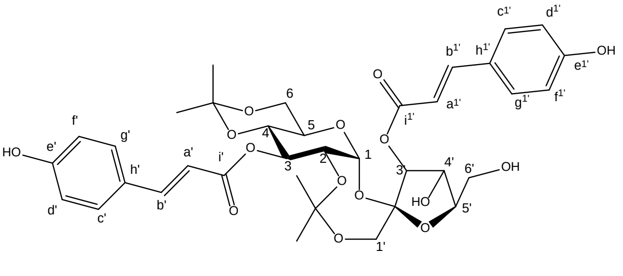

19

Synthesis of 3,3′-di-*O*-feruloyl-2,1′-di-*O*-isopropylidene sucrose, 3,3′-feru 20

Following the general procedure, deprotection of compound **13** (100 mg, 0.08 mmol) using NEt_3 (24 μL, 0.17 mmol) and 1.56 M 3HF·NEt_3 (163 μL, 0.25 mmol) gave compound **20** as a white solid (55 mg, 88% yield).

Analytical data for 3,3′-feru 20: mp 141-143°C; ^1H NMR (300 MHz, Acetone-d6): isopropylidene rings: δ 1.26-1.45 (m, 12H, 2 x $(CH_3)_2C$)); methoxy and sucrose unit: δ 3.91-3.96 (m, 6H, 3 x H-h′, 3 x H-h$^{1'}$), 3.51-3.55 (m, 1H, H-1′), 3.64-3.76 (m, 7H, H-4, 2 x H-6, 2 x H-6′, H-2, H-5), 4.07-4.16 (m, 2H, H-5′, H-1′), 4.79 (t, 1H, *J* = 14 Hz, H-4′), 4.93 (d, 1H, *J* = 7.5 Hz, H-3′), 5.50 (t, 1H, *J* = 19 Hz, H-3), 6.23 (d, 1H, *J* = 4 Hz, H-1); aromatic and alkenyl protons: δ 6.24 (d, 1H, *J* = 15 Hz, H-a′), 6.44 (d, 1H, *J* = 16 Hz, H-a$^{1'}$), 6.88-7.26 (m, 6H, H-c′, H-c$^{1'}$, H-d′, H-d$^{1'}$, H-g′, H-g$^{1'}$), 7.55 (d, 1H, *J* = 16 Hz, H-b′), 7.81 (d, 1H, *J* = 16 Hz, H-b$^{1'}$); ^{13}C NMR (75.5 MHz, $CDCl_3$): δ 19.2, 24.2, 25.6, 29.2 (4 x $(CH_3)_2C$), 56.2, 56.3 (2 x OCH_3), 61.7 (C-6), 62.2 (C-5), 64.6 (C-6′), 66.6 (C-1′), 70.8 (C-3), 71.6 (C-4′), 71.9 (C-2), 72.5 (C-4), 81.6 (C-3′), 85.1 (C-5′), 91.5 (C-1), 100.0, 101.9 (2 x $(CH_3)_2C$), 104.1 (C-2′), 109.6, 111.2 (C-g′, C-g$^{1'}$), 114.2, 114.9 (C-a′, C-a$^{1'}$), 115.0, 115.5 (C-d′, C-d$^{1'}$), 123.3, 123.6 (C-c′, C-c$^{1'}$), 127.1 (C-i′, C-i$^{1'}$), 145.2, 147.0 (C-b′, C-b$^{1'}$), 147.8 (C-f′, C-f$^{1'}$) , 148.3, 148.6 (C-e′, C-e$^{1'}$), 166.4, 168.7 (C-j′, C-j$^{1'}$); HRMS (ESI positive mode): found *m/z* 775.2818 [M+H]$^+$, calcd.775.2813 for $C_{38}H_{47}O_{17}$.

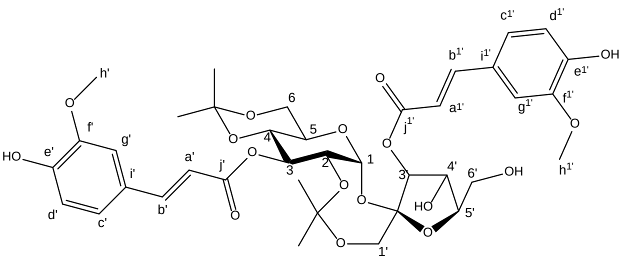

20

Synthesis of 3,3′-di-*O*-sinapoyl-2,1′-di-*O*-isopropylidene sucrose, 3.3′-sinap 21

Following the general procedure, deprotection of compound **14** (100 mg, 0.08 mmol) using NEt_3 (24 μL, 0.17 mmol) and 1.56 M 3HF·NEt_3 (163 μL, 0.25 mmol) gave compound **21** as a white solid (60 mg, 90 % yield).

Analytical data for 3,3′-sinap 21: mp 142-144 °C; ^{1}H NMR (300 MHz, Acetone-d6): isopropylidene rings: δ 1.26-1.45 (m, 12H, 2 x $(CH_3)_2C$)); methoxy and sucrose unit: δ 3.90-3.91 (m, 12H), 3.51-3.55 (m, 1H, H-1′) 3.64-3.79 (m, 7H, H-4, 2 x H-6, 2 x H-6′, H-2, H- 5), 4.07-4.16 (m, 2H, H-5′, H-1′), 4.78 (t, 1H, *J* = 14 Hz, H-4′), 4.93 (d, 1H, *J* = 7 Hz, H-3′), 5.53 (t, 1H, *J* = 18.5 Hz, H-3), 6.23 (d, 1H, *J* = 4 Hz, H-1); aro- matic and alkenyl protons: δ 6.27 (d, 1H, *J* = 15.5 Hz, H- a′), 6.42 (d, 1H, *J* = 16 Hz, H-a$^{1'}$), 6.74 (s, 2H H-c′, H-g′), 6.95 (s, 2H H- c$^{1'}$, H-g$^{1'}$), 7.52 (d, 1H, *J* = 16 Hz, H-b′), 7.80 (d, 1H, *J* = 16 Hz, H-b$^{1'}$); ^{13}C NMR (75.5 MHz, $CDCl_3$): δ19.2, 24.2, 25.6, 29.2 (4 x $(CH_3)_2C$), 56.6 (4 x OCH_3), 61.8 (C-6), 62.2 (C-5), 64.6 (C-6′), 66.5 (C-1′), 70.8 (C-3), 71.5 (C-4′), 71.9 (C-2), 72.7 (C-4), 81.8 (C-3′), 85.2 (C-5′), 91.5 (C-1), 100.0, 101.9 (2 x $(CH_3)_2C$), 104.2 (C-2′), 105.3, 106.1 (C-c′, C-c$^{1'}$, C-g′, C-g$^{1'}$), 114.6, 115.7 (C-a′, C-a$^{1'}$), 125.9 (C-i′, C-i$^{1'}$), 137.5, 137.7 (C-e′, C-e$^{1'}$), 145.5, 147.4, 147.5, 148.0 (C-b′, C-b$^{1'}$, C-d′, C-d$^{1'}$, C-f′, C-f$^{1'}$), 166.3, 168.5 (C-j′, C-j$^{1'}$); HRMS (ESI positive mode): found *m*/*z* 835.3001 [M+H]$^+$, calcd.835.3025 for $C_{40}H_{51}O_{19}$.

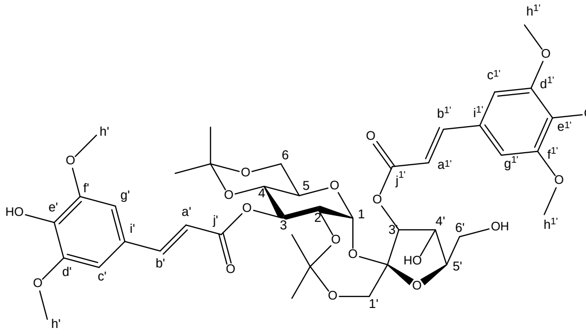

21

Synthesis of 3,3′-di-*O*-caffeoyl-2,1′-di-*O*-isopropylidene sucrose, 3.3′-caff 22

Following the general procedure, deprotection of compound **15** (100 mg, 0.07 mmol) using NEt_3 (19 μL, 0.14 mmol) and 1.56 M $3HF{\cdot}NEt_3$ (135 μL, 0.21 mmol) gave compound **22** as a yellow solid (28 mg, 54 % yield).

Analytical data for 3,3′-caff 22: mp 162-164 °C; ^{1}H NMR (300 MHz, Acetone-d6): isopropylidene rings: δ 1.22-1.47 (m, 12H, 2 x $(CH_3)_2C$)); sucrose unit: δ 3.51-3.55 (m, 1H, H-1′), 3.69-3.86 (m, 7H, H-4, 2 x H-6, 2 x H-6′, H-2, H-5), 4.00-4.09 (m, 2H, H- 5′, H-1′), 4.66 (t, 1H, *J* = 15 Hz, H-4′), 5.13 (d, 1H, *J* = 8 Hz, H-3′), 5.39 (t, 1H, *J* = 18 Hz, H-3), 6.21 (d, 1H, *J* = 4 Hz, H-1); aromatic and alkenyl protons: δ 6.25 (d, 1H, *J* = 16 Hz, H-a′), 6.43 (d, 1H, *J* = 16 Hz, H-a$^{1'}$), 6.83-6.90 (m, 2H, H-f′, H-f$^{1'}$), 7.03 (d, 1H, *J* = 8 Hz, H-g′), 7.15-7.16 (m, 2H, H-g$^{1'}$, H-c′), 7.32 (d, 1H, *J* = 2 Hz, H-c$^{1'}$), 7.52 (d, 1H, *J* = 16 Hz, H- b′), 7.73 (d, 1H, *J* = 16 Hz, H-b$^{1'}$); ^{13}C NMR (75.5 MHz, $CDCl_3$): δ19.1, 19.4, 24.4, 25.7 (4 x $(CH_3)_2C$), 62.7 (C-6), 63.2 (C-5), 65.1 (C-6′), 67.1 (C-1′), 71.4 (C- 3), 72.4 (C-4′), 72.7 (C-2), 73.1 (C-4), 79.8 (C-3′), 84.9 (C-5′), 92.1 (C- 1), 100.4 (2 x $(CH_3)_2C$), 104.6 (C-2′), 114.7 (C-a′, C-a$^{1'}$), 115.6, 116.5 (C-c′, C-c$^{1'}$, C-f′, C-f$^{1'}$), 122.3, 123.5 (C-g′, C-g$^{1'}$), 127.6, 127.7 (C-h′, C-h$^{1'}$), 146.0, 146.5, 147.5, 149.3 (C-b′, C-b$^{1'}$, C-d′, C-d$^{1'}$, C-e′, C-e$^{1'}$), 166.6, 167.7 (C-i′, C-i$^{1'}$); HRMS (ESI positive mode): found *m*/*z* 769.2314 [M+Na]$^+$, calcd.769.2320 for $C_{36}H_{42}O_{17}Na$.

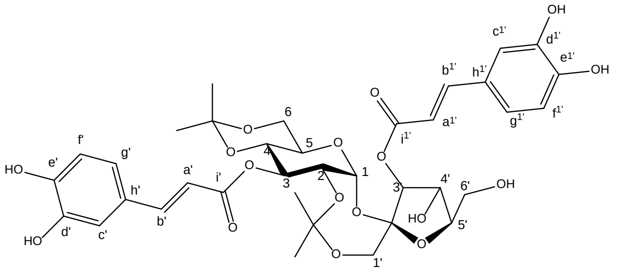

22

Synthesis of 3,3′-di-*O*-(3,4-dimethoxycinnamoyl)-2,1′-di-*O*-isopropylidene sucrose, 3,3′-diOMe 23

Following the general procedure, deprotection of compound **16** (100 mg, 0.10 mmol) using NEt_3 (28 μL, 0.20 mmol) and 1.56 M $3HF{\cdot}NEt_3$ (192 μL, 0.30 mmol) gave compound **23** as a white solid (52 mg, 65 % yield).

Analytical data for 3,3′-diOMe 23: mp 117-119 °C; ^{1}H NMR (300 MHz, , $CDCl_3$): isopropylidene rings: δ 1.25-1.45 (m, 12H, 2 x $(CH_3)_2C$)); methoxy and sucrose unit: δ 3.89-3.90 (m, 12H, 6 x H-h′, 6 x H-h$^{1'}$), 3.51-3.54 (m, 1H, H-1′), 3.63-3.67 (m, 3H, H-4, H-6, H-6′), 3.73- 3.78 (m, 1H, H-2), 3.83-3.86 (m, 1H, H-6′), 3.93-3.95 (m, 2H, H-5, H-6), 4.10-4.13 (m, 1H, H-5′), 4.14-4.15 (m, 1H, H-1′), 4.80 (t, 1H, *J* = 14 Hz, H-4′), 4.94 (d, 1H, *J* = 7.5 Hz, H-3′), 5.49-5.55 (t, 1H, *J* = 19 Hz, H-3), 6.24 (d, 1H, *J* = 4 Hz, H-1); aromatic and alkenyl pro- tons: δ 6.27 (d, 1H, *J* = 16 Hz, H-a′), 6.46 (d, 1H, *J* = 16 Hz, H-a$^{1'}$), 6.83- 6.87 (m, 2H, H- g′, H-g$^{1'}$), 7.02-7.06 (m, 2H, H-c′, H-f′), 7.26-7.27 (m, 2H, H-c$^{1'}$, H-f$^{1'}$), 7.56 (d, 1H, *J* = 16 Hz, H-b′) 7.82 (d, 1H, *J* = 16 Hz, H-b$^{1'}$); ^{13}C NMR (75.5 MHz, , $CDCl_3$): δ 19.2, 24.2, 25.5, 29.2 (4 x $(CH_3)_2C$), 56.1, 56.2 (4 x OCH_3), 61.7 (C-6), 62.2 (C-5), 64.6 (C-6′), 66.5 (C-1′), 70.8 (C-3), 71.6 (C-4′), 71.9 (C-2), 72.4 (C-4), 81.3 (C-3′), 85.0 (C-5′), 91.5 (C-1), 100.0, 101.9 (2 x $(CH_3)_2C$), 104.0 (C-2′), 109.7, 111.2, 111.3 (C-c′, C-c$^{1'}$,

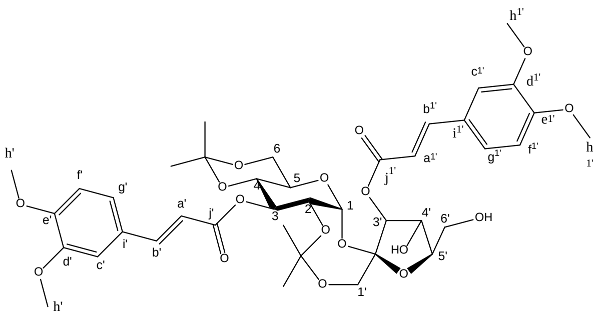

23

C-f′, C-f$^{1'}$), 114.5 , 115.7 (C-a′, C-a$^{1'}$), 122.9, 123.0 (C-g′, C-g$^{1'}$), 127.4, 127.5 (C-i′, C-i$^{1'}$), 145.1, 147.6 (C-b′, C-b$^{1'}$), 149.3, 149.4 (C-e′, C-e$^{1'}$), 151.4, 151.6 (C-d′, C-d$^{1'}$), 166.4, 168.6 (C-j′, C-j$^{1'}$); HRMS (ESI positive mode): found *m/z* 803.3152 $[M+H]^+$, calcd.803.3126 for $C_{40}H_{51}O_{17}$.

Synthesis of 3,3′-di-*O*-(3,4,5-trimethoxycinnamoyl)-2,1′-di-*O*-isopropylidene sucrose, 3,3′-triOMe 24

Following the general procedure, deprotection of compound **17** (100 mg, 0.09 mmol) using NEt_3 (25 µL, 0.18 mmol) and 1.56 M 3HF·NEt_3 (173 µL, 0.27 mmol) gave compound **24** as a white solid (75 mg, 97 % yield).

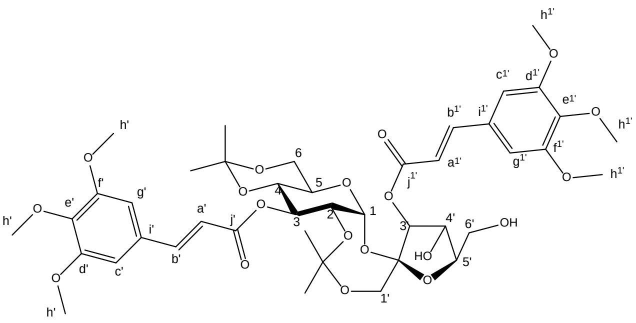

24

Analytical data for 3,3′-triOMe 24: mp 116-118 °C; ^{1}H NMR (300 MHz, $CDCl_3$): isopropylidene rings: δ 1.25-1.60 (m, 12H, 2 x $(CH_3)_2C$)); methoxy and sucrose unit: δ 3.86-3.87 (m, 18H, 9 x H-h′, 9 x H-h$^{1'}$), 3.51-3.55 (m, 1H, H-1′), 3.59-3.79 (m, 7H, H-4, 2 x H-6, 2 x H-6′, H-2, H-5), 4.10-4.16 (m, 2H, H-5′, H-1′), 4.79 (t, 1H, *J* = 14 Hz, H-4′), 4.95 (d, 1H, *J* = 7 Hz, H-3′), 5.52 (t, 1H, *J* = 19 Hz, H-3), 6.25 (d, 1H, *J* = 4 Hz, H-1); aromatic and alkenyl protons: δ 6.31 (d, 1H, *J* = 16 Hz, H-a′), 6.48 (d, 1H, *J* = 16 Hz, H-a$^{1'}$), 6.72 (s, 2H, H-c′, H-g′), 6.92 (s, 2H, H-c$^{1'}$, H-g$^{1'}$), 7.51 (d, 1H, *J* = 16 Hz, H-b′) 7.80 (d, 1H, *J* = 16 Hz, H-b$^{1'}$); ^{13}C NMR (75.5 MHz, , $CDCl_3$): δ 19.1, 24.1, 25.5, 29.1, 29.9 (4 x $(CH_3)_2C$), 56.4, 61.1, 61.2 (6 x OCH_3), 61.7 (C-6), 62.1 (C-5), 64.6 (C-6′), 66.5 (C-1′), 70.8 (C-3), 71.5 (C-4′), 71.8 (C-2), 72.4 (C-4), 81.2 (C-3′), 84.9 (C-5′), 91.4 (C-1), 100.0, 101.9 (2 x $(CH_3)_2C$), 104.0 (C-2′), 105.5, 106.2 (C-c′, C-c$^{1'}$, C-g′, C-g$^{1'}$), 116.1, 117.1 (C-a′, C-a$^{1'}$), 129.8, 129.9 (C-i′, C-i$^{1'}$), 140.4, 140.6 (C-e′, C-e$^{1'}$), 145.2, 147.5 (C-b′, C-b$^{1'}$), 153.5, 153.6 (C-d′, C-d$^{1'}$, C-f′, C-f$^{1'}$), 166.1, 168.1 (C-j′, C-j$^{1'}$); HRMS (ESI positive mode): found *m/z* 885.3123 $[M+Na]^+$, calcd. 885.3157 for $C_{42}H_{54}O_{19}Na$.

Synthesis of 6′-*O*-tert-butyldimethylsiloxy-2,1′:4,6-di-*O*-isopropylidene sucrose, 6′-*O*-TBS 25

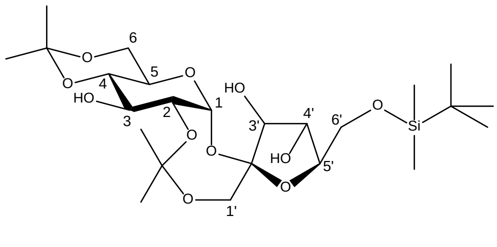

25

The synthesis of 6′-*O*-TBS **25** was accomplished according to the literature procedure.[3] To a solution of 2,1′:4,6-di-*O*-isopropylidene sucrose **2** (1.00 g, 2.37 mmol) and DMAP (28.9 mg, 0.237 mmol) in CH_2Cl_2 (50.0 ml), NEt_3 (793 µL, 5.68 mmol) and TBSCl (428 mg, 2.84 mmol) were added at room temperature. The reaction mixture was then left to stir for 12 hours. Upon completion (TLC), the reaction mixture was washed with 10% NH_4Cl solution (2 x 15 mL), the organic layer collected, dried over anhydrous $MgSO_4$ and CH_2Cl_2 was removed under reduced pressure. The crude mixture was purified using silica gel column chromatography using EtOAc/hexane (1:1) as eluent. 6′-*O*-TBS **25** (1.21 g, 95%) was obtained as white solid. The ^{1}H NMR data of the product confirmed with the literature values.[3]

Synthesis of 3′-*O*-carboxybenzyl-6′-*O*-tert-butyldimethylsiloxy-2,1′-di-*O*-isopropylidene sucrose, 3′-*O*-Cbz 26

The synthesis of 3′-*O*-Cbz **26** was accomplished according to the literature procedure.[3] To a stirred solution of 6′-*O*-TBS **25** (500 mg, 0.932 mmol) and DMAP (11.4 mg, 0.0932 mmol) in CH_2Cl_2 (20.0 ml) was added TMEDA (420 µL, 2.80 mmol) and CbzCl (199 µL, 1.40 mmol) at room temperature. The reaction was left to stir for 12 hours. Upon completion (TLC), the reaction was washed water (2 x 10 mL), the organic layer dried over anhydrous $MgSO_4$, CH_2Cl_2 was removed and the crude mixture was purified using column chromatog-raphy with a gradient of EtOAc/hexane as eluent (starting with Hex/EtOAc (8:1), then Hexane/EtOAc (6:1) and Hexane/EtOAc (4:1)). 3′-*O*-Cbz **26** was obtained as white solid (469 mg, 75% yield). The ^{1}H NMR data of the prod-uct confirmed with the literature values.[3]

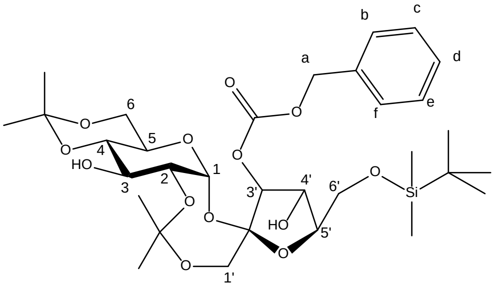

26

1.2.4.3 General procedure for acylation of 3′-*O*-Cbz 26

To a stirred solution of 3′-*O*-Cbz **26** (300 mg, 0.448 mmol) and DMAP (11 mg, 0.09 mmol) in CH_2Cl_2 (20 mL) was added the (substituted) cinnamic acid (1.79 mmol) and EDC (344 mg, 1.79 mmol) at room temperature. The reaction mixture was left to stir for a week, washed with distilled water (2 x 10 mL), the organic phase dried over anhydrous $MgSO_4$ and the solvent evaporated under reduced pressure. The crude product was purified using silica gel column chromatography using Hexane/EtOAc (8:1) as eluent. The general procedure was used to prepare compounds **27, 28, 29, 30, 31** and **32** and also gave compounds **33, 34, 35, 36, 37** and **38** as by products.

Synthesis of 3,4′-di-*O*-(4-*tert*-butyldimethylsiloxycinnamoyl)- 3′-*O*-carboxybenzyl-6′-*O*-*tert*-butyldimethylsiloxy-2,1′-di-*O*-isopropylidene sucrose 27

Following the general procedure, OTBS-coumaric acid (498 mg, 1.79 mmol) gave compound **27** (192 mg, 36% yield) and by product **33** (258 g, 62% yield) as white solids.

Analytical data for compound 27: ^{1}H NMR (300 MHz, $CDCl_3$): TBS groups: δ 0.02 (s, 6H), 0.83 (s, 9H); TBS on OTBS couma-royl group: δ 0.18-0.19 (m, 12H), 0.95 (m, 18H); isopropy-lidene rings: δ 1.22-1.44 (m, 12H, 2 x $(CH_3)_2C$)); Cbz and su-crose unit: δ 3.49 (d, 1H, *J* = 12 Hz), 3.71-3.90 (m, 7H), 4.15-4.21 (m, 2H), 4.89 (d, 1H, *J* = 4 Hz, H-3′), 5.26-5.41 (m, 3H, H-3, 2 x H-a), 5.52 (t, 1H, *J* = 7 Hz, H-4′), 6.06 (d, 1H, *J* = 3.5 Hz, H-1); Cbz, OTBS-coumaroyl aromatic and alkenyl protons: δ 6.26 (dd, 2H, *J* = 34 Hz, H-a′, Ha1′), 6.81 (d, 4H, *J* = 9 Hz, H-d′, H-f′, Hd1′, H-f1′), 7.23- 7.46 (m, 9H, H-c′, H-g′, Hc1′, H-g1′, H-b, H-c, H-d, H-e, H-f), 7.57-7.64 (m, 2H, H-b′, H-b1′); ^{13}C NMR (75.5 MHz, $CDCl_3$): δ -5.16, -5.12, -4.17, 18.5, 19.3, 24.1, 25.8, 26.1, 29.2, 62.6, 63.7, 64.3, 70.8, 70.9, 71.9, 72.2, 81.7, 83.4, 92.0, 99.9, 101.5, 105.0, 115.0, 116.1, 120.7, 120.8, 127.7, 128.1, 128.6, 128.8, 129.9, 130.1, 135.4, 144.7, 145.9, 154.7, 158.0, 158.3, 166.0, 166.4.

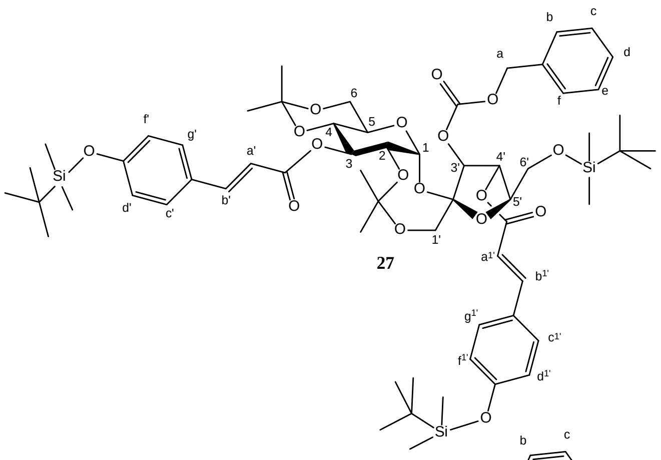

27

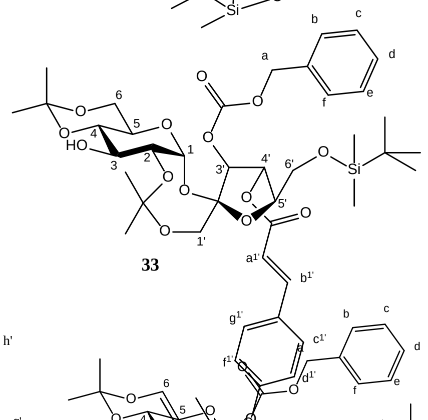

33

Analytical data for compound 33: The ^{1}H NMR data of compound **33** confirmed to the literature values.[3]

Synthesis of 3,4′-di-*O*-(4-*tert*-butyldimethylsiloxy-3-methoxycinnamoyl) -3′-*O*-carboxybenzyl-6′-*O*-*tert*-butyldimethylsiloxy-2,1′-di-*O*-isopropylidene sucrose 28

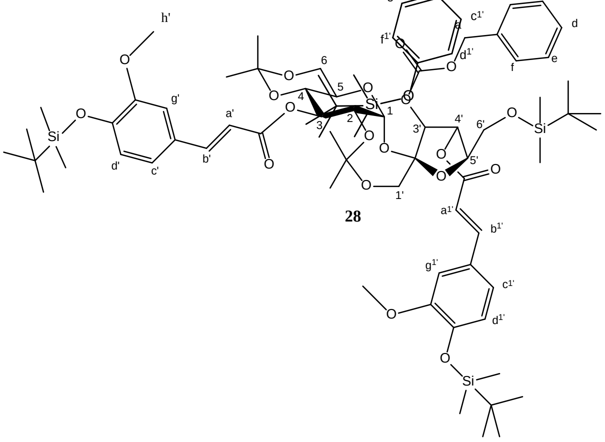

28

Following the general procedure, OTBS-feru acid (552 mg, 1.79 mmol) gave compound **28** (140 mg, 25% yield) and by-product **34** (151 mg, 35% yield) as white solids.

Analytical data for compound 28: ^{1}H NMR (300 MHz, $CDCl_3$): TBS group: δ 0.03 (s, 6H, $(CH_3)_2Si$), 0.84 (s, 9H, $(CH_3)_3CSi$); TBS on OTBS-feruoyl group: 0.14-0.15 (m, 12H, 2 x $(CH_3)_2Si$), 0.97 (m, 18H, 2 x $(CH_3)_3CSi$); isopropylidene rings: δ 1.23-1.55 (m, 12H, 2 x $(CH_3)_2C$)); Cbz, methoxy and sucrose unit: δ 3.81-3.83 (m, 6H, 3 x H-h′, 3 x H-h1′), 3.50 (d, 1H, *J* = 12 Hz), 3.72-3.79 (m, 4H), 3.88-3.91 (m, 4H), 4.15-4.21 (m, 2H), 4.89 (d, 1H, *J* = 4 Hz, H-3′), 5.28-5.42 (m, 3H, H-3, 2 x H-a), 5.53 (t, 1H, *J* = 7 Hz, H-4′), 6.07 (d, 1H, *J* = 3.5 Hz, H-1); Cbz, OTBS-feruloyl aromatic and alkenyl protons: δ 6.27 (dd, 2H, *J* = 33 Hz, H-a′, H-a1′), 6.82 (d, 2H, *J* = 9 Hz, H-c′, H-c1′), 6.98-7.02 (m, 4H, H-d′, H-g′, H-d1′,H-g1′), 7.30-7.47 (m, 5H, H-b, H-c, H-d, H-e, H-f), 7.57 (d, 1H, *J* = 6 Hz, H-b′), 7.62 (d, 1H, *J* = 6.5 Hz, H-b1′); ^{13}C NMR (75.5 MHz, $CDCl_3$): δ -5.37, -5.33, -4.60, -4.59, 18.3, 18.5, 19.0, 23.9, 25.7, 25.9, 55.5, 62.4, 63.5, 64.1, 66.7,70.6, 70.7, 71.7, 72.0, 81.5, 83.2, 91.8, 99.7, 101.3,

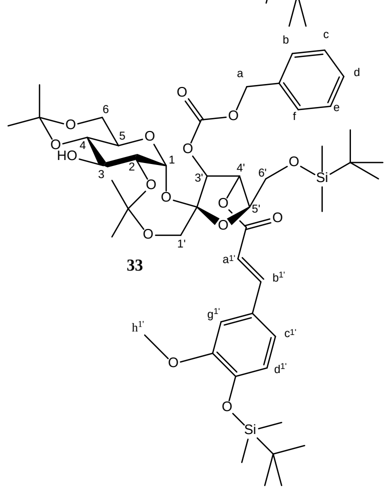

33

104.8, 110.8, 110.9, 114.8, 115.9, 121.1, 122.2, 122.5, 128.1, 128.4, 128.6, 135.2, 144.8, 146.0, 147.5, 147.8, 151.2, 154.5, 165.7, 166.1.

Analytical data for compound 34: The ^{1}H NMR data of the compound was compared with literature values[3] and confirmed to be **34**.

Synthesis of 3,4′-di-*O*-(4-*tert*-butyldimethylsiloxy-3,5-dimethoxycinnamoyl) -3′-*O*-carboxybenzyl-6′-*O*-*tert*-butyldimethylsiloxy-2,1′-di-*O*-isopropylidene sucrose 29

Following the general procedure, OTBS-sinapic acid (605 mg, 1.79 mmol) gave compound **29** (182 mg, 31% yield) and by-product **35** (182 mg, 41% yield) as white solids.

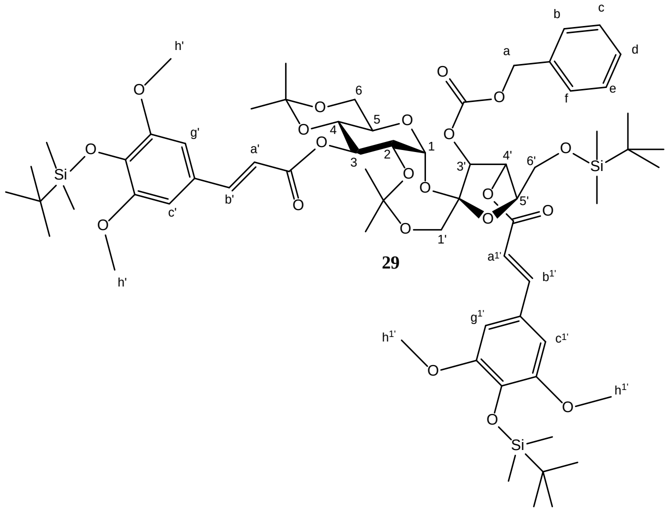

29

Analytical data for compound 29: ^{1}H NMR (300 MHz, CDCl$_3$): TBS group: δ 0.03 (s, 6H, (CH$_3$)$_2$Si), 0.85 (s, 9H, (CH$_3$)$_3$CSi); TBS on OTBS-sinapoyl groups: δ 0.11-0.12 (m, 12H, 2 x (CH$_3$)$_2$Si), 0.98 (m, 18H, 2 x (CH$_3$)$_3$CSi); iso-propylidene rings: δ 1.24-1.45 (m, 12H, 2 x (CH$_3$)$_2$C)); Cbz, methoxy and sucrose unit: δ 3.51 (d, 1H, *J* = 12 Hz), 3.72-3.78 (m, 3H), 3.80 (m, 12H, 6 x H-h′, 6 xh$^{1'}$), 3.89-3.92 (m, 4H), 4.15, 4.20, 4.22 (m, 2H), 4.90 (d, 1H, *J* = 4 Hz, H-3′), 5.29-5.42 (m, 3H, H-3, 2 x H-a), 5.53 (t, 1H, *J* = 7.5 Hz, H-4′), 6.07 (d, 1H, *J* = 4 Hz, H-1); Cbz, OTBS-sinapoyl aromatic and alkenyl protons: δ 6.27 (dd, 2H, *J* = 32.5 Hz, H-a′, H-a$^{1'}$), 6.71 (d, 4H, *J* = 8 Hz, H-c′, H-g′, H-a$^{1'}$, H- g$^{1'}$), 7.30-7.48 (m, 5H, H-b, H-c, H-d, H-e, H-f), 7.55 (d, 1H, *J* = 6 Hz, H-b′), 7.61 (d, 1H, *J* = 6 Hz, H-b$^{1'}$); ^{13}C NMR (75.5 MHz, CDCl$_3$): δ -5.18, -5.14, -4.43, -4.40, 18.5, 18.9, 19.2, 24.1, 25.8, 25.9, 26.1, 29.2, 55.9, 62.6, 63.7, 64.3, 66.8, 70.8, 70.9, 71.9, 72.1, 81.7, 83.4, 92.0, 99.8, 101.5, 105.0, 105.5, 105.6, 115.1, 116.2, 127.0, 127.3, 128.6, 128.8, 135.3, 137.1, 137.3, 145.4, 146.6, 152.0, 154.7, 165.8, 166.2.

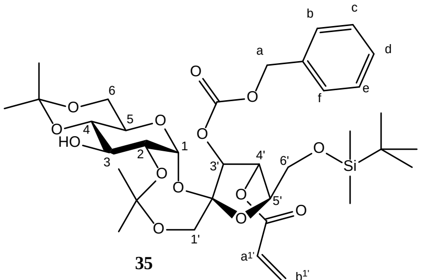

35

Analytical data for compound 35: The ^{1}H NMR data of the compound was compared with literature values[3] and con-firmed to be **35**.

Synthesis of 3,4′-di-*O*-(3,4-di-*tert*-butyldimethylsiloxycinnamoyl) -3′-*O*-carboxybenzyl-6′-*O*-*tert*-butyldimethylsiloxy-2,1′-di-*O*-isopropylidene sucrose 30

Following the general procedure, OTBS-caffeic acid gave compound **30** (91 mg, 14% yield) and by-product **36** (176 mg, 37% yield) as white solids.

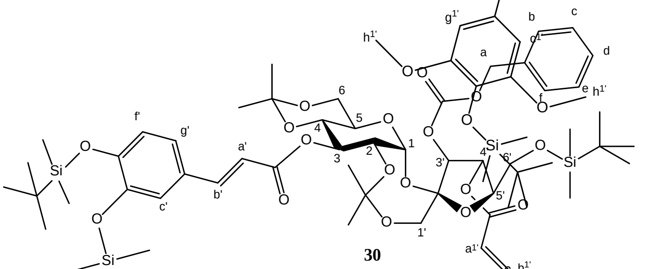

30

Analytical data for compound 30: ^{1}H NMR (300 MHz, CDCl$_3$): TBS group: δ 0.18 (s, 6H, (CH$_3$)$_2$Si), 0.83 (s, 9H, (CH$_3$)$_3$CSi); TBS on OTBS-caffeoyl groups: δ 0.18-0.19 (m, 24H, 4 x (CH$_3$)$_2$Si), 0.95-0.96 (m, 36H, 4 x (CH$_3$)$_3$CSi); isopropy-lidene rings: δ 1.23-1.44 (m, 12H, 2 x ((CH$_3$)$_2$C); Cbz and sucrose unit: δ 3.51 (d, 1H, *J* = 12 Hz), 3.65-3.92 (m, 7H), 4.15, 4.20, 4.22 (m, 2H), 4.88 (d, 1H, *J* = 4 Hz, H-3′), 5.27- 5.41 (m, 3H, H-3, 2 x H-a), 5.51 (t, 1H, *J* = 7 Hz, H-4′), 6.06 (d, 1H, *J* = 4 Hz, H-1); Cbz and aro-matic and alkenyl protons: δ 6.19 (dd, 2H, *J* = 34 Hz, H-a′, H-a$^{1'}$), 6.77-6.80 (m, 2H, H-f′, H-f$^{1'}$), 6.96-6.99 (m, 4H, H-c′, H-c$^{1'}$, H-g′, H-g$^{1'}$), 7.29-7.46 (m, 5H, H-b, H-c, H-d, H-e, H-f), 7.51 (d, 1H, *J* = 6 Hz, H-b′), 7.56 (d, 1H, *J* = 6 Hz, H-b$^{1'}$); ^{13}C NMR (75.5 MHz, CDCl$_3$): δ -5.15, -5.12, -3.88, -3.83, 18.5, 18.7, 19.3, 24.1, 26.1, 63.7, 64.3, 66.9, 70.8, 71.0, 71.9, 72.1, 81.7, 83.4, 92.0, 99.9, 101.6, 105.0,

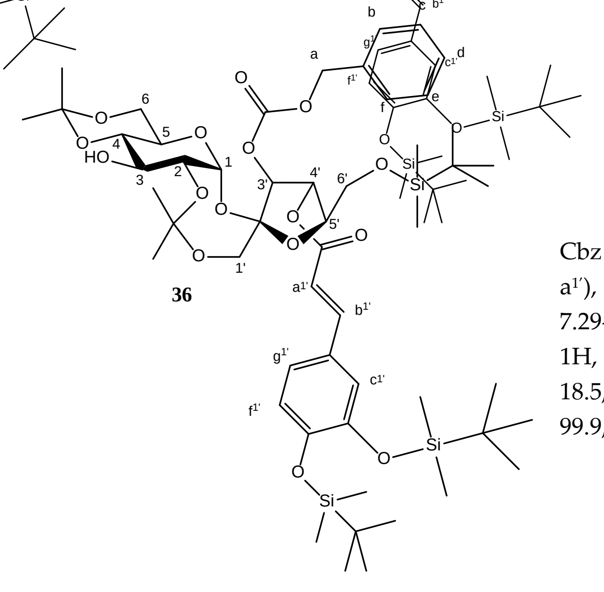

36

114.9, 116.0, 120.7, 121.4, 122.2, 122.7, 128.6, 128.8, 135.4, 144.9, 146.1, 147.4, 149.6, 149.9, 154.7, 166.0, 166.4.

Analytical data for compound 36: The ^{1}H NMR data of the compound was compared with literature values[3] and confirmed to be **36**.

Synthesis of 3,4′-di-*O*-(3,4-dimethoxycinnamoyl) -3′-*O*-carboxybenzyl-6′-*O*-*tert*-butyldimethylsiloxy-2,1′-di-*O*-isopropylidene sucrose 31

Following the general procedure, diOMe acid (373 mg, 1.79 mmol) gave compound **31** (141 mg, 30% yield) and by-product **37** (150 mg, 39% yield) as white solids.

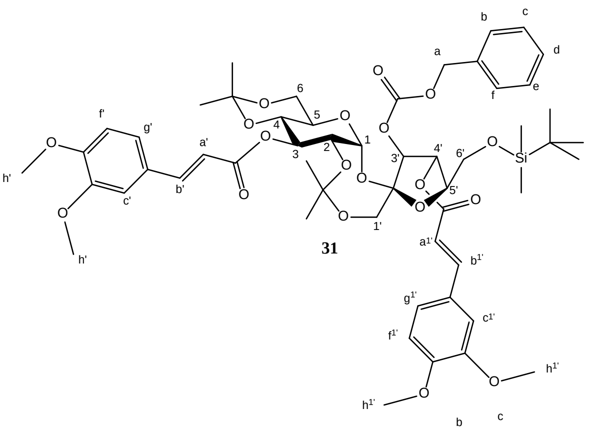

31

Analytical data for compound 31: ^{1}H NMR (300 MHz, [illegible] $CDCl_3$): TBS group: δ 0.03 (s, 6H, $(CH_3)_2Si$), 0.84 (s, 9H, $(CH_3)_3CSi$); [illegible] isopropylidene rings: δ 1.23-1.45 (m, 12H, 2 x $(CH_3)_2C$)); Cbz, meth- [illegible] oxy and sucrose unit: δ 3.89 (m, 12H, 6 x H-h′, 6 x H-h$^{1'}$), 3.50 (d, 1H, *J* = [illegible] 12.5 Hz), 3.69-3.83 (m, 7H), 4.15-4.22 (m, 2H), 4.89 (d, 1H, *J* = 4 Hz, H- [illegible] 3′), 5.29-5.41 (m, 3H, H-3, 2 x H-a), 5.52 (t, 1H, *J* = 7.5 Hz, H-4′), 6.07 (d, [illegible] 1H, *J* = 4 Hz, H-1); Cbz and diOMe aromatic and alkenyl protons: δ [illegible] 6.27 (dd, 2H, *J* = 31.5 Hz, H-a′, Ha$^{1'}$), 6.83-6.86 (m, 2H, H-f′, H-f$^{1'}$), 7.01- [illegible] 7.10 (m, 4H, H-c′, H-c$^{1'}$, H-g′, H-g$^{1'}$), 7.29-7.47 (m, 5H, H-b, H-c, H-d, [illegible] H-e, H-f), 7.58 (d, 1H, *J* = 7 Hz, H-b′), 7.64 (d, 1H, *J* = 6.5 Hz, H-b$^{1'}$); ^{13}C [illegible] NMR (75.5 MHz, $CDCl_3$): δ -5.15, -5.11, 14.4, 18.5, 19.3, 24.1, 25.8, [illegible] 26.1, 29.2, 56.1, 56.2, 60.6, 62.6, 63.7, 64.3, 66.9, 70.8, 71.0, 71.9, 72.2, 81.7, [illegible] 83.4, 92.0, 99.9, 101.6, 105.0, 109.8, 109.9, 111.2, 115.0, 116.0, 122.9, 123.1, [illegible] 127.4, 127.7, 128.6, 128.8, 135.4, 144.9, 146.1, 149.4, 151.3, 151.6, 154.7, [illegible] 165.9, 166.4.

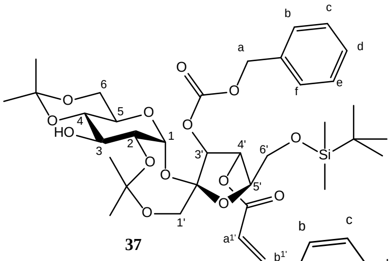

37

Analytical data for compound 37: The ^{1}H NMR data [illegible] of the compound was compared with literature values[3] and [illegible] confirmed to be **37**.

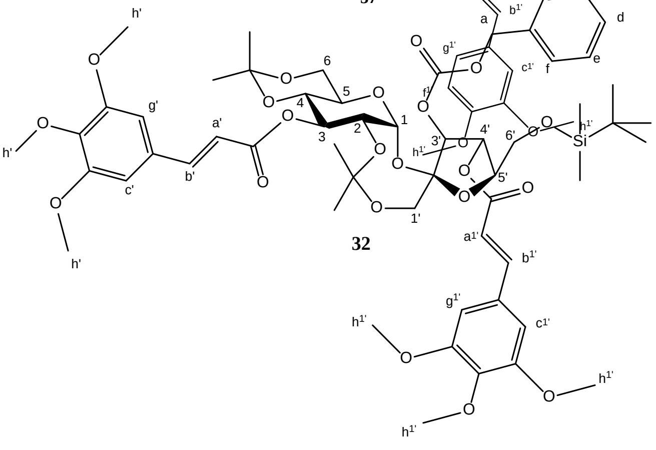

32

Synthesis of 3,4′-di-*O*-(3,4,5- [illegible] trimethoxycinnamoyl) -3′-*O*-carboxybenzyl-6′-*O*-*tert*- [illegible] butyldimethylsiloxy-2,1′-di-*O*-isopropylidene sucrose 32

Following the general procedure, triOMe acid [illegible] (426 mg, 1.79 mmol) gave compound **32** (179 mg, 36% yield) [illegible] and **38** (140 mg, 35% yield) as white solids.

Analytical data for compound 32: ^{1}H NMR (300 [illegible] MHz, $CDCl_3$): TBS group: δ 0.03 (s, 6H, $(CH_3)_2Si$), 0.85 (s, 9H, [illegible] $(CH_3)_3CSi$); isopropylidene rings: δ 1.24-1.46 (m, 12H, 2 x [illegible] $(CH_3)_2C$)); Cbz, methoxy and sucrose unit: δ 3.86-3.87 (m, 18H, 9 [illegible] x H-h′, 9 x H-h$^{1'}$), 3.51 (d, 1H, *J* = 12.5 Hz), 3.69-3.76 (m, 6H), [illegible] 3.92-3.93 (m, 3H), 4.15-4.26 (m, 2H), 4.91 (d, 1H, *J* = 4 Hz, H-3′), [illegible] 5.31-5.43 (m, 3H, H-3, 2 x H-a), 5.55 (t, 1H, *J* = 7.5 Hz, H-4′), [illegible] 6.08 (d, 1H, *J* = 4 Hz, H-1); Cbz and triOMe aromatic & alkenyl [illegible] protons: δ 6.32 (dd, 2H, *J* = 31 Hz, H-a′, H-a$^{1'}$), 6.72-6.75 (m, 4H, [illegible] H-c′, H-c$^{1'}$, H-g′, H-g$^{1'}$), 7.28-7.48 (m, 5H, H-b, H-c, H-d, H-3, H-f), [illegible] 7.56 (d, 1H, *J* = 6 Hz, H-b′), 7.61 (d, 1H, *J* = 6 Hz, H-b$^{1'}$); ^{13}C NMR [illegible] (75.5 MHz, $CDCl_3$): -5.16, -5.12, 1.24, 18.5, 19.3, 24.1, 25.8, 26.1, [illegible] 29.2, 56.4, 60.6, 61.2, 62.6, 63.7, 64.3, 66.9, 70.9, 71.1, 71.9, 72.2, [illegible] 81.7, 83.3, 92.0, 99.9, 101.6, 105.0, 105.6, 116.6, 117.6, 128.6, 128.8, [illegible] 129.9, 130.2,

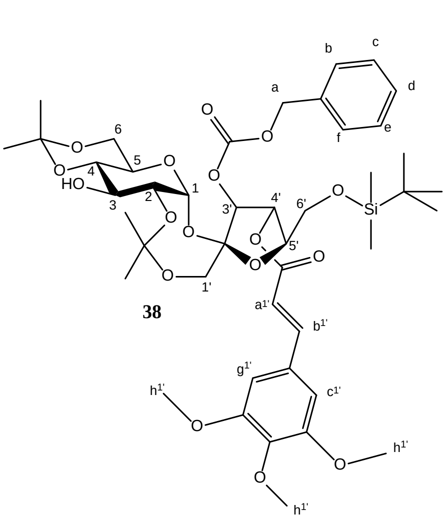

38

135.3, 140.4, 140.6, 145.0, 146.1, 153.7, 154.7, 165.6, 166.1.

Analytical data for compound 38: The ^{1}H NMR data of the compound was compared with literature values[3] and confirmed to be **38.**

Synthesis of 3,4′-di-*O*-coumaroyl-2,1′-di-*O*-isopropylidene sucrose, 3,4′-coum 39

To a stirred solution of compound **27** (100 mg, 0.08 mmol) and $Pd(OAc)_2$ (1.6 mg, 0.007 mmol) in CH_2Cl_2 (1.0 ml) was added NEt_3 (1.6 µL, 0.0112 mmol) and Et_3SiH (18 µL, 0.112 mmol) at room temperature and the mixture was left to stir for 12 hours. The reaction mixture was then diluted with EtOAc (10.0 ml), washed with water (2 x 10 mL) and the organic layer was dried over anhydrous $MgSO_4$ and the solvent evaporated. The crude product obtained was re-dissolved in pyridine (1.0 ml). NEt_3 (22 µL, 0.16 mmol) and 1.56 M $3HF{\cdot}NEt_3$ (154 µL, 0.24mmol) were added to the solution which was left to stir for 12 hours at room temperature. Upon reaction completion (TLC), pyridine was evaporated under reduced pressure and the crude product was subjected to purification using EtOAc/Hexane (3:2) as eluent. 3,4′-coum **39** was obtained as white solid in (28 mg, 49% yield).

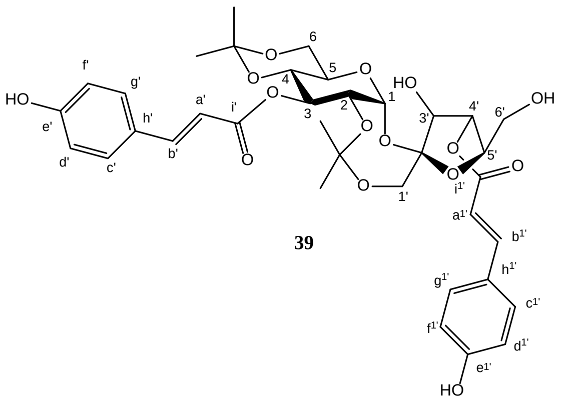

39

Analytical data for 3,4′-coum 39: mp 171-173 °C; ^{1}H NMR (300 MHz, Acetone-d6): isopropylidene rings: δ 1.25-1.47 (m, 12H, 2 x $(CH_3)_2C$)); sucrose unit: δ 3.52-3.56 (m, 1H, H-1′), 3.72-3.83 (m, 4H, H-2, H-4, H-6, H-6′), 4.00-4.07 (m, 5H, H-5, H-6, H-6′, H-1′, H-3′), 4.12 (d, 1H, *J* = 13 Hz, H-5′), 5.10 (d, 1H, *J* = 11 Hz), 5.38 (t, 1H, *J* = 19 Hz, H-3), 5.55 (t, 1H, *J* = 14.5 Hz, H-4′), 6.21 (d, 1H, *J* = 4 Hz, H-1); aromatic and alkenyl protons: δ 6.37 (dd, 2H, *J* = 31 Hz, H-a′, H-a$^{1'}$), 6.90 (dd, 4H, *J* = 11 Hz, H-d′, H-d$^{1'}$, H-f′, H-f$^{1'}$), 7.54-7.71 (m, 6H, H-c′, H-c$^{1'}$, H-g′, H-g$^{1'}$, H-b′, H-b$^{1'}$); ^{13}C NMR (75.5 MHz, Acetone-d6): δ 19.4, 24.5, 25.6 (4 x $(CH_3)_2C$), 60.7 (C-6), 62.7 (C-5), 64.1 (C-6′), 64.5, 66.9 (C-1′), 71.5 (C-3), 72.7 (C-4), 72.9 (C-2), 78.1 (C-4′), 78.7 (C-3′), 83.1 (C-5′), 92.4 (C-1), 100.3, 102.1 (2 x $(CH_3)_2C$), 104.7 (C-2′), 115.0 (C-a′, C-a$^{1'}$), 115.8, 116.2, 116.8, 116.9 (C-d′, C-d$^{1'}$, C-f′, C-f$^{1'}$), 126.9, 127.1 (C-h′, C-h$^{1'}$), 129.5, 130.2, 131.0, 131.2 (C-c′, C-c$^{1'}$, C-g′, C-g$^{1'}$), 145.4, 146.5 (C-b′, C-b$^{1'}$), 160.8, 161.0 (C-e′, C-e$^{1'}$), 166.7, 167.5 (C-i′, C-i$^{1'}$); HRMS (ESI positive mode): found *m*/*z* 737.2399 $[M+Na]^+$, calcd.737.2421 for $C_{36}H_{42}O_{15}Na$.

Synthesis of 3,4′-di-*O*-feruloyl-2,1′-di-*O*-isopropylidene sucrose, 3,4′-feru 40

To a stirred solution of compound **28** (100 mg, 0.08 mmol) and $Pd(OAc)_2$ (1.6 mg, 0.007 mmol) in CH_2Cl_2 (1.0 ml) was added NEt_3 (1.6 µL, 0.0112 mmol) and Et_3SiH (18 µL, 0.112 mmol) at room temperature and the mixture was left to stir for 12 hours. The reaction was diluted with EtOAc (10.0 ml), washed with water (2 x 10 ml) and the organic layer was dried over anhydrous $MgSO_4$ and the solvent evaporated. The crude product obtained was re-dissolved in pyridine (1.0 ml). NEt_3 (22 µL, 0.16 mmol) and 1.56 M $3HF{\cdot}NEt_3$ (154 µL, 0.24mmol) were added to the solution which was left to stir for 12 hours at room temperature. Upon reaction completion (TLC), pyridine was evaporated under reduced pressure and the crude mixture was subjected to purification using 3:2 EtOAc/Hexane as eluent. 3,4′-feru **40** was obtained as white solid (16 mg, 26% yield).

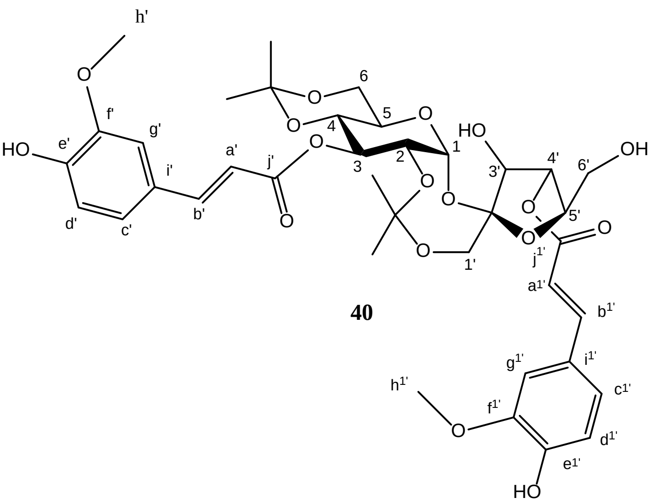

40

Analytical data for 3,4′-feru 40: mp 131-133 °C; ^{1}H NMR (300 MHz, $CDCl_3$): isopropylidene rings: δ 1.29-1.46 (m, 12H, 2 x $(CH_3)_2C$)); methoxy and sucrose unit: δ 3.86- 3.92 (m, 6H, 3 x H-h′, 3 x H-h$^{1'}$), 3.49-3.53 (m, 1H, H-1′), 3.69-3.78, (m, 4H, H-2, H-4, H-6, H-6′), 3.98-4.14 (m, 5H, H-5, H-6, H-6′, H-1′, H-3′), 4.21 (d, 1H, *J* = 12.5 Hz, H-5′), 5.39 (t, 1H, *J* = 19 Hz, H-3), 5.54 (t, 1H, *J* = 14 Hz, H-4′), 6.25 (d, 1H, *J* = 4 Hz, H-1); aromatic and alkenyl protons: δ 6.26 (d, 1H, *J* = 4 Hz, H-a′), 6.31 (d, 1H, *J* = 4 Hz, H-a$^{1'}$), 6.91 (d, 2H, *J* = 8 Hz, H-d′, H-d$^{1'}$), 7.01-7.08 (m, 4H, H-c′, H-c$^{1'}$, H-g′, H-g$^{1'}$), 7.63 (dd, 2H, *J* = 28.5 Hz, H-b′, H-b$^{1'}$) ; ^{13}C NMR (75.5 MHz, $CDCl_3$): δ 19.1, 22.7, 24.2, 25.4 (4 x $(CH_3)_2C$), 56.1, 56.2 (2 x OCH_3), 62.1 (C-6), 62.5 (C-5), 64.6 (C-6′), 66.4 (C-1′), 70.7

(C-3), 71.6 (C-2), 71.7 (C-4), 78.4 (C-4′), 82.8 (C-3′, C-5′), 91.8 (C-1), 100.0, 101.9 (2 x $(CH_3)_2C$), 103.9 (C-2′), 109.7, 109.8 (C-g′, C-g$^{1'}$), 114.4, 115.0 (C-a′, C-a$^{1'}$), 115.4 (C-d′, C-d$^{1'}$), 123.2, 123.5 (C-c′, C-c$^{1'}$), 126.9, 127.1 (C-i′, C-i$^{1'}$), 145.3 (C-b′, C-b$^{1'}$), 146.6, 147.0 (C-f′, C-f$^{1'}$), 148.2, 148.5 (C-e′, C-e$^{1'}$), 166.5, 167.3 (C-j′, C-j$^{1'}$); HRMS (ESI positive mode): found *m/z* 797.2607 $[M+Na]^+$, calcd.797.2633 for $C_{38}H_{46}O_{17}Na$.

Synthesis of 3,4′-di-*O*-sinapoyl-2,1′-di-*O*-isopropylidene sucrose, 3,4′-sinap 41

To a stirred solution of compound **29** (100 mg, 0.08 mmol) and $Pd(OAc)_2$ (1.6 mg, 0.007 mmol) in CH_2Cl_2 (1.0 ml) was added NEt_3 (1.6 μL, 0.0112 mmol) and Et_3SiH (18 μL, 0.112 mmol) at room temperature and left to stir for 12 hours. The reaction mixture was then diluted with EtOAc (10.0 ml), washed with water (2 x 10 mL) and the organic layer was dried over anhydrous $MgSO_4$ and the solvent evaporated. The crude product obtained was re-dissolved in pyridine (1.0 ml). NEt_3 (22 μL, 0.16 mmol) and 1.56 M $3HF{\cdot}NEt_3$ (154 μL, 0.24mmol) were added to the solution and was left to stir for 12 hours at room temperature. Upon reaction completion (TLC), pyridine was removed under reduced pressure and the crude product was subjected to purification using 3:2 EtOAc/Hexane as eluent. 3,4′-sinap **41** was obtained as white solid (17 mg, 26% yield).

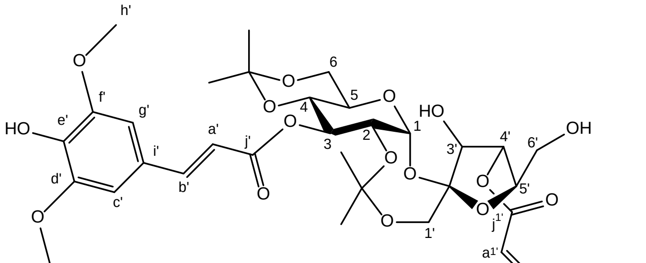

41

Analytical data for 3,4′-sinap 41: mp 143-145 °C; ^1H NMR (300 MHz, $CDCl_3$): isopropylidene rings: δ 1.29-1.45 (m, 12H, 2 x $(CH_3)_2C$)); methoxy and sucrose unit: δ 3.91 (m, 12H, 6 x H-h′, 6 x H-h$^{1'}$), 3.48-3.52 (m, 1H, H-1′), 3.65-3.77 (m, 4H, H-2, H-4, H-6, H-6′), 3.95-4.23 (m, 6H, H-5, H-6, H-6′, H-1′, H-3′, H-5′), 5.39 (t, 1H, *J* = 19 Hz, H-3), 5.56 (t, 1H, *J* = 14 Hz, H-4′), 6.24 (d, 1H, *J* = 4 Hz, H-1); aromatic and alkenyl protons: δ 6.25 (d, 1H, *J* = 6 Hz, H-a′), 6.29 (d, 1H, *J* = 6 Hz, H-a$^{1'}$), 6.75 (d, 4H, *J* = 2 Hz, H-c′, H-c$^{1'}$, H-g′, H-g$^{1'}$), 7.59 (dd, 2H, *J* = 30 Hz, H-b′, H-b$^{1'}$) ; ^{13}C NMR (75.5 MHz, $CDCl_3$): δ 19.1, 23.2, 25.4, 29.1(4 x $(CH_3)_2C$), 56.5, 60.6 (4 x OCH_3), 62.1 (C-6), 62.5 (C-5), 64.5 (C-6′), 66.4 (C-1′), 70.7 (C-3), 71.6 (C-2), 71.7 (C-4), 77.4 (C-4′), 78.4 (C-3′), 82.7 (C-5′), 91.8 (C-1), 100.0, 102.0 (2 x $(CH_3)_2C$), 103.9 (C-2′), 105.3, 105.5 (C-c′, C-c$^{1'}$, C-g′, C-g$^{1'}$), 114.8, 115.8 (C-a′, C-a$^{1'}$), 125.8, 126.0 (C-i′, C-i$^{1'}$), 126.9, 127.2 (C-e′, C-e$^{1'}$), 145.5, 146.8, 147.4 (C-b′, C-b$^{1'}$, C-d′, C-d$^{1'}$, C-f′, C-f$^{1'}$), 166.3, 167.2 (C-j′, C-j$^{1'}$); HRMS (ESI positive mode): found *m/z* 857.2885 $[M+Na]^+$, calcd.857.2844 for $C_{40}H_{50}O_{19}Na$.

Synthesis of 3,4′-di-*O*-caffeoyl-2,1′-di-*O*-isopropylidene sucrose, 3,4′-caff 42

To a stirred solution of compound **30** (100 mg, 0.07 mmol) and $Pd(OAc)_2$ (1.4 mg, 0.006 mmol) in CH_2Cl_2 (1.0 ml) was added NEt_3 (1.3 μL, 0.0092 mmol) and Et_3SiH (15 μL, 0.092 mmol) at room temperature and the mixture was left to stir for 12 hours. The reaction mixture was then diluted with EtOAc (10.0 ml), washed with water (2 x 10 mL) and the organic layer was dried over anhydrous $MgSO_4$ and the solvent evaporated. The crude product obtained was re-dissolved in pyridine (1.0 ml). NEt_3 (20 μL, 0.14 mmol) and 1.56 M $3HF{\cdot}NEt_3$ (135 μL, 0.21mmol) were added to the solution which was left to stir for 12 hours at room temperature. Upon reaction completion (TLC), pyridine was evaporated under reduced pressure and the crude product was subjected to purification using 3:2 EtOAc/Hexane as eluent. 3,4′-caff **42** was obtained as yellow solid (11 mg, 22% yield).

Analytical data for 3,4′-caff 42: mp 165-167 °C; ^1H NMR (300 MHz, Acetone-d6): isopropylidene rings: δ 1.24-1.46 (m, 12H, 2 x $(CH_3)_2C$)); sucrose unit: δ 3.52-3.56 (m, 1H, H-1′), 3.71-3.83 (m, 4H, H-2, H-4, H-6, H-6′), 3.98-4.07 (m, 5H, H-5, H-6, H-6′, H-1′, H-3′), 4.15 (d, 1H, *J* = 12.5 Hz, H-5′), 5.11 (d, 1H, *J* = 11 Hz), 5.38 (t, 1H, *J* = 19 Hz, H-3), 5.55 (t, 1H, *J* = 14.5 Hz, H-4′), 6.21 (d, 1H, *J* = 4 Hz, H-1); aromatic and alkenyl protons: δ 6.30 (dd, 2H, *J* = 30 Hz, H-a′, H-a$^{1'}$), 6.85-6.89 (m, 2H, H-f′, H-f$^{1'}$), 7.03-7.06 (m, 2H H-g′, H-g$^{1'}$), 7.18 (dd, 2H, *J* = 10 Hz, H-c′, H-c$^{1'}$), 7.58 (dd, 2H, *J* = 37 Hz, H-b′, H-b$^{1'}$) ; ^{13}C NMR (75.5 MHz, Acetone-d6): δ 18.4, 23.6, 24.6 (4 x $(CH_3)_2C$), 61.8 (C-6), 63.1 (C-5), 63.5 (C-6′), 63.9 (C-1′), 70.5 (C-3), 71.7 (C-2), 71.9 (C-4), 77.1 (C-4′), 77.7 (C-3′), 82.1 (C-5′), 91.4 (C-1), 99.3, 101.1(2 x $(CH_3)_2C$), 103.7 (C-2′),

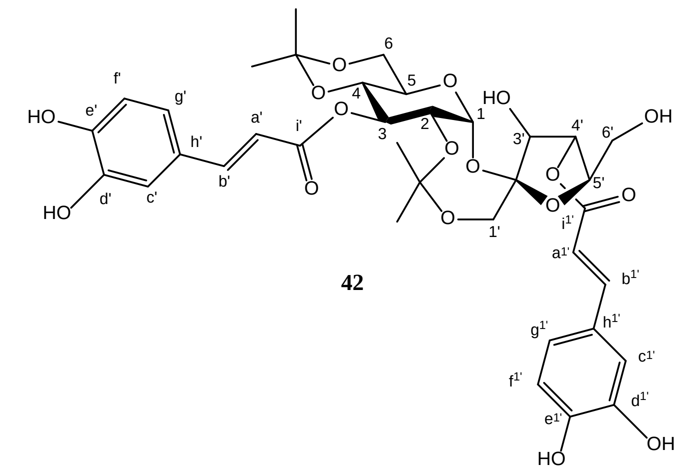

42

114.0, 114.3, (C-a′, C-a$^{1'}$), 114.9, 115.5 (C-c′, C-c$^{1'}$, C-f′, C-f$^{1'}$), 121.6, 121.9 (C-g′, C-g$^{1'}$), 126.6, 126.7 (C-h′, C-h$^{1'}$), 144.8, 145.5, 145.8, 147.9, 148.2 (C-b′, C-b$^{1'}$, C-d′, C-d$^{1'}$, C-e′, C-e$^{1'}$), 165.7, 166.5 (C-i′, C-i$^{1'}$); HRMS (ESI positive mode): found *m*/*z* 747.2499 $[M+H]^+$, calcd.747.2500 for $C_{36}H_{43}O_{17}$.

Synthesis of 3,4′-di-*O*-(3,4-dimethoxycinnamoyl)-2,1′-di-*O*-isopropylidene sucrose, 3,4′-diOMe 43

To a stirred solution of compound **31** (100 mg, 0.096 mmol) and $Pd(OAc)_2$ (1.8 mg, 0.008 mmol) in CH_2Cl_2 (1.0 ml) was added NEt_3 (1.8 µL, 0.0128 mmol) and Et_3SiH (20 µL, 0.128 mmol) at room temperature and the mixture was left to stir for 12 hours. The reaction mixture was then diluted with EtOAc (10.0 ml), washed with water (2 x 10 mL) and the organic layer was dried over anhydrous $MgSO_4$ and the solvent evaporated. The crude product obtained was re-dissolved in pyridine (1.0 ml). NEt_3 (27 µL, 0.19 mmol) and 1.56 M 3HF·NEt_3 (186 µL, 0.29mmol) were added to the solution and was left to stir for 12 hours at room temperature. Upon reaction completion (TLC), pyridine was evaporated under reduced pressure and the crude product was then subjected to purification using 3:2 EtOAc/Hexane as eluent. 3,4′-diOMe **43** was obtained as white solid (23 mg, 30% yield).

Analytical data for 3,4′-diOMe 43: mp 127-129 °C; ^{1}H NMR (300 MHz, $CDCl_3$): isopropylidene rings: δ 1.28-1.44 (m, 12H, 2 x $(CH_3)_2C$)); methoxy and sucrose unit: δ 3.88 (m, 12H, 6 x H-h′, 6 x H-h$^{1'}$), 3.47-3.51 (m, 1H, H-1′), 3.67- 3.76 (m, 4H, H-2, H-4, H-6, H-6′), 3.92-4.11 (m, 5H, H-5, H-6, H-6′, H-1′, H-3′), 4.20 (d, 1H, *J* = 12.5 Hz, H-5′), 5.37 (t, 1H, *J* = 19 Hz, H-3), 5.54 (t, 1H, *J* = 14 Hz, H-4′), 6.24 (d, 1H, *J* = 4 Hz, H-1); aromatic and alkenyl protons: δ 6.29 (dd, 2H, *J* = 22.5 Hz, H-a′, H-a$^{1'}$), 6.82-6.84 (m, 2H, H-f′, H-f$^{1'}$), 7.02-7.10 (m, 4H, H-c′, H-c$^{1'}$, H-g′, H-g$^{1'}$), 7.63 (dd, 2H, *J* = 30 Hz, H-b′, H-b$^{1'}$) ; ^{13}C NMR (75.5 MHz, $CDCl_3$): δ 19.1, 23.1, 23.9, 25.4 (4 x $(CH_3)_2C$), 56.1, 56.2, 60.6 (4 x OCH_3), 62.1 (C-6), 62.5 (C-5), 64.5 (C-6′), 66.4 (C-1′), 70.7 (C-3), 71.6 (C-2), 71.7 (C-4), 77.4 (C-4′), 78.4 (C-3′), 82.8 (C-5′) , 91.8 (C-1), 100.0, 101.9 (2 x $(CH_3)_2C$), 103.9 (C-2′), 109.8, 110.0, 111.2, 111.5 (C-c′, C-c$^{1'}$, C-f′, C-f$^{1'}$), 114.8, 115.8 (C-a′, C-a$^{1'}$), 122.9 (C-g′, C-g$^{1'}$), 127.3, 127.6 (C-i′, C-i$^{1'}$), 145.1, 146.4 (C-b′, C-b$^{1'}$), 149.4, 149.9 (C-e′, C-e$^{1'}$), 151.3, 151.6 (C-d′, C-d$^{1'}$), 166.4, 167.2 (C-j′, C-j$^{1'}$); HRMS (ESI positive mode): found *m*/*z* 825.2903 $[M+Na]^+$, calcd.825.2946 for $C_{40}H_{50}O_{17}Na$.

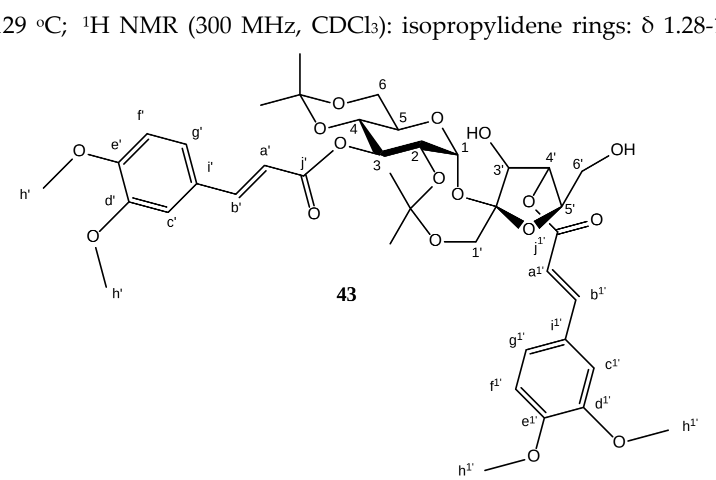

Synthesis of 3,4′-di-*O*-(3,4,5-trimethoxycinnamoyl)-2,1′-di-*O*-isopropylidene sucrose, 3,4′-triOMe 44

To a stirred solution of compound **32** (100 mg, 0.09 mmol) and $Pd(OAc)_2$ (1.7 mg, 0.0074 mmol) was added NEt_3 (1.7 µL, 0.0118 mmol) and Et_3SiH (19 µL, 0.118 mmol) at room temperature and the mixture was left to stir for 12 hours. The reaction mixture was then diluted with EtOAc (10.0 ml), washed with water (2 x 10 mL) and the organic layer was dried over anhydrous $MgSO_4$ and the solvent evaporated. The crude product obtained was re-dissolved in pyridine (1.0 ml). NEt_3 (25 µL, 0.18 mmol) and 1.56 M 3HF·NEt_3 (173 µL, 0.27mmol) were added to the solution which was left to stir for 12 hours at room temperature. Upon reaction completion (TLC), pyridine was evaporated under reduced pressure and the crude product was subjected to purification using 3:2 EtOAc/Hexane as eluent. 3,4′-triOMe **44** was obtained as white solid in (24 mg, 31% yield).

Analytical data for 3,4′-triOMe 44: mp 121-123 °C; ^{1}H NMR (300 MHz, $CDCl_3$): isopropylidene rings: δ 1.29-1.45 (m, 12H, 2 x $(CH_3)_2C$)); methoxy and sucrose unit: δ 3.84-3.86 (m, 18H, 9 x H-h′, 9 x H-h$^{1'}$), 3.48-3.52 (m, 1H, H-1′), 3.67-3.77 (m, 4H, H-2, H-4, H-6, H-6′), 3.93-4.11 (m, 5H, H-5, H-6, H-6′, H-1′, H-3′), 4.21 (d, 1H, *J* = 12.5 Hz, H-5′), 5.40 (t, 1H, *J* = 19 Hz, H-3), 5.57 (t, 1H, *J* = 14 Hz, H-4′), 6.24 (d, 1H, *J* = 4 Hz, H-1); aromatic and alkenyl protons: δ 6.33 (dd, 2H, *J* = 21.5 Hz, H-a′, H-a$^{1'}$), 6.74-6.75 (m, 4H, H-c′, H-c$^{1'}$, H-g′, H-g$^{1'}$), 7.60 (dd, 2H, *J* = 28.5 Hz, H-b′, H-b$^{1'}$) ; ^{13}C NMR (75.5 MHz, $CDCl_3$): δ 19.2, 24.3, 25.5, 29.2 (4 x $(CH_3)_2C$), 56.4, 61.2 (6 x OCH_3), 62.1 (C-6), 62.5 (C-5), 64.6 (C-6′), 66.4 (C-1′), 70.8 (C-3), 71.6 (C-2), 71.7 (C-4), 77.4 (C-4′), 78.4 (C-3′), 82.8 (C-5′), 91.9 (C-1), 100.1, 102.0 (2 x $(CH_3)_2C$), 103.9 (C-2′), 105.5, 105.6 (C-c′, C-c$^{1'}$,

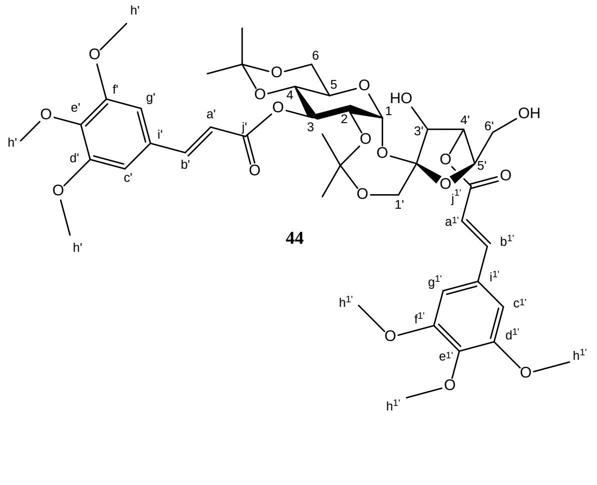

C-g′, C-g1′), 116.4, 117.3 (C-a′, C-a1′), 129.0, 129.8 (C-i′, C-i1′), 140.4, 140.6 (C-e′, C-e1′), 146.5 (C-b′, C-b1′), 153.7 (C-d′, C-d1′, C-f′, C-f1′), 166.2, 167.0 (C-j′, C-j1′); HRMS (ESI positive mode): found *m/z* 863.3331 [M+H]$^+$, calcd.863.3338 for $C_{42}H_{55}O_{19}$.

Synthesis of 4′-*O*-para-nitrobenzoyl-3′-*O*-carboxybenzyl-6′-*O*-tert-butyldimethylsiloxy-2,1′-di-*O*-isopropylidene sucrose, 4′-*O*-PNB 45

The synthesis of 4′-*O*-PNB was accomplished according to the literature procedure.[3] 3′-*O*-Cbz **26** (500 mg, 0.746 mmol) and DMAP (9.1 mg, 0.0746 mmol) were dissolved in CH_2Cl_2 (15 mL) and stirred at 4 °C in an ice bath. Subsequently, NEt_3 (312 µL, 2.24 mmol) was added fol-lowed by dropwise addition of a solution of PNBCl (208 mg, 1.12 mmol) in CH_2Cl_2 (5 mL). The reaction was left to stir for 12 hours (TLC). The reac-tion mixture was evaporated, and the crude product was purified using column chromatography using Hexane/EtOAc (4:1) as elu-ent. 4′-*O*-PNB **45** (428 mg, 70% yield) was obtained as White solid. The ^{1}H NMR data of 4′-*O*-PNB **45** confirmed to the literature data.[3]

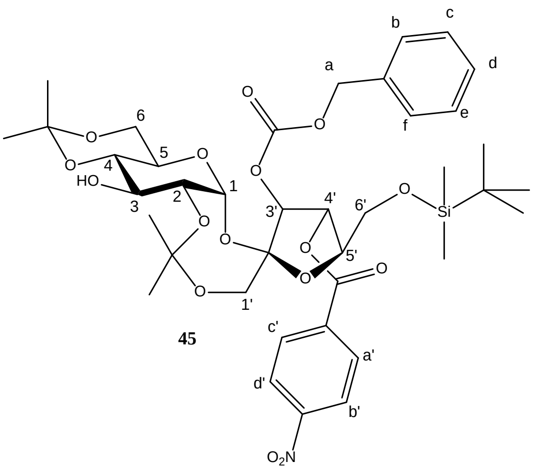

45

Synthesis of 3′-*O*-carboxybenzyl-4′-*O*-para-nitrobenzoyl - 2,1′-di-*O*-isopropylidene sucrose, 3′-*O* -Cbz-4′-*O*-PNB 46

To a stirred solution of 4′-*O*-PNB **45** (1.00 g, 1.22 mmol) in pyridine (10.0 ml) was added NEt_3 (339 µL, 2.44 mmol) and 1.56 M 3HF·NEt_3 (2.35ml, 3.66mmol) and the reaction was left to stir for 12 hours at room temperature (TLC). Pyridine was removed, and the crude product was subjected to column chromatography purification using EtOAc/Hexane (3:2) as the eluent. 3′-*O* -Cbz-4′-*O*-PNB **46** (740 mg, 86% yield) was obtained as white solid.

Analytical data for 3′-*O* -Cbz-4′-*O*-PNB 46: ^{1}H NMR (300 MHz, $CDCl_3$): isopropylidene rings: δ 1.36-1.55 (m, 12H, 2 x $(CH_3)_2C$)); sucrose unit: δ 3.15 (dd, 1H, *J* = 15 Hz), 3.54-3.88 (m, 9H), 4.13 (d, 1H, *J* = 12.5 Hz), 4.26-4.27 (m, 1H), 5.17-5.19 (m, 1H, H-3′), 5.88 (t, 1H, *J* = 12 Hz, H-4′), 6.19 (d, 1H, *J* = 3.5 Hz, H-1); Cbz group: δ 5.17-5.19 (m, 2H), 7.30-7.36 (m, 5H); PNB group: δ 8.20 (dd, 4H, *J* = 49 Hz); ^{13}C NMR (75.5 MHz, $CDCl_3$): δ 19.3, 24.3, 25.6, 29.2, 62.0, 64.3, 66.7, 70.1, 70.7, 72.8, 73.7, 76.1, 80.3, 83.1, 91.7, 100.1, 102.3, 103.7, 123.9, 128.5, 128.9, 131.2, 134.6, 134.8, 150.1, 151.1, 154.8, 164.1.

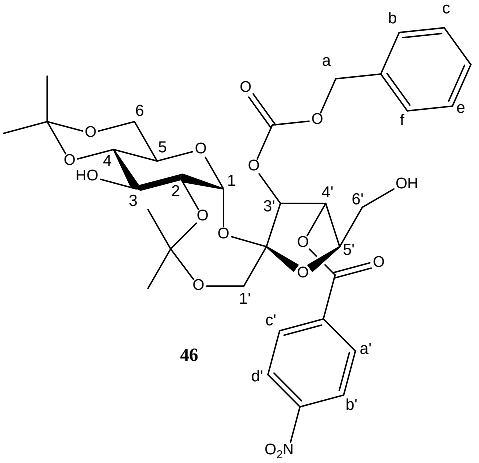

46

1.2.5.3 General procedure for acylation of 3′-*O*-Cbz-4′-*O*-PNB 46

A stirred solution of 3′-*O*-Cbz-4′-*O*-PNB **46** (150 mg, 0.218 mmol) and DMAP (3 mg, 0.02 mmol) in CH_2Cl_2 (15 mL) was heated under reflux for 10 minutes. The (substituted) cinnamic acid (0.872 mmol) and EDC (167 mg, 0.872 mmol) were then added to the refluxing solution which was left under reflux for 2 weeks (TLC). The reaction mixture was then washed with distilled water (3 x 10 mL), the organic phase separated, dried over anhydrous $MgSO_4$ and the solvent evaporated under vacuum. The crude product was purified using column chromatography using Hexane/EtOAc (8:1) as the eluent. The general procedure was used to prepare compounds **47, 48, 49, 50, 51, 52** and **53**. Along with these compounds, a by-product was always obtained, and its analytical data were not recorded.

Synthesis of 3,6′-di-*O*-cinnamoyl - 3′-*O*-carboxybenzyl-4′-*O*-para-nitrobenzoyl -2,1′-di-*O*-isopropylidene sucrose 47

Following the general procedure, reaction using cinnamic acid (129 mg, 0.872 mmol) gave compound **47** (135 mg, 64% yield) and a by-product (40 mg, 22% yield) as white solids.

Analytical data for compound 47: mp 98-101°C; 1H NMR (300 MHz, MeOD): isopropylidene rings: δ 1.24-1.45 (m, 12H, 2 x $(CH_3)_2C$)); Cbz and sucrose unit: δ 3.53-4.52 (m, 11H), 5.08 (d, 1H, *J* = 4 Hz, H-3'), 5.34 (m, 3H, H-3, 2 x H-a), 5.62 (t, 1H, *J* = 8 Hz, H-4'), 6.16 (d, 1H, *J* = 4 Hz, H-1); Cbz and cinnamoyl aromatic and alkenyl protons: δ 6.41 (dd, 2H, *J* = 32 Hz, H-a', H-$a^{1'}$) 7.30-7.52 (m, 15H, H-b, H-c, H-d, H-e, H-f, H-c', H-$c^{1'}$, H-d', H-$d^{1'}$, H-e', H-$e^{1'}$, H-f', H-$f^{1'}$, H-g', H-$g^{1'}$), 7.65 (dd, 2H, *J* = 15 Hz, H-b', H-$b^{1'}$); PNB group: δ 8.18 (dd, 4H, *J* = 24 Hz, H-a^2, H-b^2, H-c^2, H-d^2); 13C NMR (75.5 MHz, $CDCl_3$): δ 19.2, 24.1, 25.7, 29.0, 29.2, 62.3, 64.5, 65.0, 66.6, 71.0, 71.2, 71.8, 72.1, 79.4, 79.9, 80.5, 81.0, 92.1, 99.9, 101.8, 105.1, 105.2, 117.6, 118.3, 123.9, 128.3, 128.8, 128.9, 129.0, 129.1, 130.5, 130.6, 131.2, 134.3, 134.4, 134.5, 134.7, 135.0, 145.0, 145.5, 147.8, 151.0, 154.6, 163.8, 166.0, 166.1, 166.5.

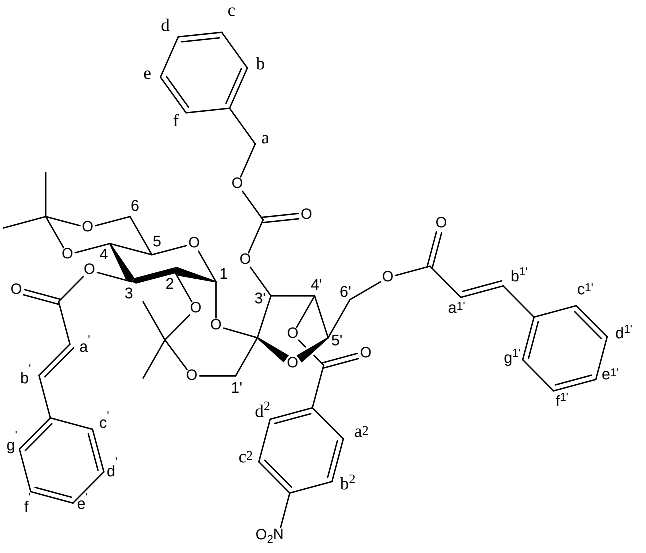

47

Synthesis of 3,6'-di-*O*-(4-acetoxycinnamoyl) -3'-*O*-carboxybenzyl-4'-*O*-para-nitrobenzoyl -2,1'-di-*O*-isopropylidene sucrose 48

Following the general procedure, reaction using OAc-coum acid (177 mg, 0.872 mmol) gave compound **48** (184 mg, 78% yield) and a by-product (29 mg, 15% yield) as white solids.

Analytical data for compound 48: ^{1}H NMR (300 MHz, Acetone-d6): isopropylidene rings: δ 1.22-1.49 (m, 12H, 2 x $(CH_3)_2C$); OAc-coumaroyl acetoxy & methoxy, Cbz and sucrose unit: δ 2.28-2.29 (m, 6H, 3 x H-i', 3 x H-$i^{1'}$), 3.64-4.50 (m, 7H), 4.25-4.32 (m, 1H), 4.45-4.50 (m, 1H), 4.52-4.62 (m, 1H), 4.70 (m, 1H), 5.29-5.33 (m, 2H, 2 x H-a), 5.37 (d, 1H, *J* = 4 Hz, H-3'), 5.44 (t, 1H, *J* = 18.5 Hz, H-3), 5.77 (t, 1H, *J* = 7.5 Hz, H-4'), 6.20 (d, 1H, *J* = 4 Hz, H-1); Cbz and OAc-coumaroyl aromatic and alkenyl protons: δ 6.49-6.59 (m, 2H, H-a', H-$a^{1'}$), 7.16-7.50 (m, 9H, H-b, H-c, H-d, H-e, H-f, H-d', H-$d^{1'}$ H-f', H-$f^{1'}$), 7.68-7.87 (m, 6H, H-c', H-$c^{1'}$ H-g', H-$g^{1'}$, H-b', H-$b^{1'}$; PNB group: δ 8.35 (dd, 4H, *J* = 26 Hz, H-a^2, H-b^2, H-c^2, H-d^2); ^{13}C NMR (75.5 MHz, Acetone-d6): δ 11.3, 14.3, 19.4, 20.9, 21.0, 23.7, 24.2, 24.3, 24.5, 25.7, 39.7, 55.0, 62.6, 65.2, 65.4, 67.1, 71.2, 71.3, 71.8, 72.4, 80.6, 80.7, 81.8, 92.8, 100.3, 102.2, 118.6, 117.9, 118.6, 119.2, 123.2, 123.4, 129.3, 129.4, 129.5, 129.6, 130.2, 130.3, 130.6, 132.0, 132.7, 132.8, 132.9, 133.0, 133.5, 136.3, 136.4, 144.5, 144.8, 146.6, 153.5, 153.9, 155.1, 164.8, 165.4, 166.3, 166.8, 165.4, 166.3, 166.8, 168.0, 169.5, 170.1.

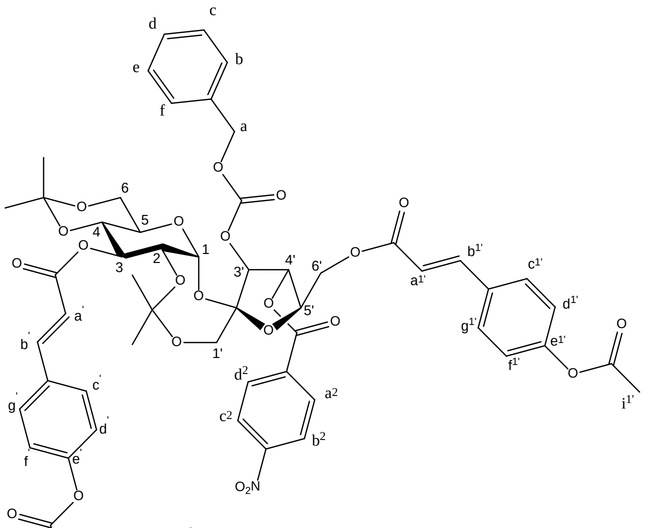

48

Synthesis of 3,6'-di-*O*-(4-acetoxy-3-methoxycinnamoyl) -3'-*O*-carboxybenzyl-4'-*O*-para-nitrobenzoyl -2,1'-di-*O*-isopropylidene sucrose 49

Following the general procedure, reaction using OAc-feru acid (177 mg, 0.872 mmol) gave compound **49** (149 mg, 60% yield) and a by-product (28 mg, 14% yield) as white solids.

Analytical data for compound 49: ^{1}H NMR (300 MHz, Acetone-d6): isopropylidene rings: δ 1.23-1.49 (m, 12H, 2 x $(CH_3)_2C$); Cbz, OAC-feru acetoxy, methoxy and sucrose unit: δ 2.26-2.27 (m, 6H, 3 x H-i', 3 x H$i^{1'}$), 3.89-3.91 (m, 6H, 3 x H-h', 3 x H-$h^{1'}$), 3.65-4.07 (m, 4H), 4.29-4.33, (m, 1H), 4.43-4.50 (m, 1H), 4.56-4.62 (m, 1H), 4.68-4.72 (m, 1H), 5.32 (d, 1H,

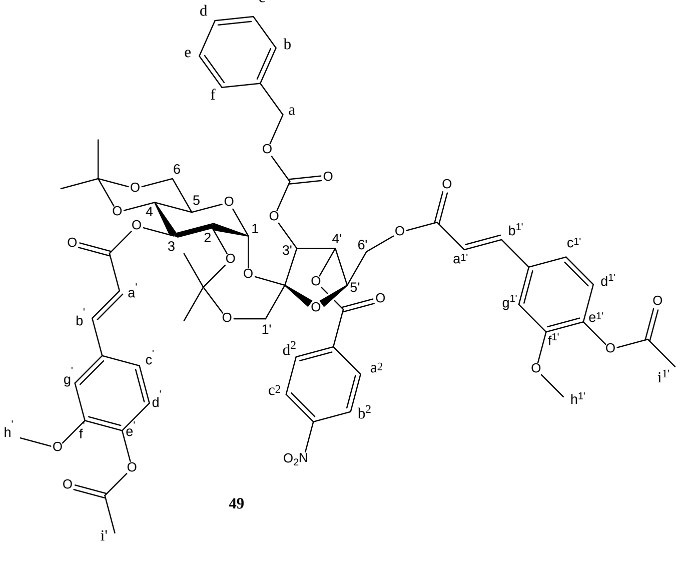

49

J = 4 Hz, H-3′), 5.37- 5.38 (m, 2H, Cbz, 2 x H-a), 5.45 (t, 1H, J = 18.5 Hz, H-3), 5.75 (t, 1H, J = 7.5 Hz, H-4′), 6.20 (d, 1H, J = 4 Hz, H-1); Cbz and OAc- Feru aromatic and alkenyl protons: δ 6.58 (dd, 2H, J = 24.5 Hz, H-a′, H-a1′) 7.08-7.13 (m, 2H, H-d′, H-d1′), 7.20-7.39 (m, 5H, H-b, H-c, H-d, H-e, H-f), 7.45-7.53 (m, 4H, H-c′, H-c1′, H-g′, H-g1′), 7.68 (dd, 2H, J = 22.5 Hz, H-b′, H-b1′); PNB group: δ 8.34 (dd, 4H, J = 26.5 Hz, H-a2, H-b2, H-c2, H-d2); ^{13}C NMR (75.5 MHz, Acetone-d6): δ18.5, 19.6, 23.4, 24.8, 55.5, 61.7, 64.3, 64.5, 66.3, 70.4, 70.8, 71.6, 71.8, 79.0, 79.9, 80.9, 91.9, 99.4, 101.3, 105.4, 111.5, 111.7, 117.8, 118.3, 121.5, 123.3, 123.4, 123.6, 128.4, 128.5, 128.6, 128.7, 131.1, 133.3, 134.8, 135.5, 141.8, 144.0, 144.3, 150.9, 151.9, 154.2, 163.9, 165.5, 166.0, 168.0.

Synthesis of 3,6′-di-*O*-(4-acetoxy-3,5-dimethoxycinnamoyl) -3′-*O*-carboxybenzyl-4′-*O*-para-nitrobenzoyl -2,1′-di-*O*-isopropylidene sucrose 50

Following the general procedure, reaction using OAc-sinap acid (232 mg, 0.872 mmol) gave compound **50** (173 mg, 66% yield) and a by-product (27 mg, 13% yield) as yellow solids.

Analytical data for compound 50: isopropylidene rings: δ 1.17-1.48 (m, 12H, 2 x $(CH_3)_2C$)); OAc-sinap acetoxy, methoxy and sucrose unit: δ 2.26 (m, 6H, 3 x H-I′, 3 x H-i1′), 3.64-3.78 (m, 4H), 3.89 (m, 12H, 6 x H-h′, 6 x H-h1′) 4.29-4.71 (m, 5H), 5.32 (d, 1H, J = 4 Hz, H-3′) 5.38 (s, 2H, Cbz, 2 x H-a), 5.46 (t, 1H, J = 18.5 Hz, H-3), 5.74 (t, 1H, J = 8 Hz, H-4′), 6.20 (d, 1H, J = 3.5 Hz, H-1); aromatic and alkenyl protons: δ 6.61 (dd, 2H, J = 23.5 Hz, H-a′, H-a1′), 7.10 (d, 4H, J = 16 Hz, H-c′, H-c1′ H-g′, H-g1′), 7.35-7.53 (m, 5H, H-b, H-c, H-d, H-e, H-f), 7.79 (d, 2H, J = 16 Hz, H-b′, H-b1′); PNB group: δ 8.34 (dd, 4H, J = 28 Hz, H-a2, H-b2, H-c2, H-d2); ^{13}C NMR (75.5 MHz, $CDCl_3$): 19.9, 20.7, 21.3, 24.7, 26.2, 29.5, 29.8, 30.1, 30.3, 30.6, 30.8, 57.1, 57.2, 61.0, 65.7, 71.8, 73.0, 80.4, 82.3, 100.8, 102.7, 106.4, 106.6, 107.0, 118.5, 119.2, 119.7, 125.0, 129.8, `129.9, 130.0, 132.4, 132.7, 133.5, 134.0, 134.1, 136.2, 136.9, 146.2, 149.6, 154.1, 154.2, 163.9, 165.3, 166.9, 168.8.

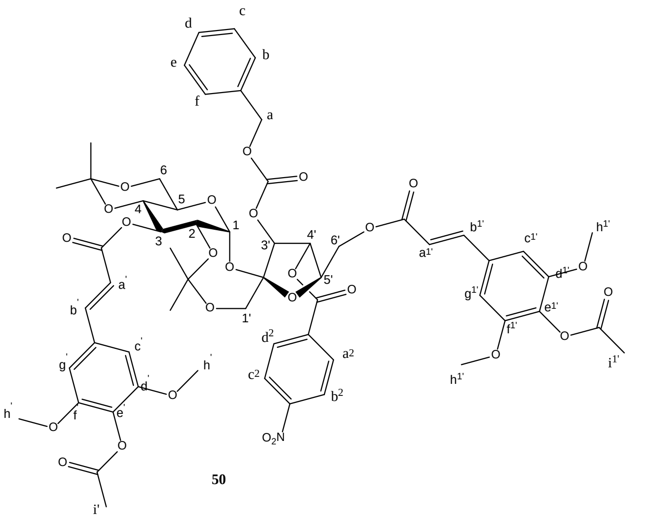

50

Synthesis of 3,6′-di-*O*-(3,4-di-*tert*-butyldimethylsiloxycinnamoyl) -3′-*O*-carboxybenzyl-4′-*O*-para-nitrobenzoyl -2,1′-di-*O*-isopropylidene sucrose, 3,6′-TBS -caff 51

Following the general procedure, reaction using OTBS-caff acid (227 mg, 0.872 mmol) gave compound **51** (198 mg, 61% yield) and a by-product (84 mg, 35% yield) as white solids.

Analytical data for compound 51: TBS group: δ 0.22 (m, 24H), 0.98 (m, 36H); isopropylidene rings: δ 1.20-1.46 (m, 12H, 2 x $(CH_3)_2C$)); sucrose unit: δ 3.62-4.67 (m, 12H), 5.28 (d, 1H, J = 4 Hz, H-3′), 5.35 (m, 2H, 2 x H-a), 5.44 (t, 1H, J = 19 Hz, H-3), 5.73 (t, 1H, J = 7.5 Hz, H-4′), 6.18 (d, 1H, J = 3.5 Hz, H-1); aromatic and alkenyl protons: δ 6.27-6.38 (m, 4H, H-a′, H-a1′), 6.87-6.94 (m, 5H, H-d′, H-d1′), 7.10-7.19 (m, 9H, , H-c′, H-c1′, H-g′, H-g1′), 7.21-7.74 (m, 13H, H-b, H-c, H-d, H-e, H-f, H-b′, H-b1′); PNB group: δ 8.31 (dd, 4H, J = 25 Hz, H-a2, H-b2, H-c2, H-d2); ^{13}C NMR (75.5 MHz, $CDCl_3$): δ -5.32, -4.81, -3.92, 0.63, 10.4, 13.5, 17.5, 17.8, 18.5, 22.8, 23.5, 23.8, 30.4, 38.8, 61.7, 64.4, 66.2, 67.4, 70.3, 71.9, 79.2, 79.9, 80.9, 91.8, 99.5, 101.4, 105.3, 115.0, 115.5,116.1, 117.7, 118.8, 119.8, 120.6, 120.9, 121.4, 122.2, 122.7, 123.6, 131.1, 132.6, 134.8, 135.5,144.8, 147.1, 149.2, 151.0, 154.2, 163.8, 165.7, 166.3, 167.2.

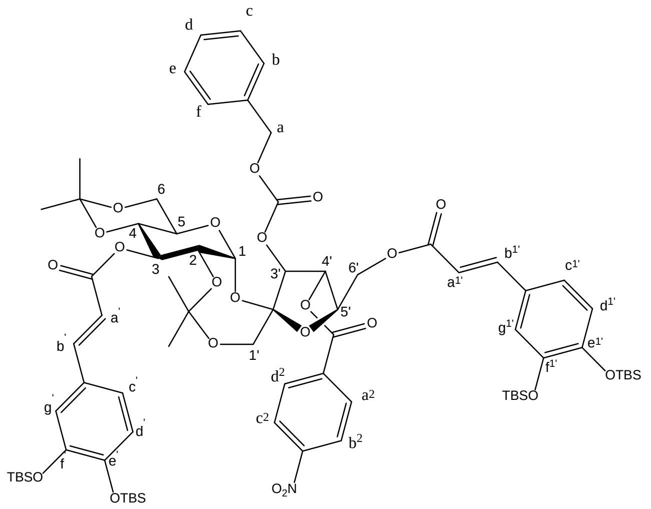

51

Synthesis of 3,6′-di-*O*-(3,4-dimethoxycinnamoyl) -3′-*O*-carboxybenzyl-4′-*O*-para-nitrobenzoyl -2,1′-di-*O*-isopropylidene sucrose 52

Following the general procedure, reaction using diOMe acid (181 mg, 0.872 mmol) gave compound **52** (187 mg, 79% yield) and by-product (37 mg, 19% yield) as white solids.

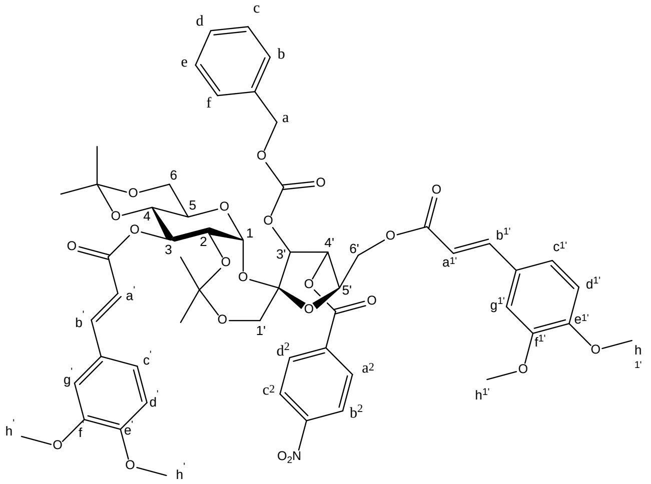

52

Analytical data for compound 52: ^{1}H NMR (300 MHz, CDCl$_3$): isopropylidene rings: δ 1.23-1.46 (m, 12H, 2 x $(CH_3)_2C$)); diOMe methoxy and sucrose unit: δ 3.89 (m, 13H, 6 x H-h′, 6 x H-h$^{1'}$), 3.54-3.73 (m, 4H), 4.00-4.51 (m, 4H), 5.10 (d, 1H, *J* = 4 Hz, H-3′), 5.27-5.43 (m, 3H, H-3, 2 x H-a), 5.63 (t, 1H, *J* = 7 Hz, H-4′), 6.17 (d, 1H, *J* = 4 Hz, H-1); aromatic and alkenyl protons: δ 6.23-6.40 (m, 4H, H-a′, H-a$^{1'}$), 6.80-7.17 (m, 12H, H-d′, H-d$^{1'}$, H-c′, H-c$^{1'}$, H-g′, H-g$^{1'}$), 7.30-7.47 (m, 5H, H-b, H-c, H-d, H-e, H-f), 7.55-7.80 (m, 4H, H-b′, H-b$^{1'}$); PNB group: δ 8.19 (dd, 4H, *J* = 43 Hz, H-a^2, H-b^2, H-c^2, H-d^2); ^{13}C NMR (75.5 MHz, CDCl$_3$): δ 19.3, 25.7, 29.3, 56.1, 56.2, 56.3, 71.2, 99.9, 101.8, 105.2, 110.1, 111.3, 114.6, 122.9, 123.8, 127.0, 128.8, 128.9, 131.2, 135.1, 148.7, 149.6, 152.2, 163.1, 163.9

Synthesis of 3,6′-di-*O*-(3,4,5-trimethoxycinnamoyl) - 3′-*O*-carboxybenzyl-4′-*O*-para-nitrobenzoyl -2,1′-di-*O*-isopropylidene sucrose 53

Following the general procedure, reaction using triOMe acid (208 mg, 0.872 mmol) gave compound **53** (167 mg, 67% yield) and a by-product (12 mg, 6% yield) as white solids.

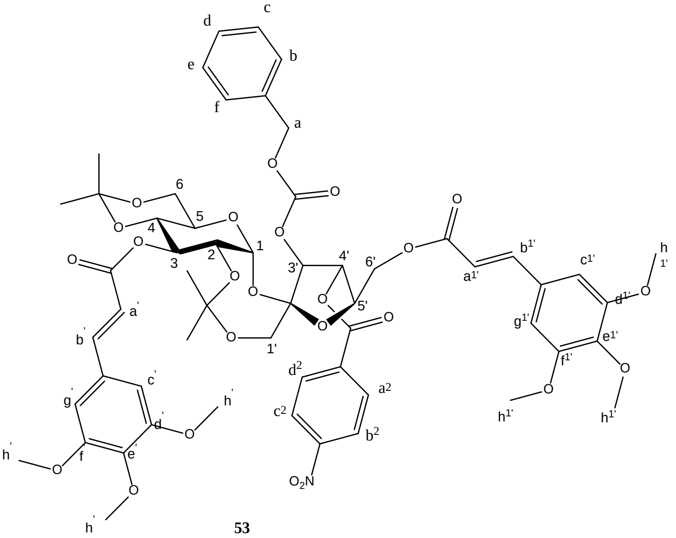

53

Analytical data for compound 53: ^{1}H NMR (300 MHz, CDCl$_3$): isopropylidene rings: δ 1.26-1.46 (m, 12H, 2 x $(CH_3)_2C$)); triOMe methoxy and sucrose unit: δ 3.85-3.88 (m, 26H, 9 x H-h′, 9 x H-h$^{1'}$), 3.54-3.76 (m, 4H), 3.90-4.50 (m, 6H), 5.09 (d, 1H, *J* = 4 Hz, H-3′), 5.27-5.42 (m, 3H, H-3, 2 x H-a), 5.61 (t, 1H, *J* = 7 Hz, H-4′), 6.17 (d, 1H, *J* = 4 Hz, H-1); aromatic and alkenyl protons: δ 6.28-6.43 (m, 3H, H-b′, H-b$^{1'}$), 6.69-6.77 (m, 6H H-c′, H-c$^{1'}$ H-g′, H-g$^{1'}$), 7.28-7.47, (m, 5H, H-b, H-c, H-d, H-e, H-f), 7.53-7.77 (m, 3H, H-b′, H-b$^{1'}$); PNB group: δ 8.1 9(dd, 4H, *J* = 43 Hz, H-a^2, H-b^2, H-c^2, H-d^2) ; ^{13}C NMR (75.5 MHz, CDCl$_3$): δ 19.3, 24.1, 25.7, 29.3, 56.4, 61.2, 64.5, 71.2, 72.1, 79.3, 80.5, 81.0, 92.1, 99.9, 101.9, 105.2, 105.5, 106.0, 116.0, 116.9, 117.5, 123.9, 128.8, 128.9, 129.3, 130.0, 131.2, 134.4, 135.0, 140.4, 145.5, 148.9, 151.1, 153.6, 153.7, 154.6, 162.7, 163.9, 166.1, 166.4.

1.2.5.4 General procedure for the deprotection of PNB, Cbz and acetal groups

To a stirred solution of each of the compounds **47, 48, 49, 50, 51, 52,** or **53** in of methanol: THF (1 ml, 9:1) was added $Mg(OMe)_2$. The reaction mixtures were left to stir for 12 hours (TLC) then quenched with 1N HCl. The solvent was evaporated, and the slurries obtained were then dissolved in CH_2Cl_2 (15 mL) and washed with water (2 x 10 mL). The organic layer was separated, dried over anhydrous $MgSO_4$, filtered and the solvent evaporated under reduced pressure. Purification using silica gel column chromatography using Hex:EtOAc (1:1) as the eluent gave the desired compounds 3,6′-cinn **54**, 3,6′-coum **55**, 3,6′-feru **56**, 3,6′-diOMe **59**, 3,6′-triOMe **60**, 3,6′-OAc-sinap **61** and 3,6′-OTBS-caff **62**.

Synthesis of 3,6′-di-*O*-cinnamoyl-2,1′-di-*O*-isopropylidene sucrose, 3,6′-cinn 54

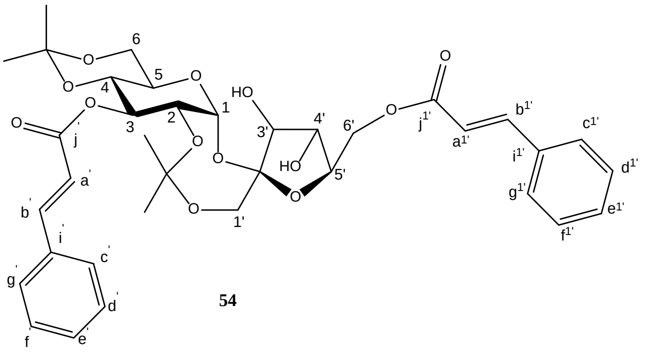

Following the general procedure, compound **47** (100 mg, 0.104 mmol) completely reacted with $Mg(OMe)_2$ (158 μL, 0.052 mmol) before quenching the reaction with 1N HCl (104 μL, 0.104 mmol). The product compound **54** (60 mg, 85 % yield) was obtained as

white solid.

Analytical data for 3,6′-cinn 54: mp 108-110 °C; ^{1}H NMR (300 MHz, $CDCl_3$): isopropylidene rings: δ 1.27-1.45 (m, 12H, 2 x $(CH_3)_2C$)); sucrose unit: δ 3.48-3.52 (m, 1H, H-5), 3.71-3.96 (m, 7H, 2 x H-1′, H-2, H-4, 2 x H-6, H-3′), 4.15-4.34 (m, 3H, H-4′, H-5′, H-6′), 4.46-4.47 (m, 1H, H-6′), 5.39 (t, 1H, *J* = 19 Hz, H-3), 6.16 (d, 1H, *J* = 4 Hz, H-1); aromatic and alkenyl protons: δ 6.43 (dd, 2H, *J* = 26.5 Hz, H-a′, H-a$^{1'}$), 7.34-7.37 (m, 6H, H-e′, H-e$^{1'}$, H-d′, H-d$^{1'}$, H-f′, H-f$^{1'}$), 7.48-7.53 (m, 4H, H-c′, H-c$^{1'}$, H-g′, H-g$^{1'}$), 7.68 (dd, 2H, *J* = 22 Hz, H-b′, H-b$^{1'}$); ^{13}C NMR (75.5 MHz, $CDCl_3$): δ 19.2, 24.3, 25.5, 29.2 (4 x $(CH_3)_2C$), 62.4 (C-6), 64.3 (C-5) , 65.9 (C-6′), 66.3 (C-1′), 71.2 (C-3), 71.9 (C-4), 72.0 (C-2), 78.7 (C-4′), 79.9 (C-3′), 80.1 (C-5′), 91.5 (C-1), 100.1, 101.8 (2 x $(CH_3)_2C$), 104.2 (C-2′), 117.9, 118.1 (C-a′, C-a$^{1'}$), 128.4, 129.1 (C-c′, C-c$^{1'}$, C-g′, C-g$^{1'}$, C-d′, C-d$^{1'}$, C-f′, C-f$^{1'}$), 130.6 (C-e′, C-e$^{1'}$), 134.6 (C-h′, C-h$^{1'}$), 145.4, 145.5 (C-b′, C-b$^{1'}$), 166.6, 167.1 (C-i′, C-i$^{1'}$); HRMS (ESI positive mode): found *m/z* 705.2502 $[M+Na]^+$, calcd.705.2523 for $C_{36}H_{42}O_{13}Na$.

Synthesis of 3,6′-di-*O*-coumaroyl-2,1′-di-*O*-isopropylidene sucrose, 3,6′-coum 55

Following the general procedure, compound **48** (100 mg, 0.092 mmol)) completely reacted with $Mg(OMe)_2$ (140 μL, 0.046 mmol) before quenching the reaction with 1N HCl (92 μL, 0.092 mmol). The product compound **55** (22 mg, 33% yield) was obtained as white solid.

Analytical data for 3,6′-coum 55: mp 159-161°C; isopropy- lidene rings: δ 1.16-1.38 (m, 12H, 2 x $(CH_3)_2C$); su- crose unit: δ 3.32-3.37 (m, 1H, H-5), 3.62-3.99 (m, 7H, 2 x H-1′, H-2, H-4, 2 x H-6, H-3′), 4.05-4.19 (m, 3H, H-4′, H-5′, H-6′), 4.32-4.36 (m, 1H, H-6′), 5.34 (t, 1H, *J* = 19 Hz, H-3), 6.02 (d, 1H, *J* = 4 Hz, H-1); aromatic and alkenyl protons: δ 6.24 (dd, 2H, *J* = 27 Hz, H-a′, H-a$^{1'}$), 6.71 (d, 4H, *J* = 9 Hz, H-d′, H-d$^{1'}$, H-f′, H-f$^{1'}$), 7.36 (d, 4H, *J* = 8.5 Hz, H-c′, H-c$^{1'}$, H-g′, H-g$^{1'}$), 7.52 (dd, 2H, *J* = 29 Hz, H-b′, H-b$^{1'}$); ^{13}C NMR (75.5 MHz, MeOD): δ 21.1, 24.5, 25.4, 29.6 (4 x $(CH_3)_2C$), 63.4 (C-6), 64.9 (C-5), 65.0 (C-6′), 67.1 (C-1′), 67.7 (C-3), 72.6 (C- 4), 73.1 (C-2), 78.1 (C-4′), 80.2 (C-3′), 81.4 (C-5′), 92.6 (C-1), 101.1, 102.8 (2 x $(CH_3)_2C$), 105.5 (C-2′), 115.1 (C-a′, C-a$^{1'}$), 115.4, 117.0 (C-d′, C-d$^{1'}$, C-f′, C-f$^{1'}$), 127.3 (C-h′, C-h$^{1'}$), 131.3, 131.4 (C-c′, C-c$^{1'}$, C-g′, C-g$^{1'}$), 146.8, 146.9 (C-b′, C-b$^{1'}$), 161.5 (C-e′, C-e$^{1'}$), 168.9, 169.1 (C-i′, C-i$^{1'}$); HRMS (ESI positive mode): found *m/z* 737.2454 $[M+Na]^+$, calcd.737.2421 for $C_{36}H_{42}O_{15}Na$.

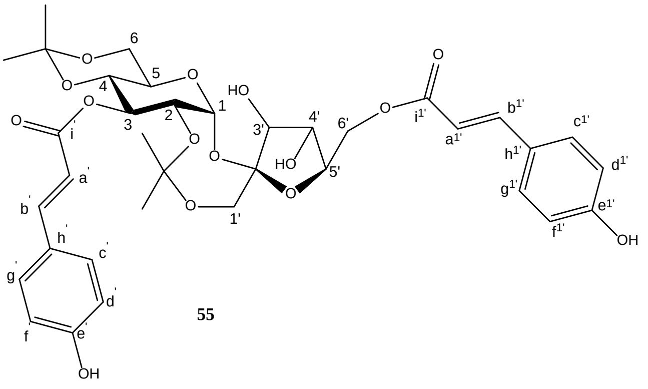

55

Synthesis of 3,6′-di-*O*-feruloyl-2,1′-di-*O*-isopropylidene sucrose, 3,6′-feru 56

Following the general procedure, compound **49** (100 mg, 0.088 mmol) completely reacted with $Mg(OMe)_2$ (134 μL, 0.044 mmol) before quenching the reaction with 1N HCl (88 μL, 0.088 mmol). The product compound **56** (14 mg, 21% yield) was obtained as white solid.

Analytical data for 3,6′-feru 56: mp 140-142 °C; ^{1}H NMR (300 MHz, Acetone-d6): isopropylidene rings: δ 1.24-1.46 (m, 12H, 2 x $(CH_3)_2C$); methoxy and sucrose unit: δ 3.93-3.94 (m, 6H, 3 x H-h′, 3 x H′h$^{1'}$), 3.43-3.48 (m, 1H, H-5), 3.65-4.28 (m, 7H, 2 x H-1′, H-2, H-4, 2 x H-6, H-3′), 4.47-4.50 (m, 3H, H-4′, H-5′, H-6′), .4.72-4.77 (m, 1H, H-6′) 5.31 (t, 1H, *J* = 19 Hz, H-3), 6.10 (d, 1H, *J* = 4 Hz, H-1′); aromatic and alkenyl protons: δ 6.44 (dd, 2H, *J* = 30 Hz, H-a′, H-a$^{1'}$), 6.98 (dd, 2H, *J* = 10.5 Hz, H-d′, H-d$^{1'}$), 7.20 (dd, 2H, *J* = 15 Hz, H-c′, H-c$^{1'}$), 7.35 (d, 2H, *J* = 14 Hz, H-g′, H-g$^{1'}$), 7.61 (dd, 2H, *J* = 25 Hz, H- b′, H-b$^{1'}$) ; ^{13}C NMR (75.5 MHz, Acetone-d6): δ 19.3, 20.8, 20.9, 24.5 (4 x $(CH_3)_2C$), 55.0, 56.4 (2 x OCH_3), 62.8 (C-6), 64.2 (C-5), 66.8 (C-6′), 67.2 (C-1′), 71.5 (C-3), 72.7 (C-2), 72.9 (C-4), 78.1 (C-4′), 80.4 (C-3′), 81.1 (C-5′), 92.0 (C-1), 100.1, 101.9 (2 x $(CH_3)_2C$), 105.1 (C-2′), 111.2 (C-g′, C-g$^{1'}$), 115.7 (C-a′, C-a$^{1'}$), 116.0, 116.1 (C-d′, C-d$^{1'}$), 124.0, 124.2 (C-c′, C-c$^{1'}$), 127.5 (C-i′, C-i$^{1'}$), 145.6, 145.9 (C-b′, C-b$^{1'}$), 148.8 (C-f′, C-f$^{1'}$), 150.1, 150.2 (C-e′, C-e$^{1'}$), 166.7, 167.4 (C-j′, C-j$^{1'}$); HRMS (ESI positive mode): found *m/z* 797.2598 $[M+Na]^+$, calcd.797.2633 for $C_{38}H_{46}O_{17}Na$.

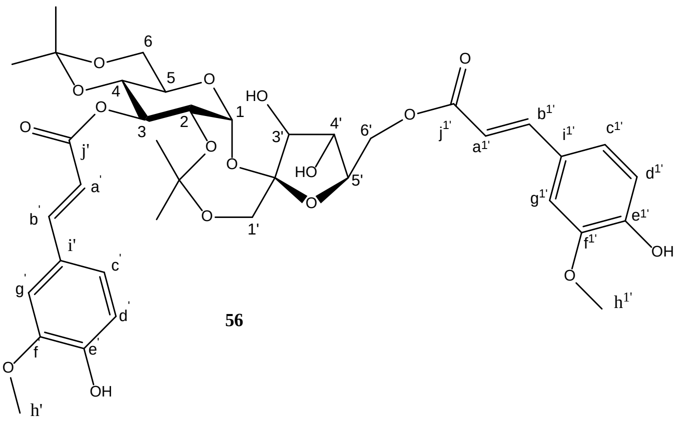

56

Synthesis of 3,6′-di-*O*-(3,4-dimethoxycinanamoyl)-2,1′-di-*O*-isopropylidene sucrose, 3,6′-diOMe 59

Following the general procedure, compound **52** (100 mg, 0.092 mmol) completely reacted with $Mg(OMe)_2$ (140 μL, 0.046 mmol) before quenching the reaction with 1N HCl (92 μL, 0.092 mmol). The product compound **59** (66 mg, 90% yield) was obtained as white solid.

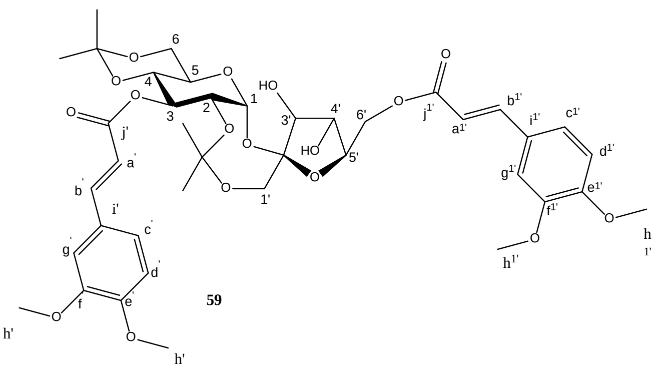

59

Analytical data for 3,6′-diOMe 59: mp 132-134 °C; ^{1}H NMR (300 MHz, Acetone-d6): isopropylidene rings: δ 1.27-1.44 (m, 12H, 2 x $(CH_3)_2C$); methoxy and sucrose unit: δ 3.87-3.88 (m, 12H, 6 x H-h′, 6 x H-h$^{1'}$) ,3.48-3.51 (m, 1H, H-5), 3.70-4.18 (m, 7H, 2 x H-1′, H-2, H-4, 2 x H-6, H-3′), 4.18-4.32 (m, 3H, H-4′, H-5′, H-6′) , 4.48-4.52 (m, 1H, H-6′), 5.40 (t, 1H, *J* = 18.5 Hz, H-3), 6.16 (d, 1H, *J* = 4 Hz, H-1′); aromatic and alkenyl protons: δ 6.29 (dd, 2H, *J* = 30 Hz, H-a′, H-a$^{1'}$) 6.81 (dd, 2H, *J* = 11 Hz, H-d′, H-d$^{1'}$), 7.02 (dd, 2H, *J* = 18 Hz, H-c′, H-c$^{1'}$), 7.07 (d, 2H, *J* = 16 Hz, H-g′, H-g$^{1'}$), 7.60 (dd, 2H, *J* = 24 Hz, H-b′, H-b$^{1'}$) ; ^{13}C NMR (75.5 MHz, Acetone-d6): δ 19.3, 24.5, 25.6, 26.2 (4 x $(CH_3)_2C$), 56.1, 56.2 (4 x OCH_3), 62.8 (C-6), 64.2 (C-5), 66.8 (C-6′), 67.2 (C-1′), 71.6 (C-3), 72.7 (C-2), 72.9 (C-4), 78.0 (C-4′), 80.4 (C-3′), 81.1 (C-5′), 92.0 (C-1), 100.1, 101.9 (2 x $(CH_3)_2C$), 105.1 (C-2′), 111.0, 112.3 (C-c′, C-c$^{1'}$, C-f′, C-f$^{1'}$), 116.3, 116.6 (C-a′, C-a$^{1'}$), 123.7, 123.8 (C-g′, C-g$^{1'}$), 128.3 (C-i′, C-i$^{1'}$), 145.4, 145.7(C-b′, C-b$^{1'}$), 150.7 (C-e′, C-e$^{1'}$), 152.6 (C-d′, C-d$^{1'}$), 166.6, 167.4 (C-j′, C-j$^{1'}$); HRMS (ESI positive mode): found *m/z* 803.3154 $[M+H]^+$, calcd.803.3126 for $C_{40}H_{51}O_{17}$.

Synthesis of 3,6′-di-*O*-(3,4,5-trimethoxycinanamoyl)-2,1′-di-*O*-isopropylidene sucrose, 3,6′-triOMe 60

Following the general procedure, compound **53** (100 mg, 0.087 mmol) completely reacted with $Mg(OMe)_2$ (134 μL, 0.044 mmol) before quenching the reaction with 1N HCl (87 μL, 0.087 mmol). The product compound **60** (47 mg, 63% yield) was obtained as white solid.

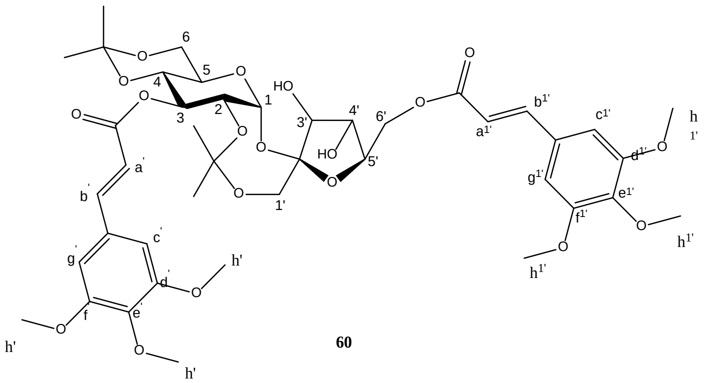

60

Analytical data for 3,6′-triOMe 60: mp 125-127 °C; ^{1}H NMR (300 MHz, $CDCl_3$): isopropylidene rings: δ 1.23-1.45 (m, 12H, 2 x $(CH_3)_2C$); methoxy and sucrose unit: δ 3.85-3.89 (m, 18H, 9 x H-h′, 9 x H-h$^{1'}$), 3.49- 3.53 (m, 1H, H-5), 3.71-4.11 (m, 7H, 2 x H-1′, H-2, H-4, 2 x H-6, H-3′), 4.17-4.52 (m, 3H, H-4′, H-5′, H-6′), 4.50-4.54 (m, 1H, H-6′) 5.34 (t, 1H, *J* = 19 Hz, H-3), 6.17 (d, 1H, *J* = 4 Hz, H-1′); aromatic and alkenyl protons: δ 6.35 (dd, 2H, *J* = 31 Hz, H-a′, H-a$^{1'}$), 6.74 (d, 4H, *J* = 6 Hz, H-c′, H-c$^{1'}$ H-g′, H-g$^{1'}$), 7.58 (dd, 2H, *J* = 19 Hz, H-b′, H-b$^{1'}$) ; ^{13}C NMR (75.5 MHz, $CDCl_3$): δ 19.2, 21.3, 24.3, 25.5 (4 x $(CH_3)_2C$), 56.4, 60.6 (6 x OCH_3), 61.2 (C-6, C-5), 66.0 (C-6′, C-1′), 71.3 (C-3), 71.8 (C-4), 72.0 (C-2), 77.4 (C-4′), 79.9 (C-3′), 80.3 (C-5′), 91.5 (C-1), 100.1, 101.8 (2 x $(CH_3)_2C$), 104.3 (C-2′), 105.5, 105.6 (C-c′, C-c$^{1'}$, C-g′, C-g$^{1'}$), 117.2 (C-a′, C-a$^{1'}$), 130.0, 130.1 (C-i′, C-i$^{1'}$), 140.4, 140.5 (C-e′, C-e$^{1'}$), 145.4 (C-b′, C-b$^{1'}$), 153.6, 153.7 (C-d′, C-d$^{1'}$, C-f′, C-f$^{1'}$), 166.9, 167.0 (C-j′, C-j$^{1'}$); HRMS (ESI positive mode): found *m/z* 885.3162 $[M+Na]^+$, calcd.885.3157 for $C_{42}H_{54}O_{19}Na$.

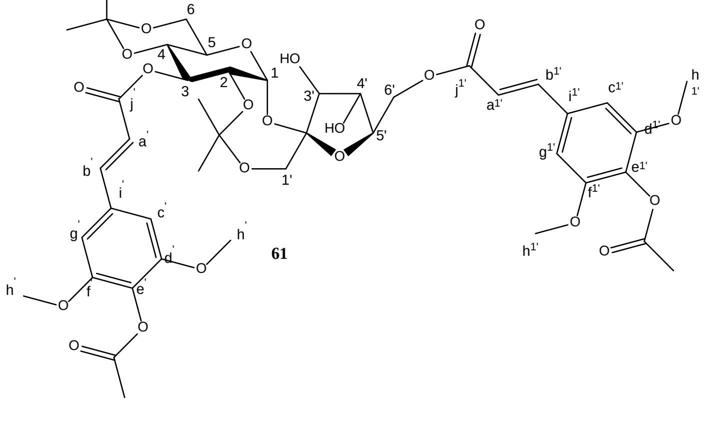

61

Synthesis of 3,6′-di-*O*-acetoxy-sinapoyl-2,1′-di-*O*-isopropylidene sucrose, 3,6′-OAc-sinap 61

Following the general procedure, compound **50** (100 mg, 0.083 mmol) completely reacted with $Mg(OMe)_2$ (128 μL, 0.042 mmol) before quenching the reaction with 1N HCl (83 μL, 0.083 mmol). The product compound **61** (40 mg, 53% yield) was obtained as white solid.

Analytical data for 3,6′-OAc-sinap 61: mp 136-138 °C; isopropylidene rings: δ 1.25-1.43 (m, 12H, 2 x $(CH_3)_2C$); OAc-sinap acetoxy and methoxy sucrose unit: δ2.27-2.30 (m, 7H, 3 x H-I′, 3 x H-i$^{1'}$), 3.79-3.80 (m, 12H, 6 x H-h′, 6 x H-h$^{1'}$), 3.47-4.03 (m, 8H), 4.18-4.54

(m, 3H), 5.25 (m, 1H, H-6′), 5.42 (t, 1H, *J* = 19 Hz, H-3), 6.15 (d, 1H, *J* = 4 Hz, H-1′); aromatic and alkenyl protons: δ 6.37 (dd, 2H, *J* = 34 Hz, H-a′, H-a$^{1'}$), 6.74 (d, 4H, *J* = 7 Hz, H-c′, H-c$^{1'}$, H-g′, H-g$^{1'}$), 7.58 (dd, 2H, *J* = 16 Hz, H-b′, H-b$^{1'}$); ^{13}C NMR (75.5 MHz, $CDCl_3$): δ 19.2, 20.6, 24.2, 25.3, 29.1, 31.0, 53.6, 56.3, 62.3, 63.9, 65.9, 66.5, 68.3, 71.6, 72.1, 79.6, 80.3, 91.2, 99.9, 100.0, 100.1, 101.5, 101.6, 104.3, 104.4, 104.8, 104.9, 118.1, 118.4, 123.7, 128.4, 128.7, 130.4, 130.7, 130.8, 132.6, 132.7, 132.8, 132.9, 152.5, 152.6, 166.7, 166.8, 168.6.

Synthesis of 3,6′-di-*O*-(3,4-di-*tert*-butyldimethylsiloxycinnamoyl)-2,1′-di-*O*-isopropylidene sucrose, 3,6′-OTBS-caff 62

Following the general procedure, compound **51** (100 mg, 0.084 mmol) completely reacted with $Mg(OMe)_2$ (128 μL, 0.042 mmol) before quenching the reaction with 1N HCl (84 μL, 0.084 mmol). The product compound **62** (83 mg, 82% yield) was obtained yellow solid.

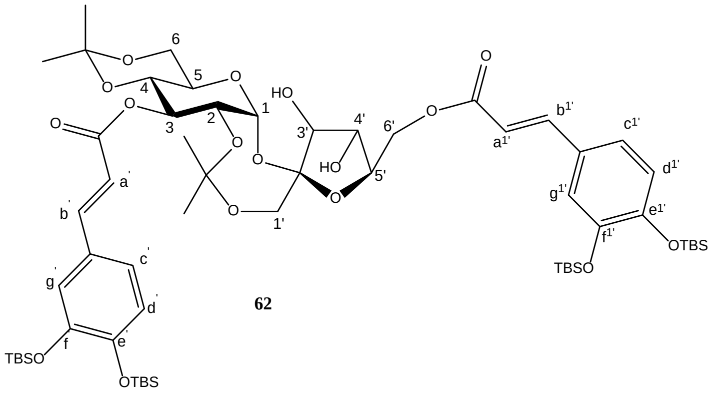

62

Analytical data for 3,6′-OTBS-caff 62: ^{1}H NMR (300 MHz, $CDCl_3$): TBS groups: δ 0.20 (m, 24H), 0.97, 0.98 (m, 36H); isopropylidene rings: δ 1.24-1.45 (m, 12H, 2 x $(CH_3)_2C$); sucrose unit: δ 3.36 (m, 2H), 3.51 (d, 1H, *J* = 12.5 Hz), 3.71-3.78 (m, 4H), 3.88-3.95 (m, 4H), 4.18- 4.22 (m, 3H), 4.27-4.33 (m, 1H), 4.48 (d, 1H, *J* = 11.5 Hz), 5.38 (t, 1H, *J* = 19 Hz, H-3), 6.18 (d, 1H, *J* = 4 Hz, H-1′); aromatic and alkenyl protons: δ 6.26 (dd, 2H, *J* = 30 Hz, H-a′, H-a$^{1'}$), 6.80 (dd, 2H, *J* = 10 Hz, H-d′, H-d$^{1'}$), 6.97-7.04 (m, 4H H-c′, H-c$^{1'}$, H-g′, H-g$^{1'}$), 7.57 (dd, 2H, *J* = 25.5 Hz, H-b′, H-b$^{1'}$) ; ^{13}C NMR (75.5 MHz, Acetone-d6): δ -4.8, -4.7, -1.2, 18.2, 18.4, 23.6, 24.6, 25.3, 25.4, 63.3, 63.8, 70.8, 71.8, 79.5, 91.1, 99.2, 101.0, 104.2, 115.9, 120.6, 120.8, 121.3, 122.3, 126.5, 128.0, 128.4, 128.5, 144.1, 144.4, 147.1, 149.3, 165.6, 166.4.

Synthesis of 3,6′-di-*O*--sinapoyl-2,1′-di-*O*-isopropylidene sucrose, 3,6′-sinap 57

A mixture of compound **61** (100 mg, 0.108 mmol) and piperidine (42.7 μL, 0.432 mmol) in EtOH/THF (1 ml, 5:1) was stirred for 12 hours at room temperature. Upon reaction completion (TLC), the crude mixture was subjected to purification using silica gel column chromatography using EtOAc/Hexane (3:2) using as the eluent. Compound **57** (41 mg, 45 % yield) was obtained as white solid.

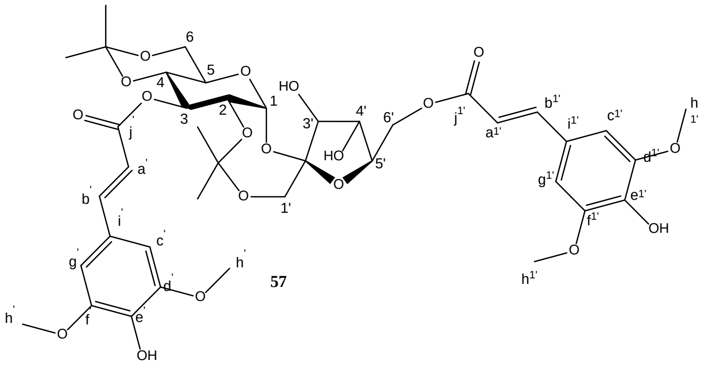

57

Analytical data for 3,6′-sinap 57: mp 136-138 °C; ^{1}H NMR (300 MHz, $CDCl_3$): δ isopropylidene rings: δ 1.23-1.43 (m, 12H, 2 x $(CH_3)_2C$); sinapoyl methoxy and sucrose unit: δ 3.85-3.86 (m, 14H, 6 x H-h′, 6 x H-h$^{1'}$), 3.32-3.71 (m, 8H, H-5, 2 x H-1′, H-2, H-4, 2 x H-6, H-3′), 3.94-4.54 (m, 4H, H-4′, H-5′, 2 x H-6′), 5.41(t, 1H, *J* = 19 Hz, H-3), 6.16 (d, 1H, *J* = 4 Hz, H-1′); aromatic and alkenyl protons: δ 6.27 (dd, 2H, *J* = 30 Hz, H-a′, H-a$^{1'}$), 6.75 (d, 4H, *J* = 7.5 Hz, H-c′, H-c$^{1'}$, H-g′, H-g$^{1'}$), 7.54 (dd, 2H, *J* = 18 Hz, H-b′, H-b$^{1'}$); ^{13}C NMR (75.5 MHz, $CDCl_3$): δ 19.7, 24.9, 26.0 (4 x $(CH_3)_2C$), 57.1 (4 x OCH_3), 63.2 (C-6), 64.6 (C-5), 67.2 (C-6′), 67.6 (C-1′), 71.9 (C-3), 73.2 (C-2), 73.3 (C-4), 78.4 (C-4′), 80.9 (C-3′), 81.5 (C-5′), 92.4 (C-1), 100.5, 102.3 (2 x $(CH_3)_2C$), 105.5 (C-2′), 107.3 (C-c′, C-c$^{1'}$, C-g′, C-g$^{1'}$), 116.4, 116.7 (C-a′, C-a$^{1'}$), 126.6 (C-i′, C-i$^{1'}$), 139.9 (C-e′, C-e$^{1'}$), 146.4, 146.7 (C-b′, C-b$^{1'}$), 149.4 (C-d′, C-d$^{1'}$, C-f′, C-f$^{1'}$), 167.1, 167.9 (C-j′, C-j$^{1'}$); HRMS (ESI positive mode): found *m/z* 857.2817 $[M+Na]^+$, calcd.857.2844 for $C_{40}H_{50}O_{19}Na$.

Synthesis of 3,6′-di-*O*-caffeoyl-2,1′-di-*O*-isopropylidene sucrose, 3,6′-caff 58

To a stirred solution of 3,6′-OTBS-caff **62** (100 mg, 0.083 mmol) in pyridine (10.0 ml) was added NEt_3 (24 μL, 0.17 mmol) and 1.56 M $3HF{\cdot}NEt_3$ (161 μL, 0.25 mmol) and the reaction mixture was left to stir for 12 hours at room temperature (TLC). Upon reaction completion, pyridine was removed under reduced pressure and the crude product was then subjected to silica gel column chromatography purification using EtOAc/Hexane (3:2) as the eluent. 3,6′-Caff **58** (58 mg, 94% yield) was obtained as yellow solid.

Analytical data for 3,6′-caff 58: mp 110-112 °C; ^{1}H NMR (300 MHz, $CDCl_3$): isopropylidene rings: δ 1.23-1.46 (m, 12H, 2 x $(CH_3)_2C$); sucrose unit: δ 3.45-3.49 (m, 1H, H-5), 3.68-3.97 (m, 7H, 2 x H-1′, H-2, H-4, 2 x H-6, H-3′), 4.05-4.52 (m, 3H, H-4′, H-5′, 2 x H-6′), 4.72-4.76 (m, 1H, H-6′), 5.30 (t, 1H, *J* = 19 Hz, H-3), 6.11 (d, 1H, *J* = 4 Hz, H-1′); aromatic and alkenyl protons: δ 6.29 (dd, 2H, *J* = 30 Hz, H-a′, H-a1′), 6.88 (dd, 2H, *J* = 10 Hz, H-d′, H-d1′), 7.06-7.07 (m, 2H, H-c′, H-c1′), 7.17 (dd, 2H, *J* = 10 Hz, H-g′, H-g1′), 7.57 (dd, 2H, *J* = 26 Hz, H-b′, H-b1′); ^{13}C NMR (75.5 MHz, Acetone-d6): δ 18.4, 19.6, 23.6, 24.6 (4 x $(CH_3)_2C$), 61.9 (C-6), 63.3 (C-5), 65.8 (C-6′), 66.3 (C-1′), 70.7 (C-3), 71.8 (C-4), 72.0 (C-2), 77.2 (C-4′), 79.5 (C-3′), 80.2 (C-5′), 91.1 (C-1), 99.2, 101.0 (2 x $(CH_3)_2C$), 104.2 (C-2′), 114.2, 114.3 (C-a′, C-a1′), 114.6, 115.0 (C-c′, C-c1′, C-f′, C-f1′), 121.6, 121.7 (C-g′, C-g1′), 126.8 (C-h′, C-h1′), 144.9, 145.2, (C-b′, C-b1′), 145.7, (C-d′, C-d1′), 148.4, 148.5 (C-e′, C-e1′), 165.8, 166.5 (C-i′, C-i1′); HRMS (ESI positive mode): found *m/z* 769.2323 $[M+Na]^+$, calcd.769.2320 for $C_{36}H_{42}O_{17}Na$.

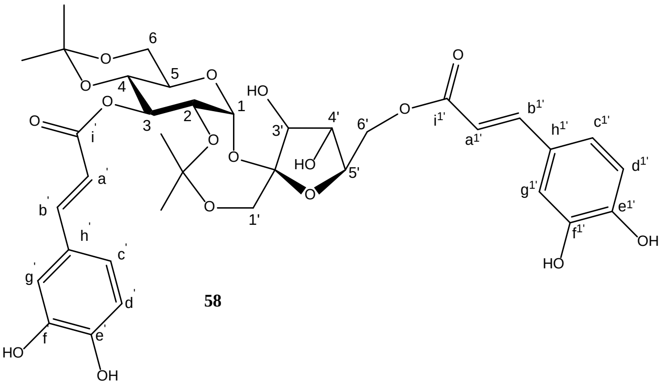

58

The synthesis of the below protected cinnamic acids was accomplished according to the literature as shown in the below figure.

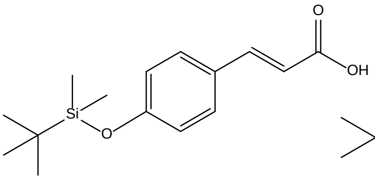

OTBS-coum acid
4-tert-Butyldimethylsiloxycinnamic acid (Ref 3)

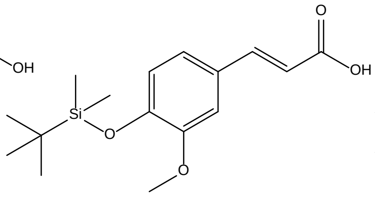

OTBS-feru acid
4-tert-butyldimethylsilyloxy-3-methoxycinnamic acid (Ref 3)

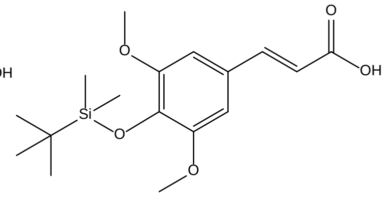

OTBS-sinap acid
4- tert-butyldimethylsilyloxy-3,5-dimethoxycinnamic acid (Ref 3)

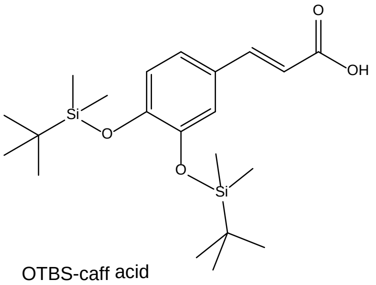

OTBS-caff acid
3,4-di-tert-butyldimethylsiloxycinnamic acid (Ref 3)

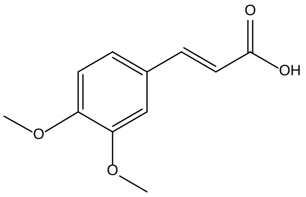

diOMecinn acid
3,4-dimethoxycinnamic acid (Ref 3)

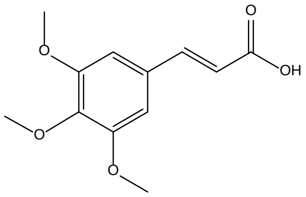

triOMecinn acid
3,4,5-trimethoxycinnamic acid (Ref 3)

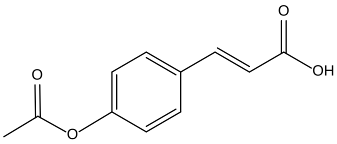

OAc-coum acid
4-acetoxycinnamic acid (Ref 3, 8)

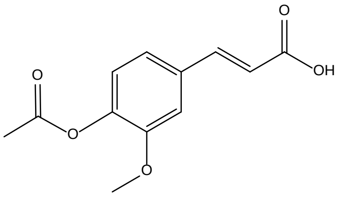

OAc-feru acid
4-acetoxy-3-methoxycinnamic acid (Ref 8, 9)

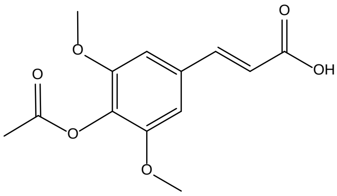

OAc-sinap acid
4-acetoxy-3,5-dimethoxycinnamic acid (Ref 8, 9)

NMR spectra of compounds 3, 25, 26, 45, 11-17, 33-38, 18-24, 23, 43, and 59

Spectra of compound **3**

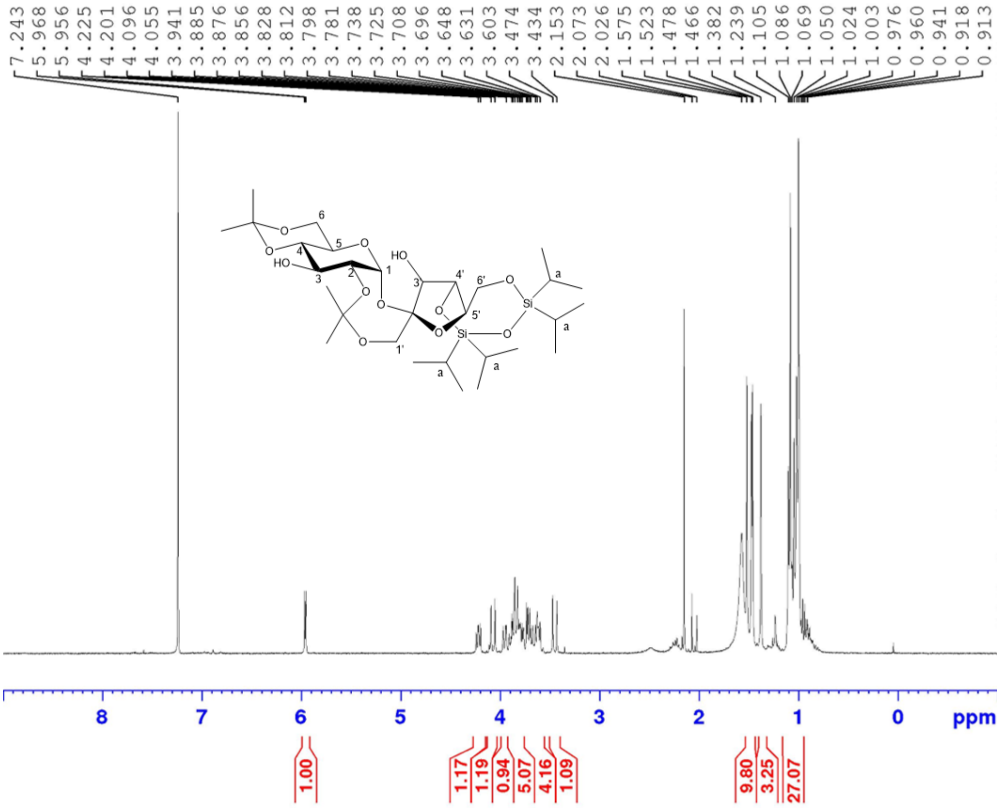

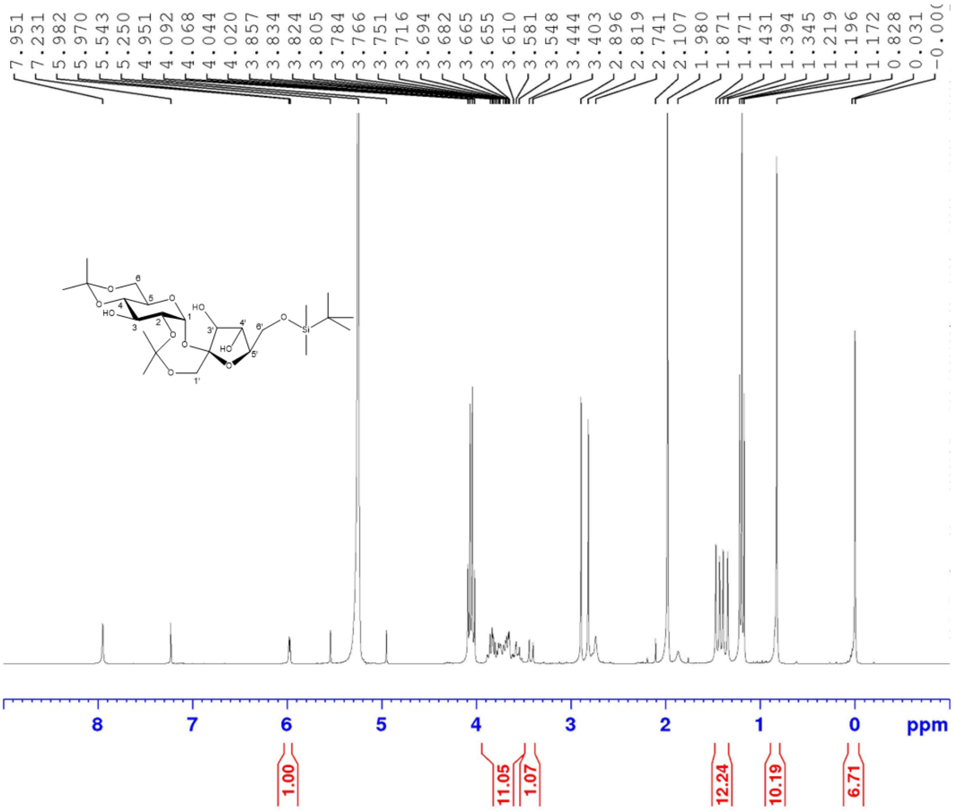

Spectra of compound **25**

Spectra of compound **26**

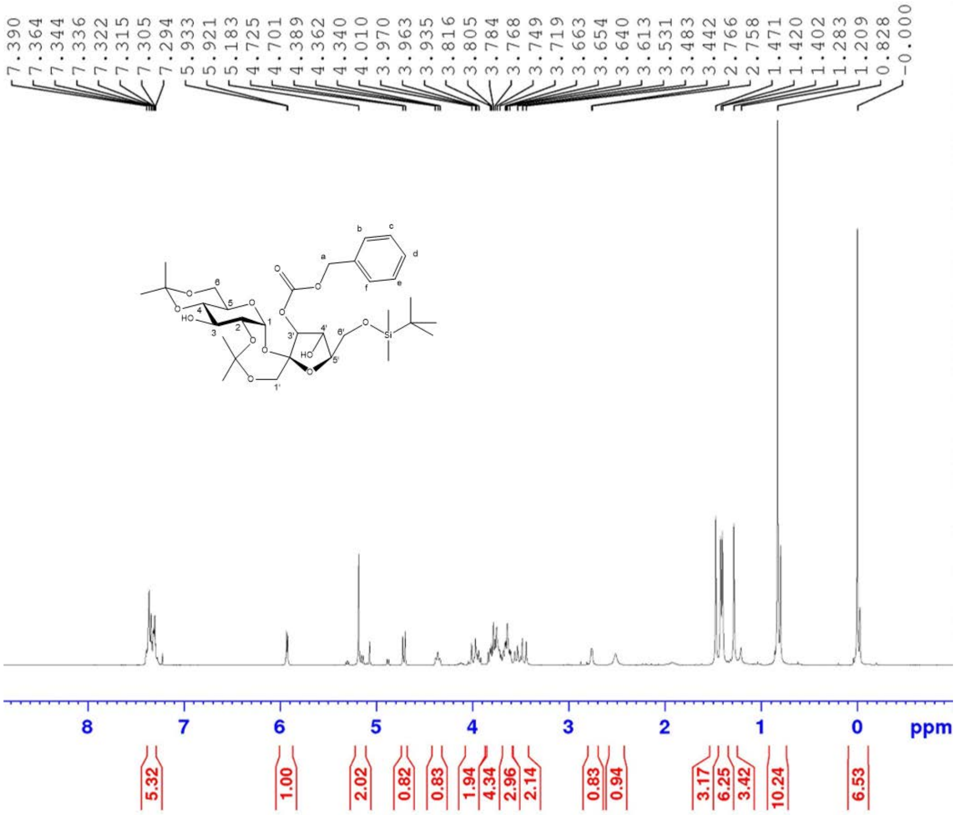

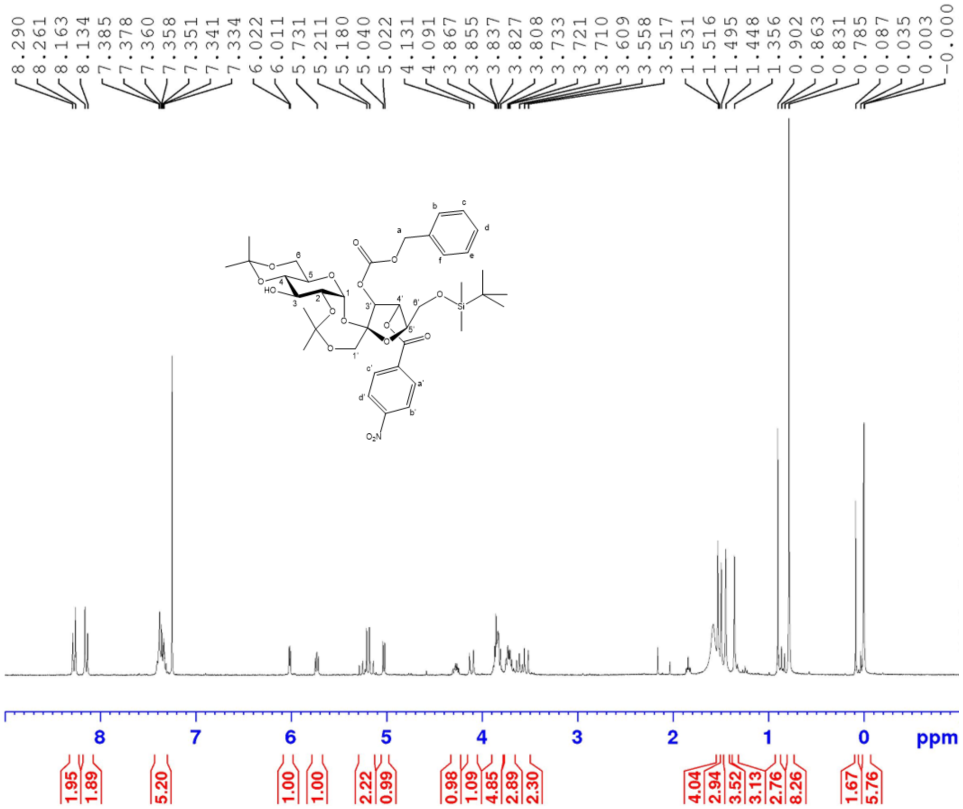

Spectra of compound **45**

Spectra of compound **11**

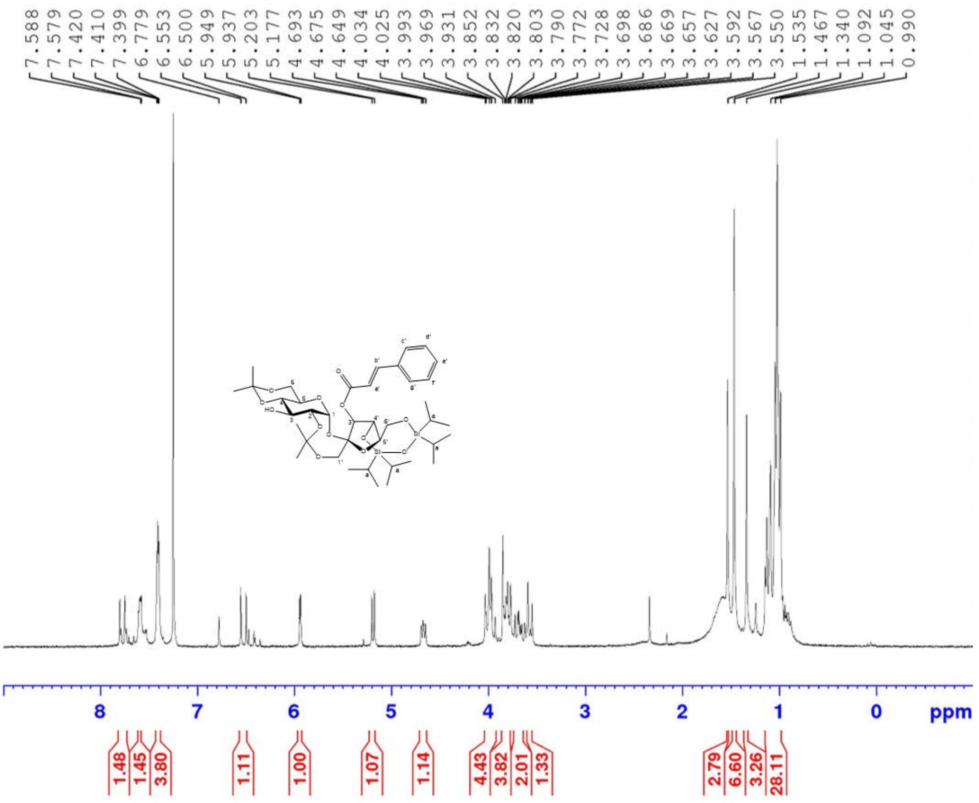

Spectra of compound **12**

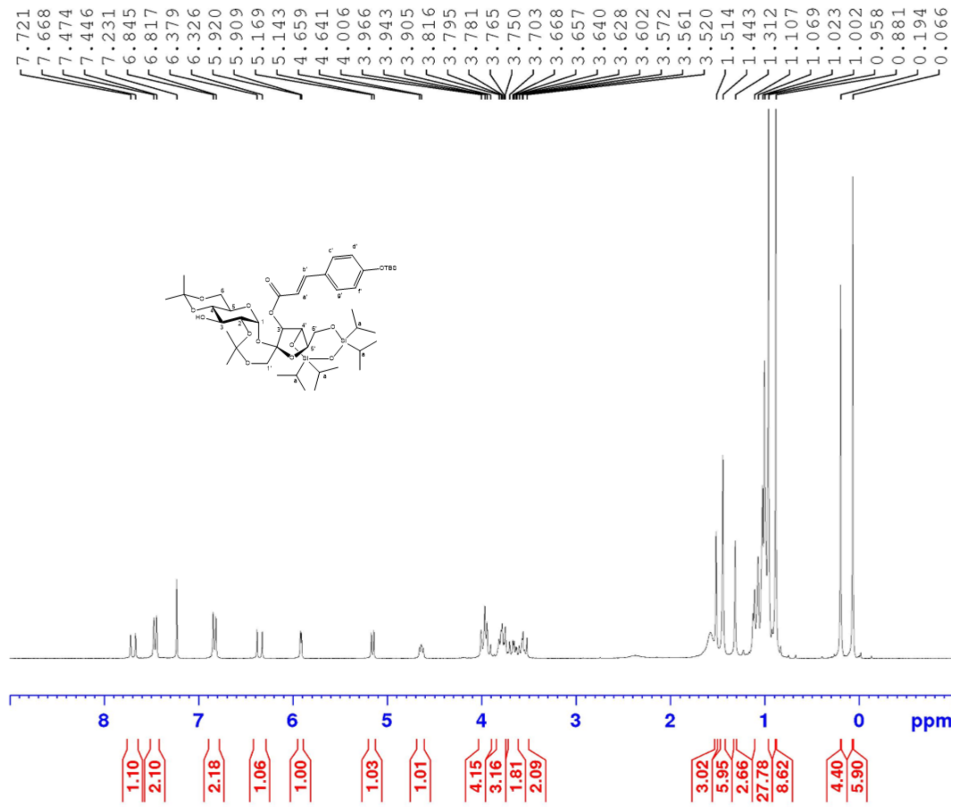

Spectra of compound **13**

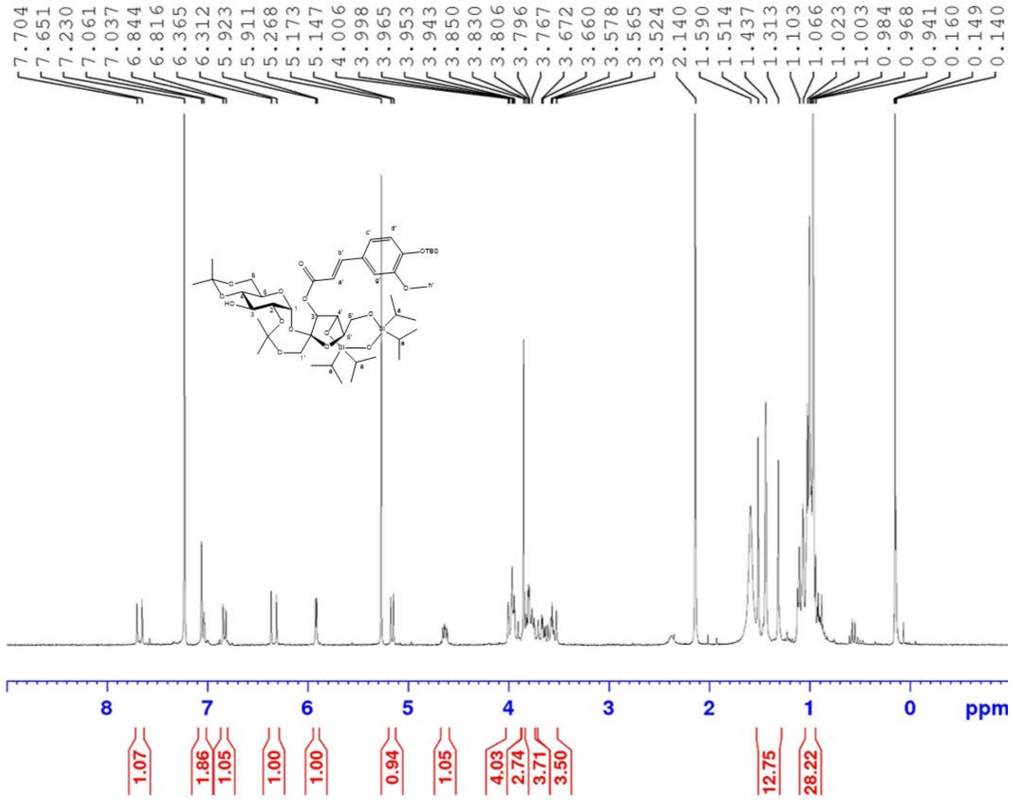

Spectra of compound **14**

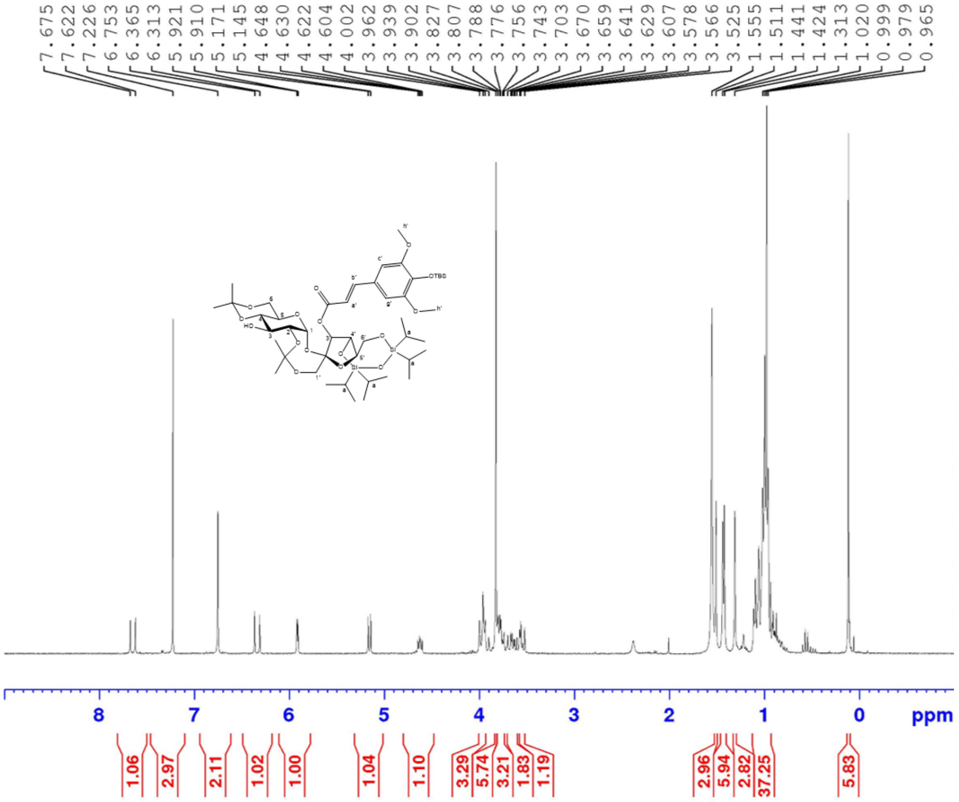

Spectra of compound **15**

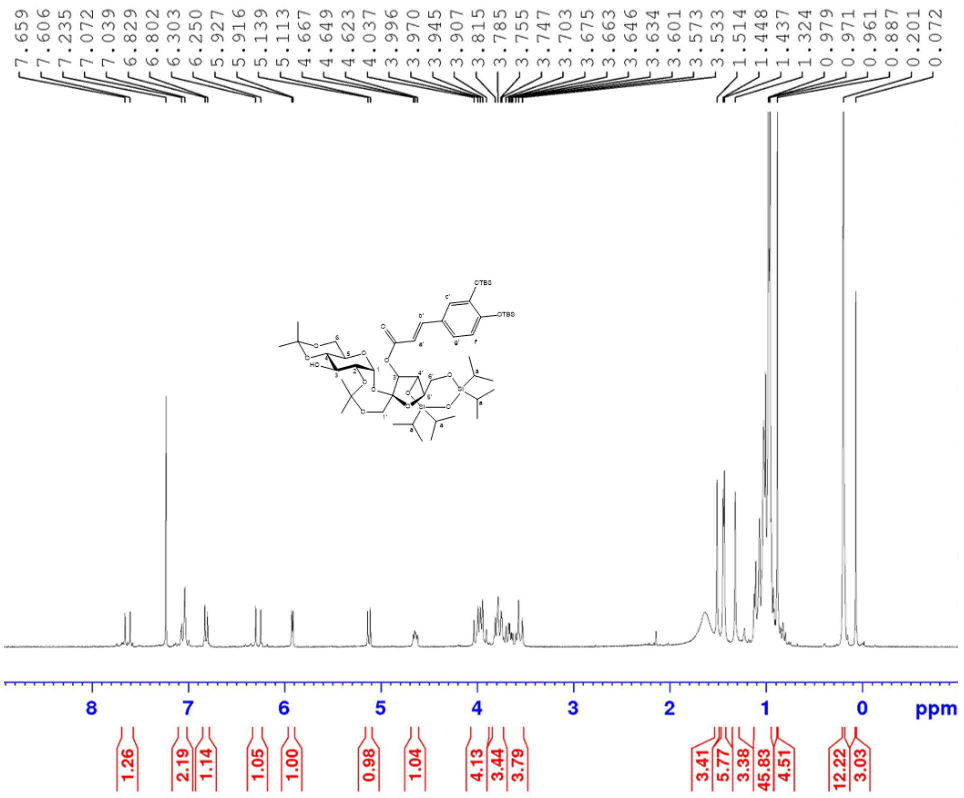

Spectra of compound **16**

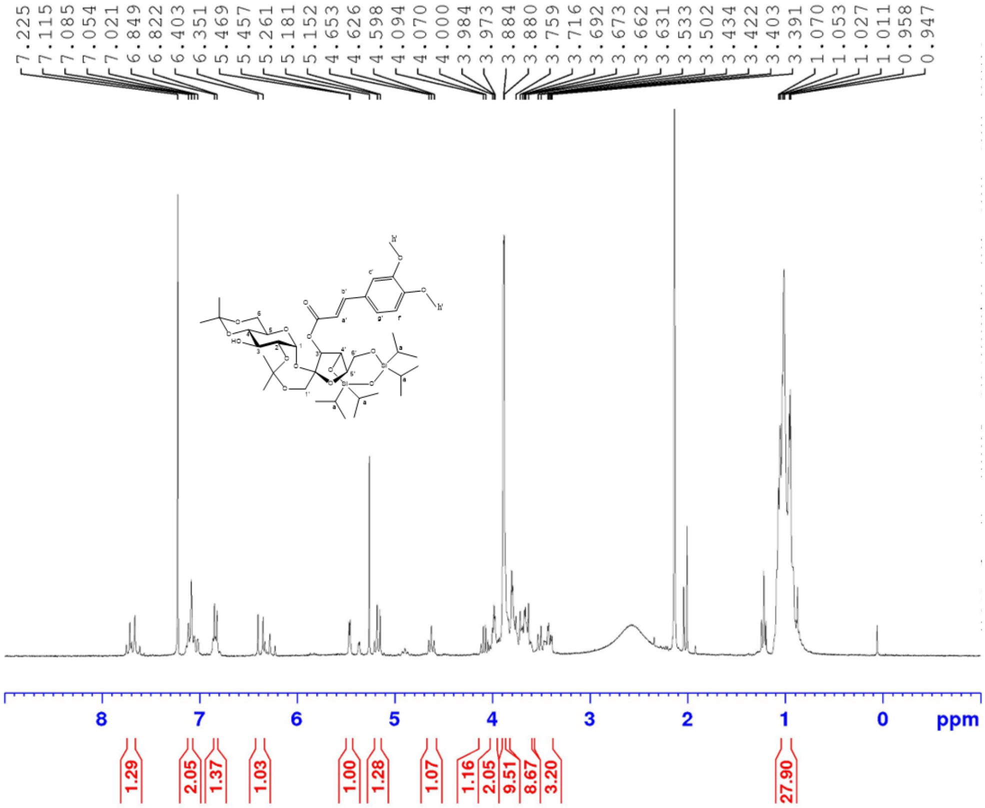

Spectra of compound **17**

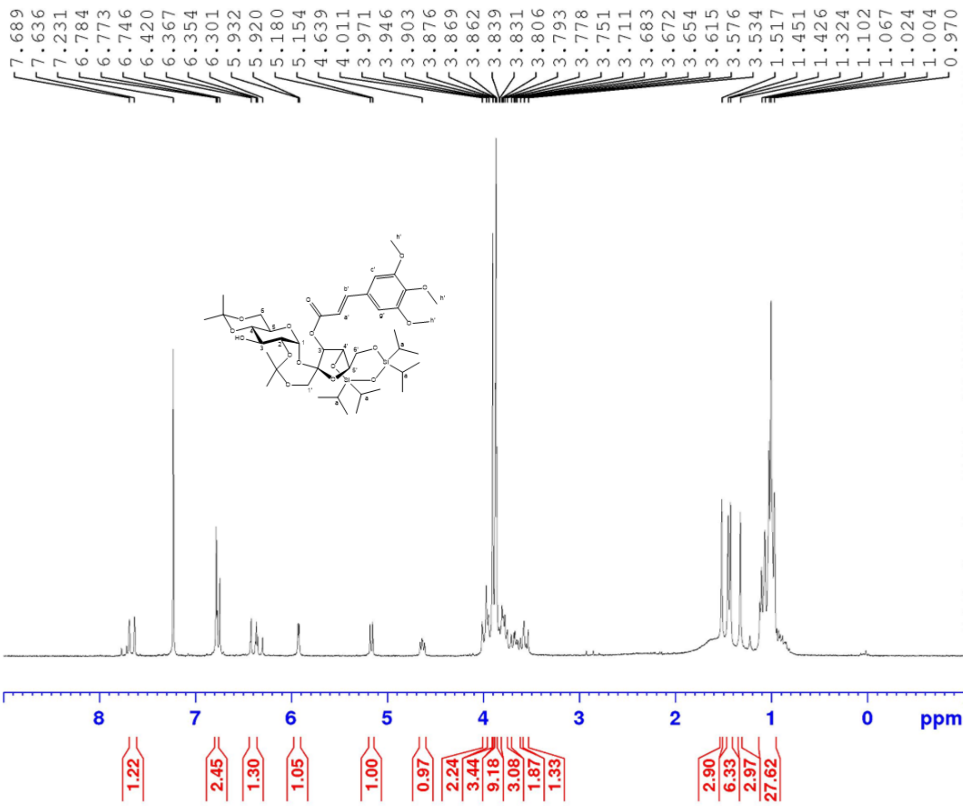

Spectra of compound **33**

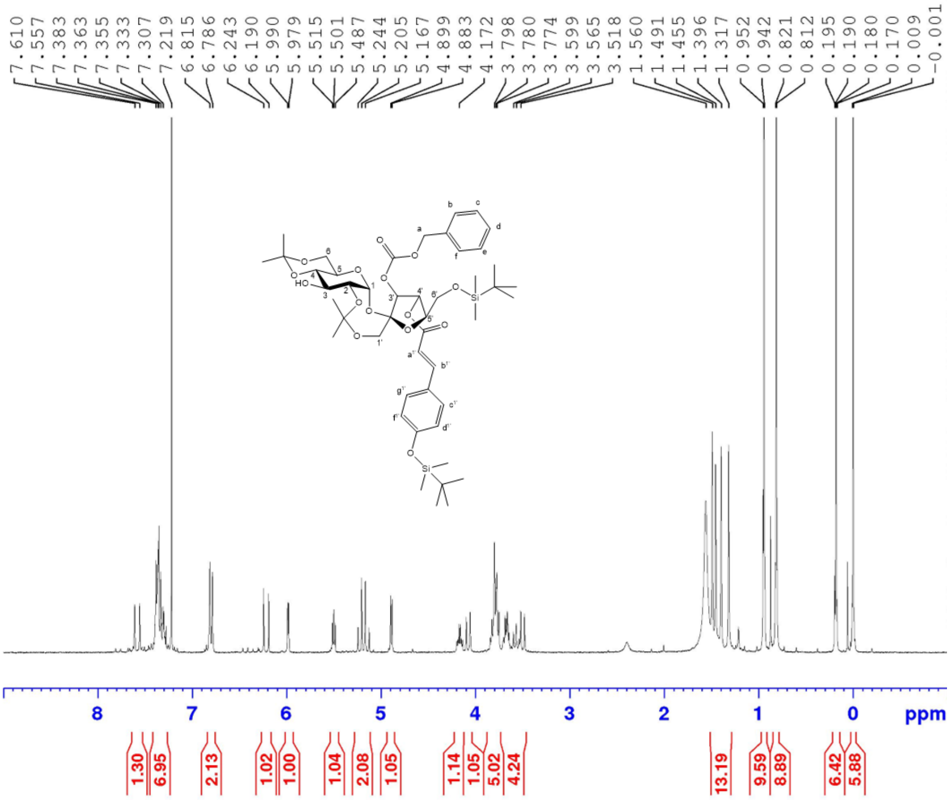

Spectra of compound **34**

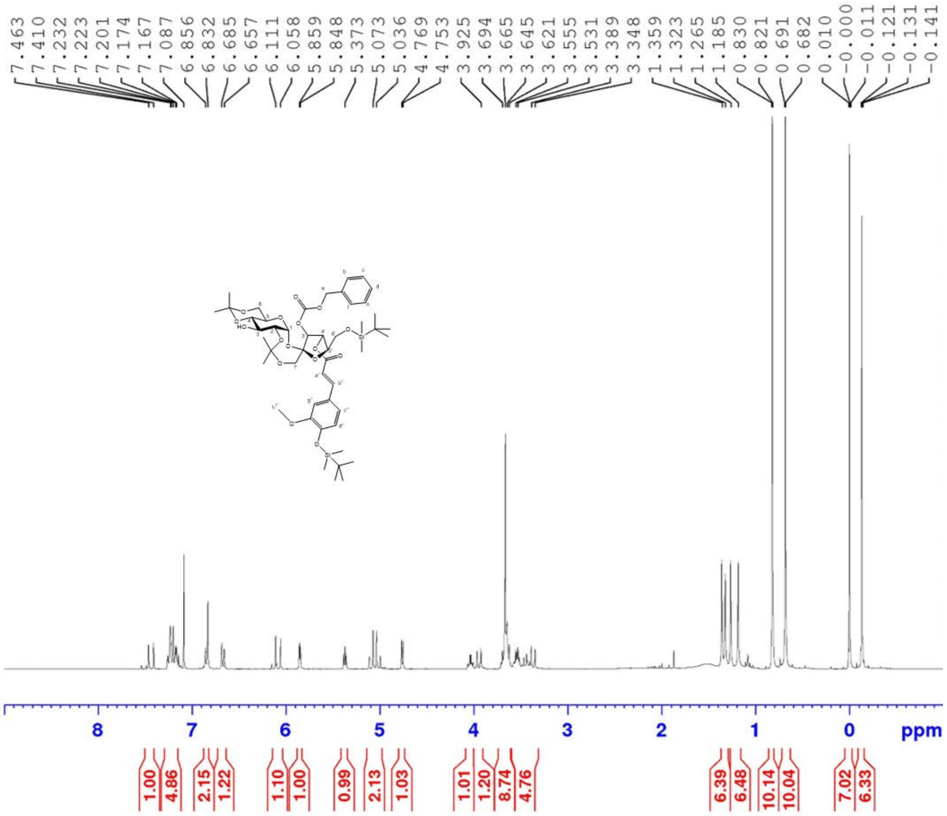

Spectra of compound **35**

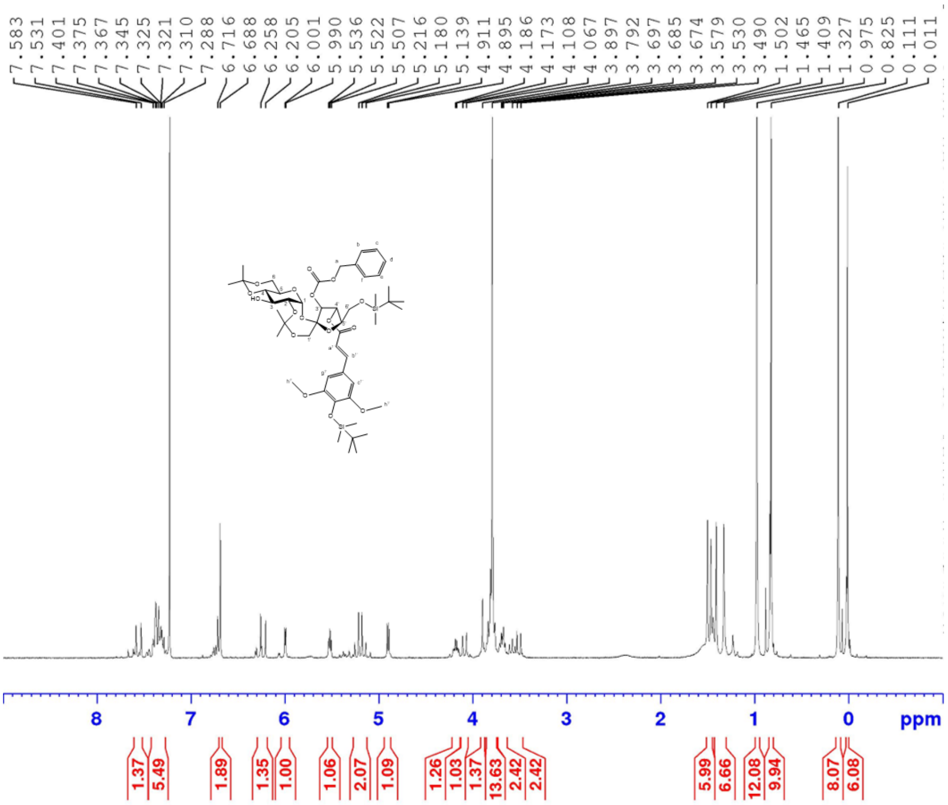

Spectra of compound **36**

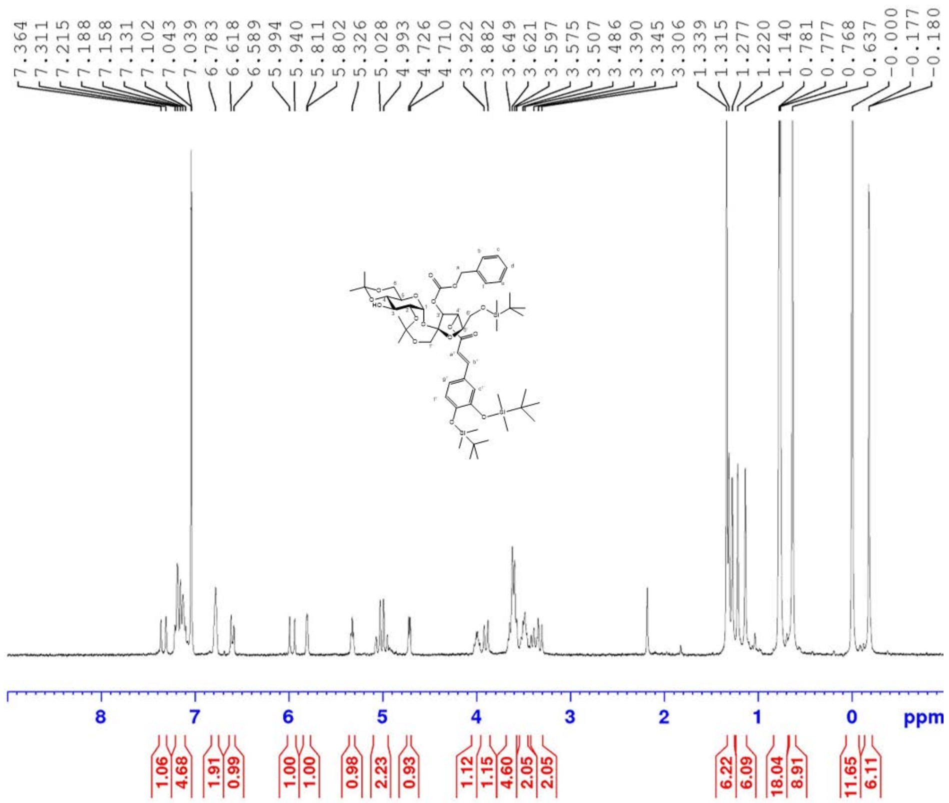

Spectra of compound **37**

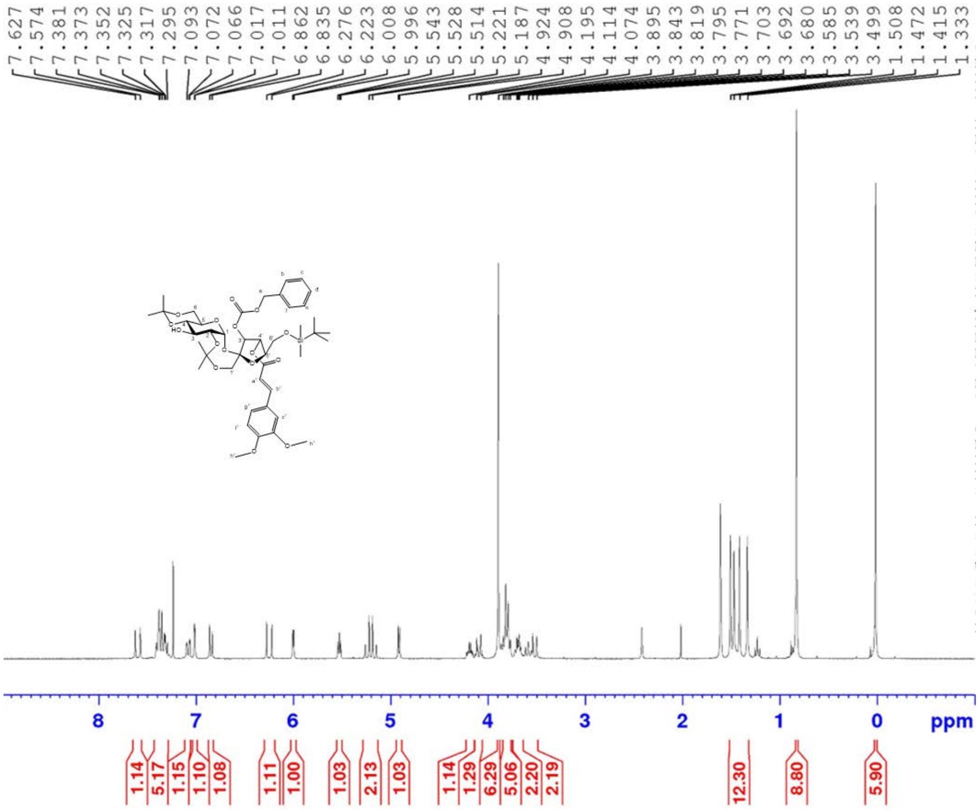

Spectra of compound **38**

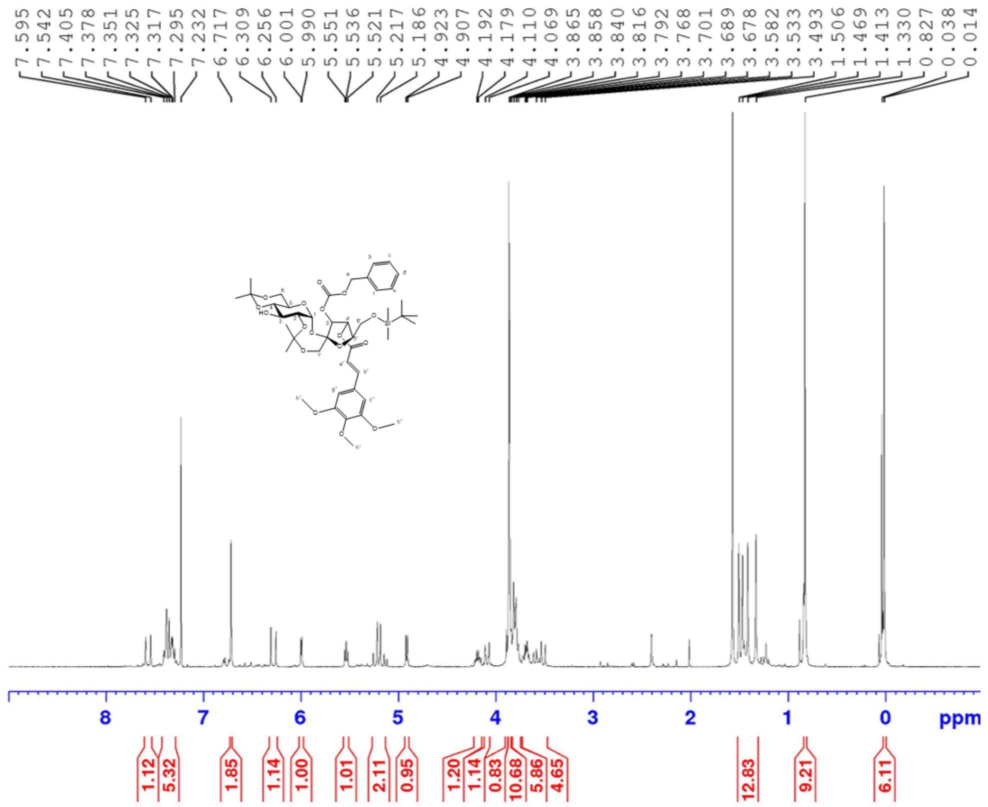

COSY spectra of **18**

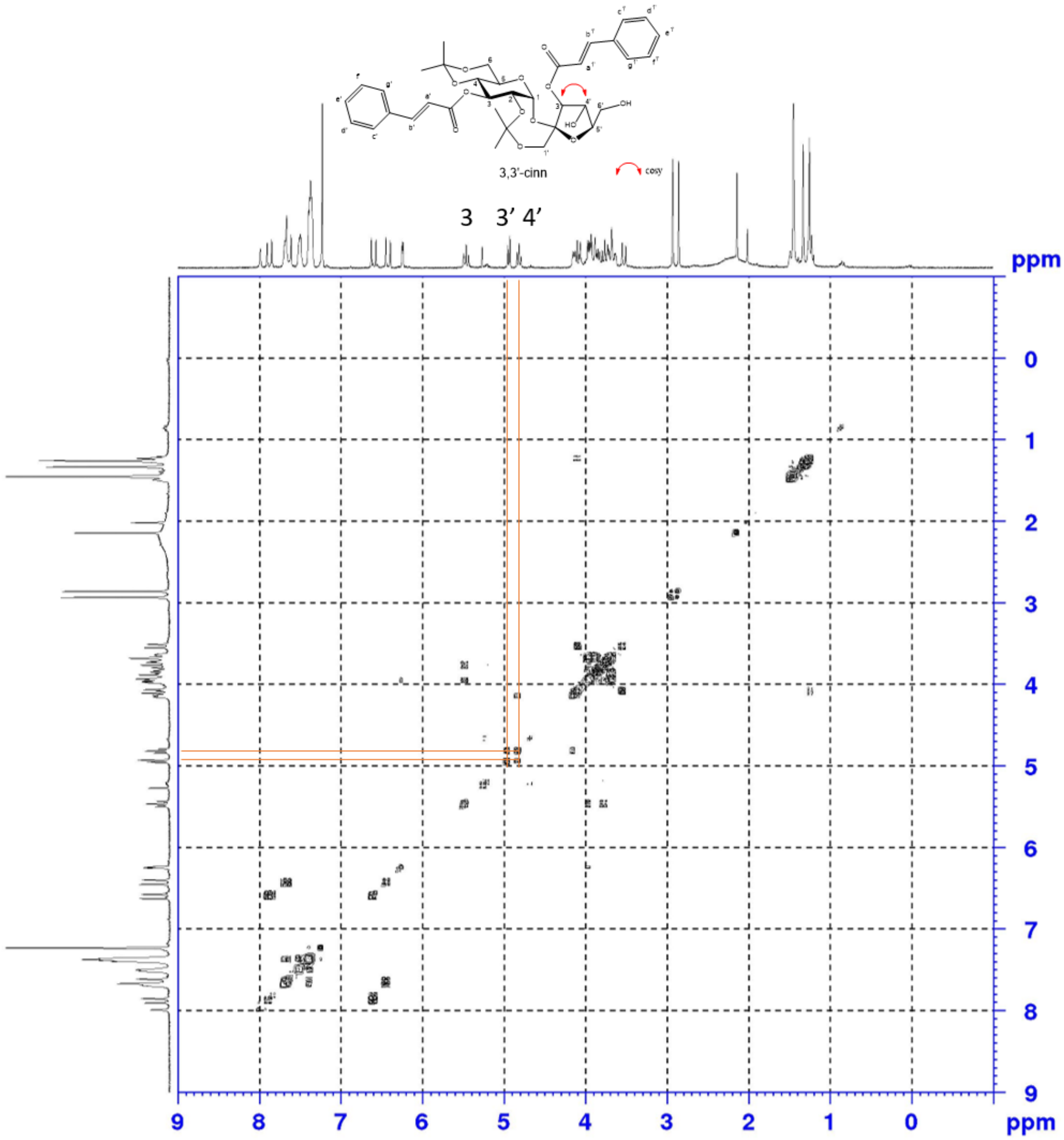

COSY spectra of **19**

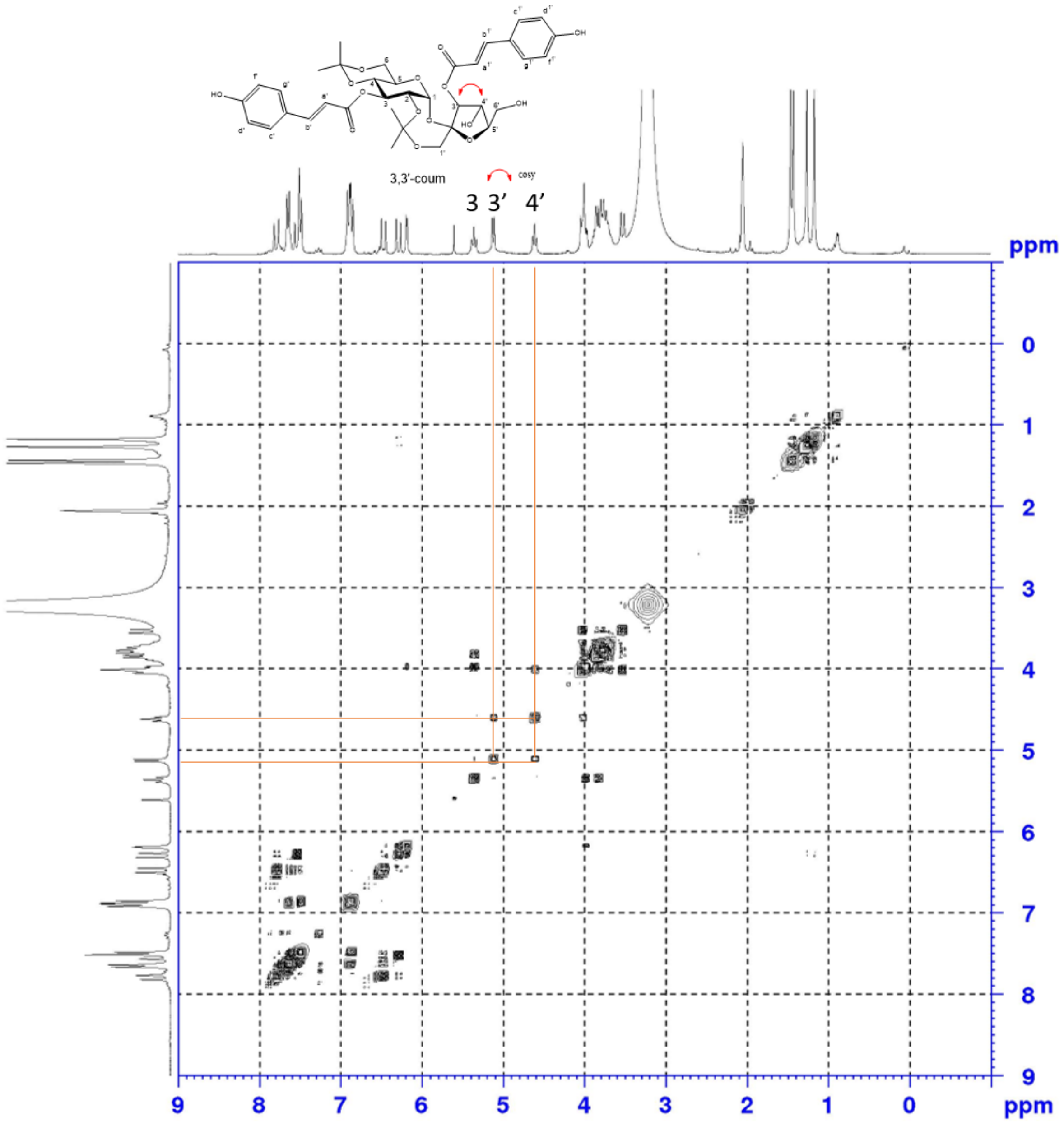

COSY spectra of **20**

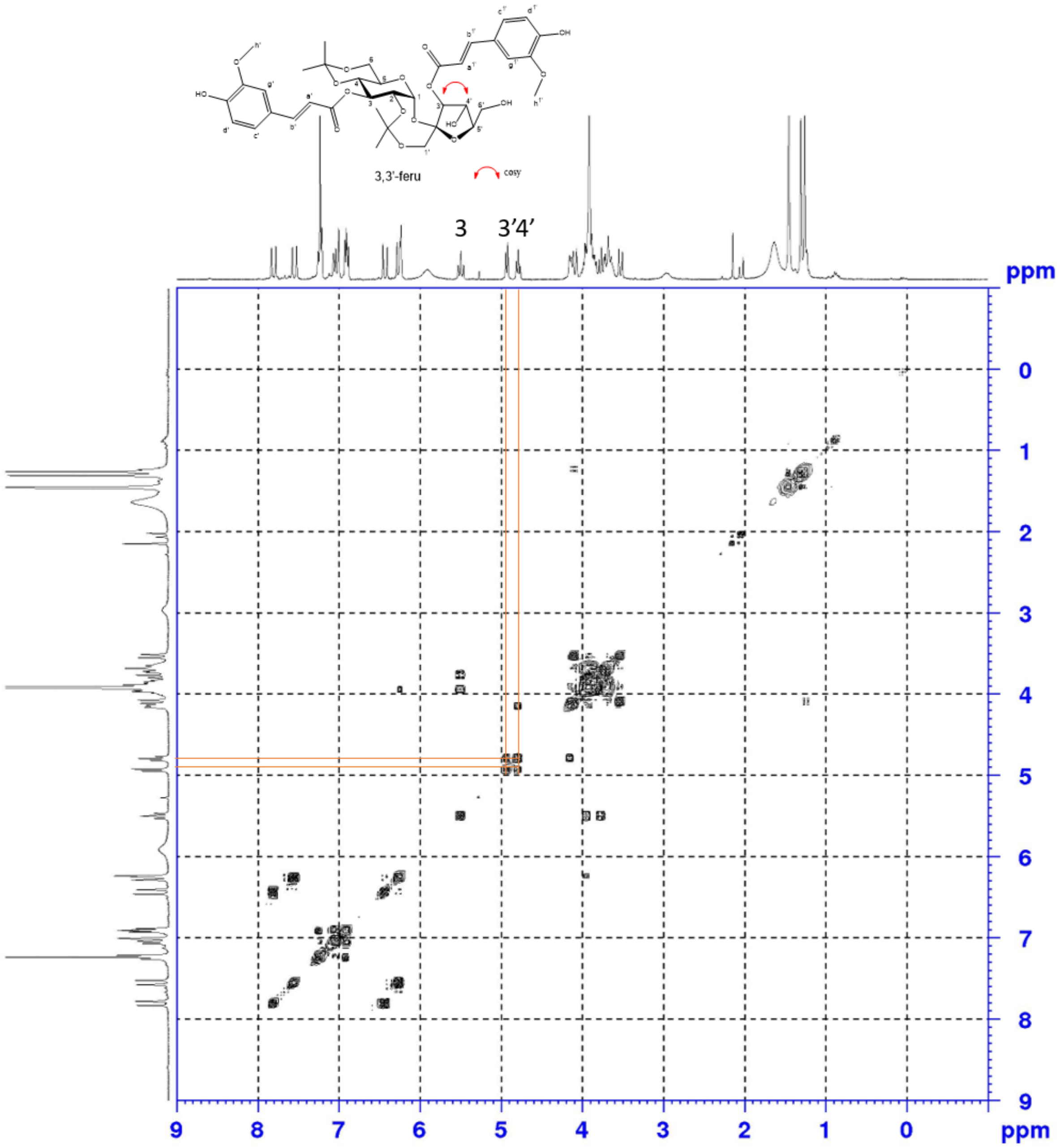

COSY spectra of **21**

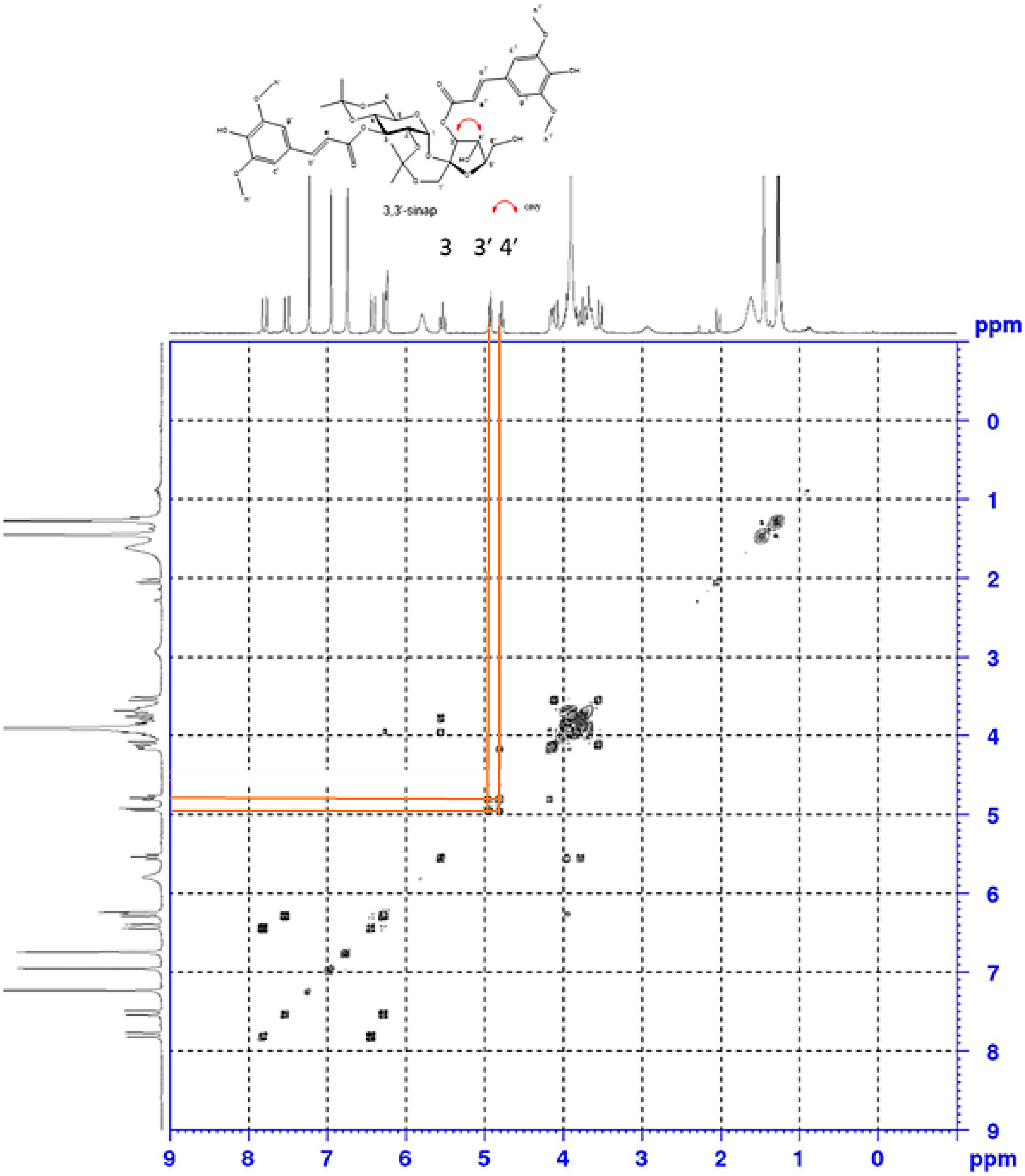

COSY spectra of **22**

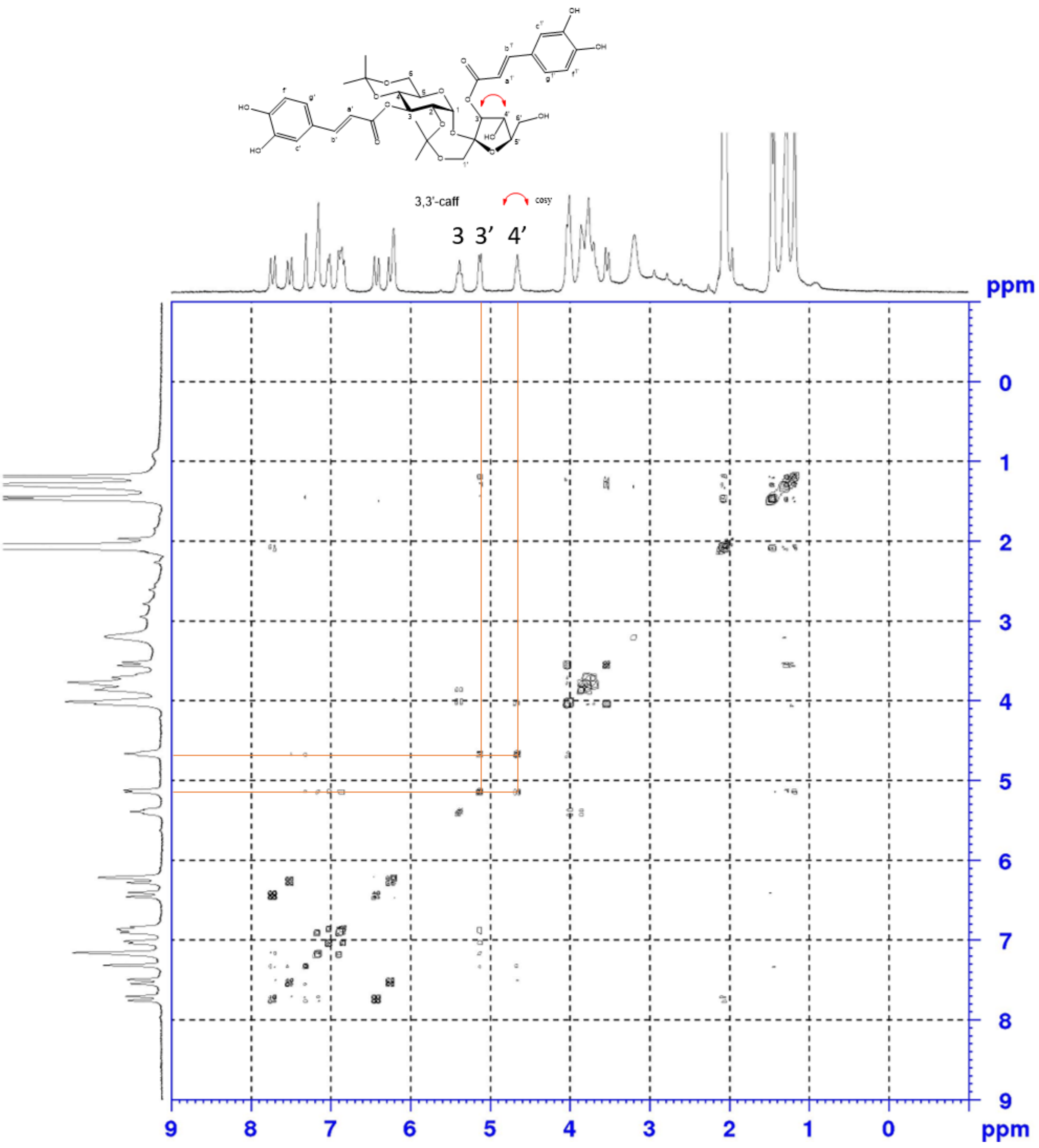

COSY spectra of **23**

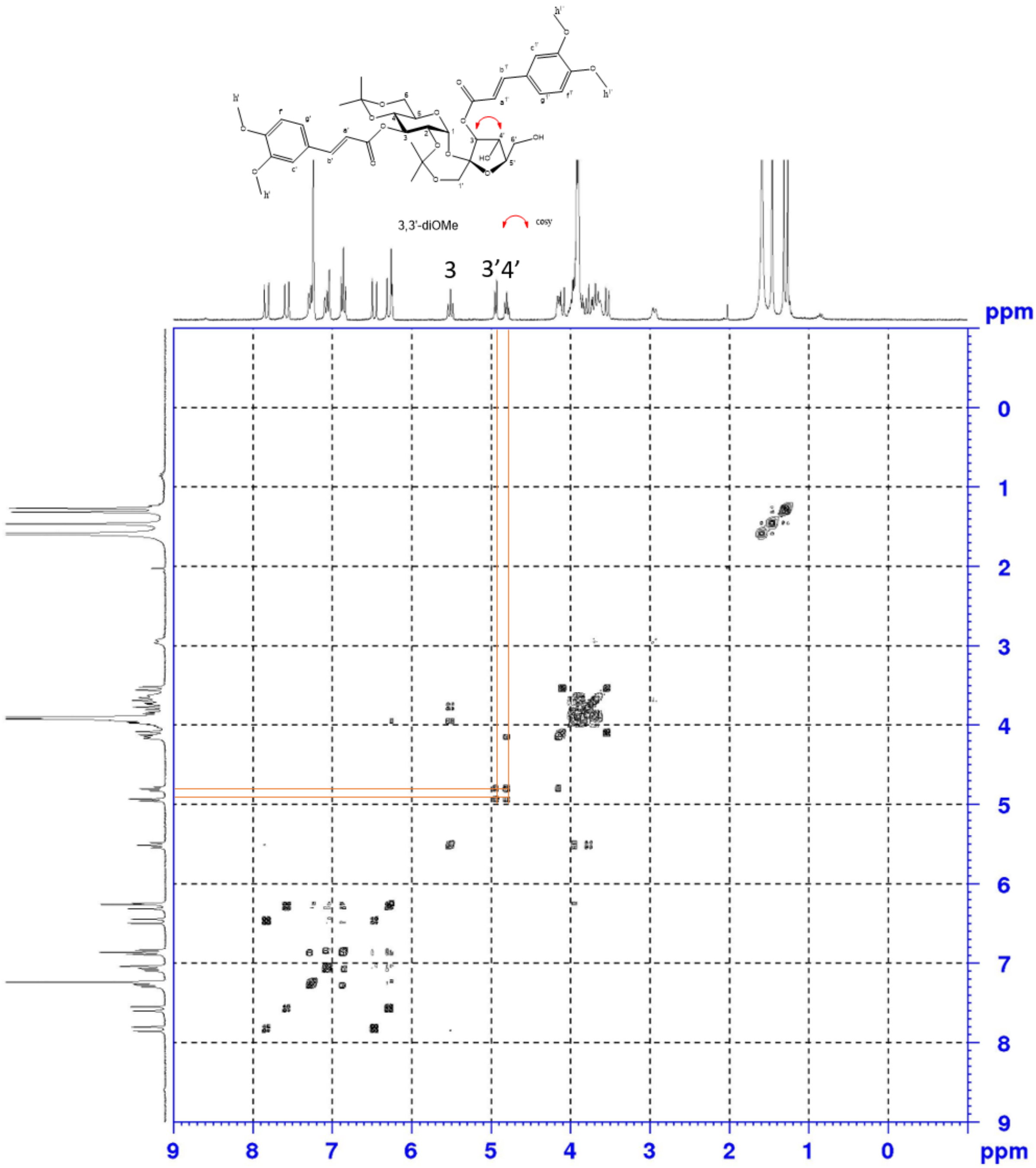

COSY spectra of **24**

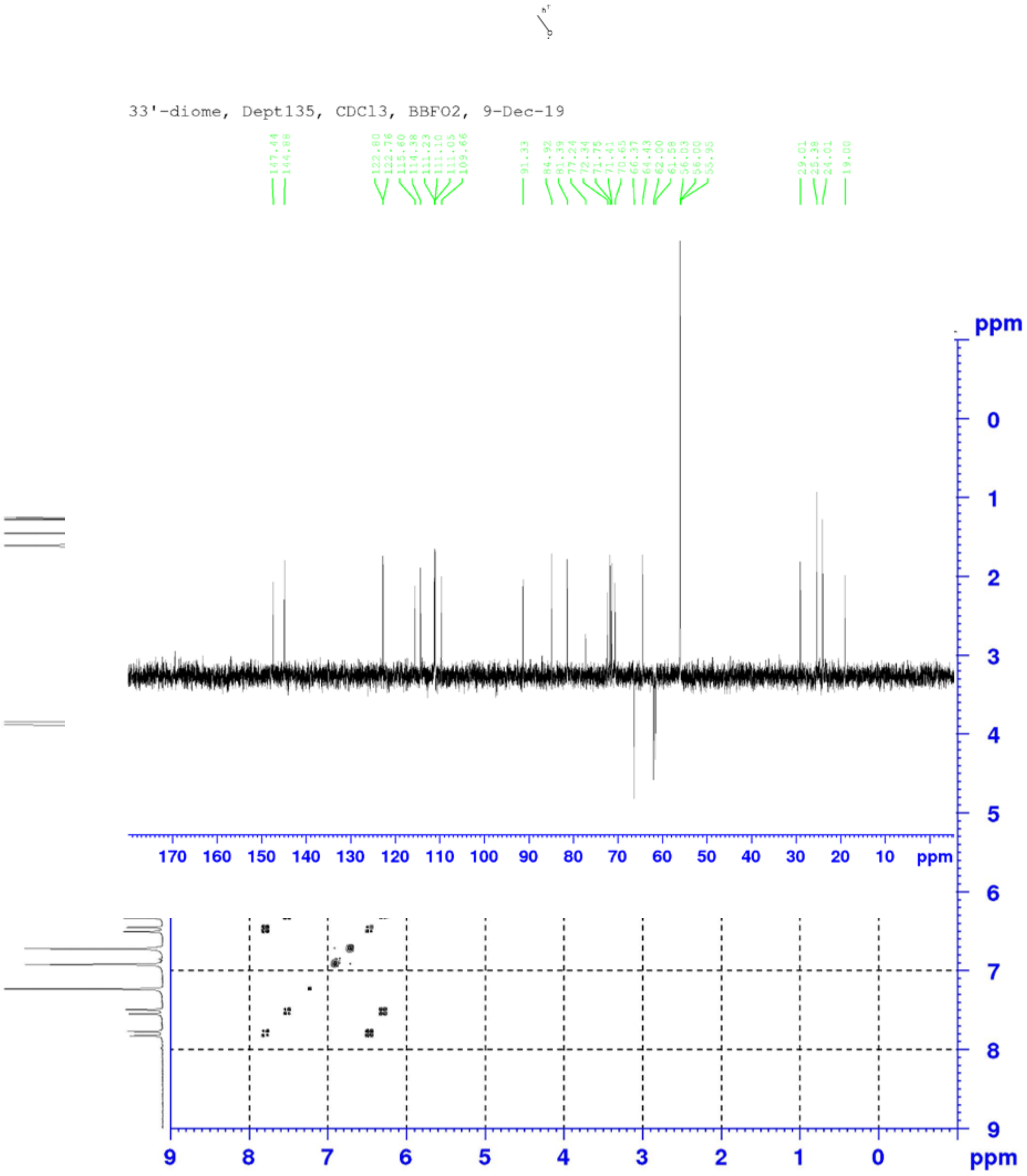

Dept135 spectra of **23**

HMBC spectra of **23**

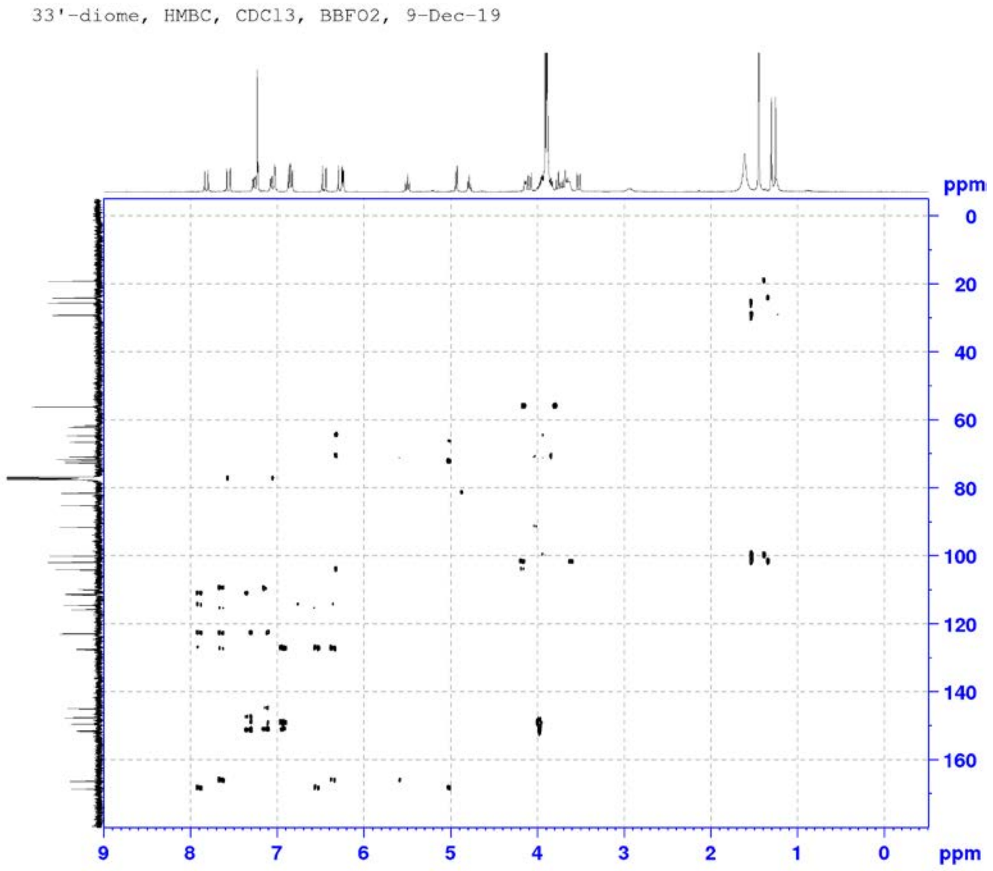

HSQC spectra of **23**

Dept135 spectra of **43**

HMBC spectra of **43**

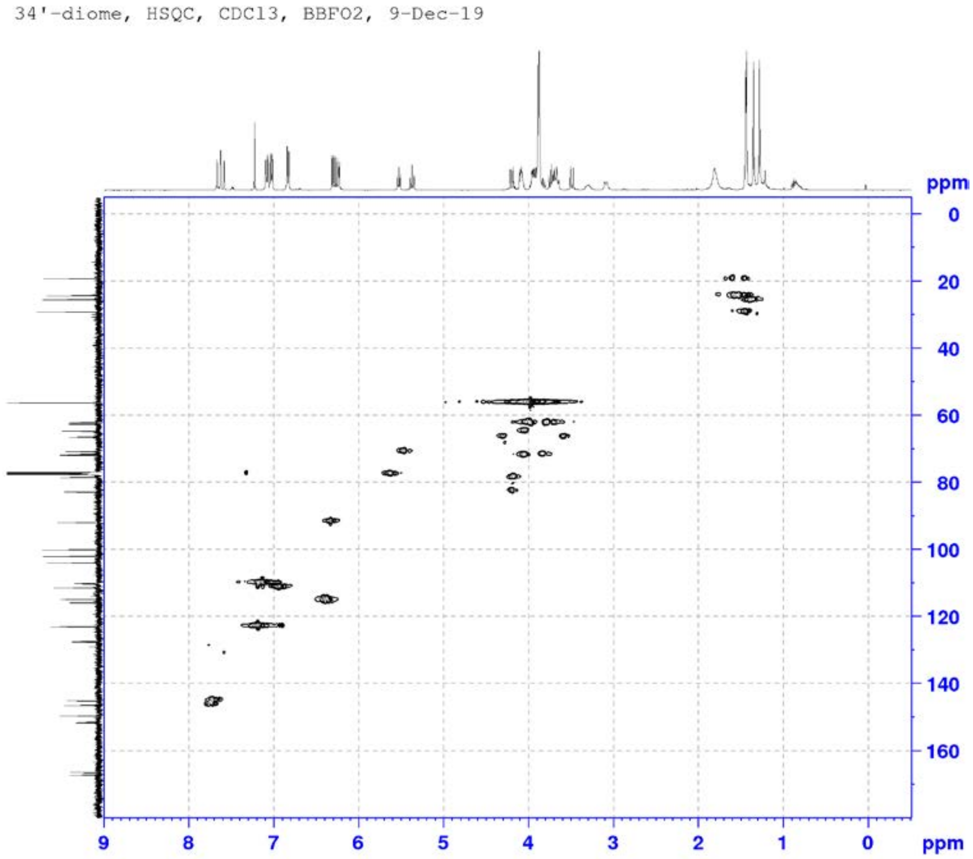
34'-diome, HSQC, CDCl3, BBFO2, 9-Dec-19
ppm
0
20
40
60
80
100
120
140
160
9
8
7
6
5
4
3
2
1
0
ppm

HS[illegible]

Dept135 spectra of **59**

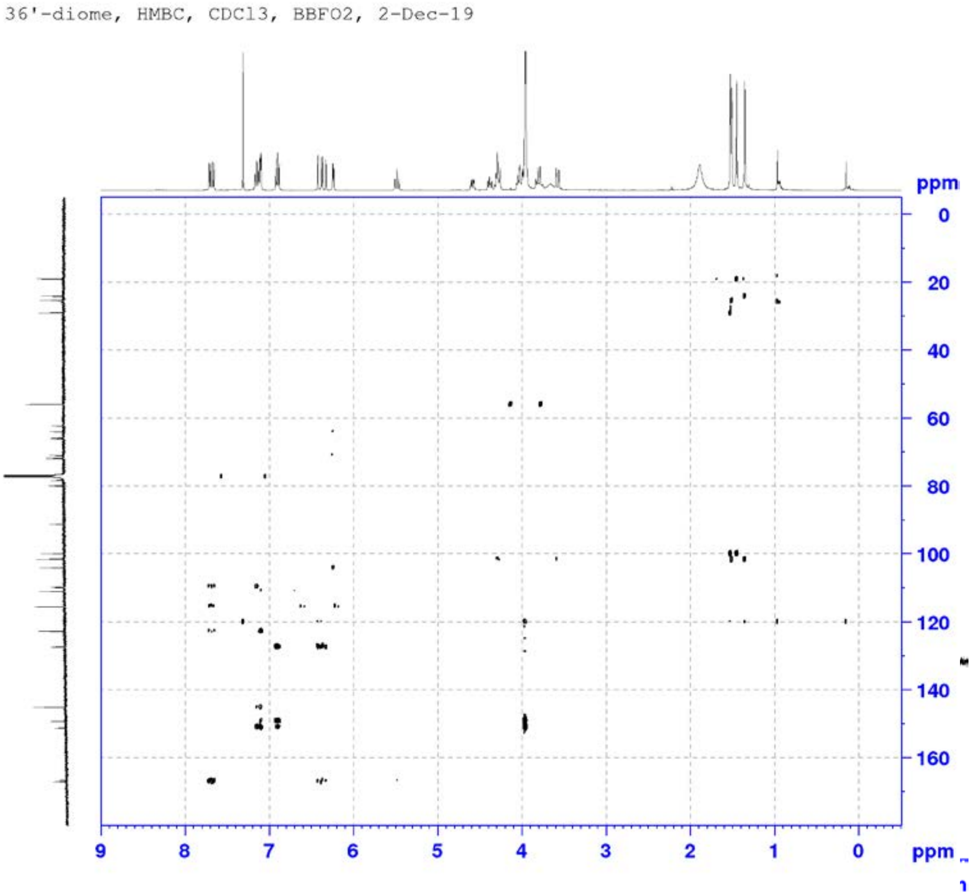

HMBC spectra of **59**

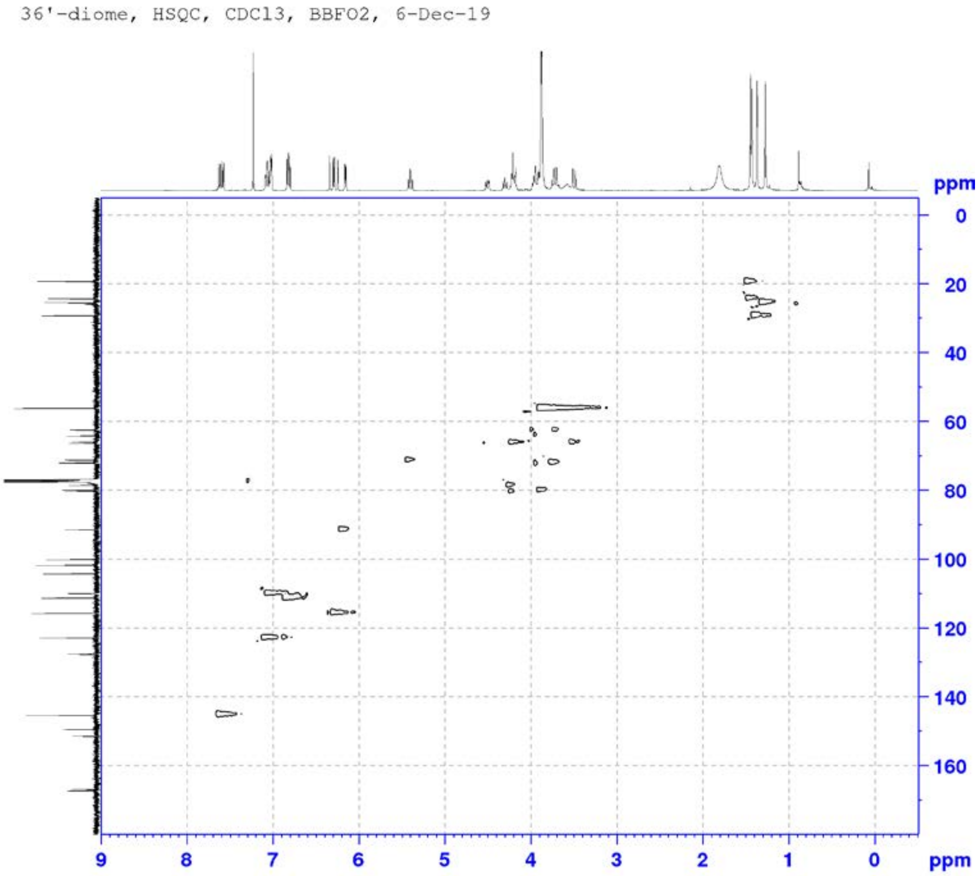

HSQC spectra of **59**

References

1. Vanderbilt, TLC stain recipes, http://www.vanderbilt.edu/AnS/Chemistry/Rizzo/stuff/TLC.html.
2. Panda, P.; Appalashetti, M.; Judeh, Z. M., Phenylpropanoid sucrose esters: plant-derived natural products as potential leads for new therapeutics. *Curr Med Chem* **2011,** *18* (21), 3234-51.
3. Lin, O. L. Development of phenylpropanoid sucrose esters as antidiabetic lead compounds. Nanyang Technological University, Interdisciplinary Graduate School (NTU Institute for Health Technologies), 2018.
4. Panda, P.; Appalashetti, M.; Natarajan, M.; Chan-Park, M. B.; Venkatraman, S. S.; Judeh, Z. M. A., Synthesis and antitumor activity of lapathoside D and its analogs. *European Journal of Medicinal Chemistry* **2012,** *53*, 1-12.
5. Panda, P.; Appalashetti, M.; Natarajan, M.; Mary, C.-P.; Venkatraman, S. S.; Judeh, Z. M. A., Synthesis and antiproliferative activity of helonioside A, 3′,4′,6′-tri-O-feruloylsucrose, lapathoside C and their analogs. *European Journal of Medicinal Chemistry* **2012,** *58*, 418-430.
6. Poschalko, A.; Rohr, T.; Gruber, H.; Bianco, A.; Guichard, G.; Briand, J.-P.; Weber, V.; Falkenhagen, D., SUBPOL: A Novel Sucrose-Based Polymer Support for Solid-Phase Peptide Synthesis and Affinity Chromatography Applications. *Journal of the American Chemical Society* **2003,** *125* (44), 13415-13426.
7. Sato, K.-i.; Sakai, K.; Tsushima, K.; Akai, S., The first total synthesis of telephiose A. *Tetrahedron Letters* **2007,** *48* (21), 3745-3748.
8. Dongxin Zhao, Li Ma, Kui Lu*and Juan He, Solid Phase Syntheses of Ferulic Acid Derivatives Acetyl Feruloyl Tyrosine and Acetyl Feruloyl Valyl Tyrosine. *The Open Biotechnology Journal* **2014,** *9*, 1-5.
9. Allais, F.; Martinet, S.; Ducrot, P.-H., Straightforward Total Synthesis of 2-O-Feruloyl-l-malate, 2-O-Sinapoyl-l-malate and 2-O-5-Hydroxyferuloyl-l-malate. *Synthesis* **2009,** *2009* (21), 3571-3578.
